# Predicting Ionic Conductivity of Imidazolium-Based Ionic Liquid Mixtures Using Quantum-Mechanically Derived Partial Charges in the Condensed Phase

**DOI:** 10.1021/acs.jpcb.4c08275

**Published:** 2025-02-21

**Authors:** Ashutosh Kumar Verma, Amey S. Thorat, Jindal K. Shah

**Affiliations:** School of Chemical Engineering, Oklahoma State University, Stillwater, Oklahoma 74078, United States

## Abstract

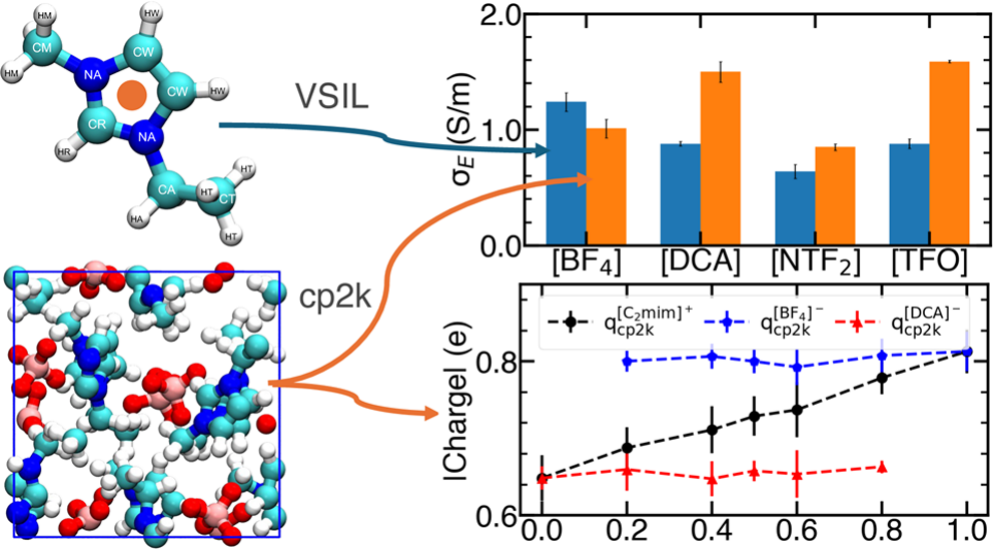

A considerable effort has been expended over the years to tune the properties of ionic liquids (ILs) by designing cations, anions, and pendant groups on the ions. A simple and effective approach to altering the properties of ILs is formulating IL–IL mixtures. However, the measurements and properties of such mixtures lag considerably behind those of pure ILs. From a molecular simulation point of view, binary IL mixtures have been investigated using charge distributions of pure ILs, which implicitly assumes that the ions of different polarizability do not influence the local electronic environment due to changing concentrations. To understand this effect, molecular dynamics (MD) simulations were conducted for a series of IL–IL mixtures containing the common cation 1-ethyl-3-methylimidazolium [C_2_mim] varying the composition of various combinations of anions (tetrafluoroborate [BF_4_] and dicyanamide [DCA], [BF_4_] and bis(trifluoromethanesulfonyl)imide [NTF_2_], [BF_4_] and trifluoromethanesulfonate [TFO], and [TFO] and [NTF_2_]). The effect of changing the electronic environment was evaluated by deriving partial charges using density functional theory (DFT) calculations in the condensed phase. It was observed that the overall charge on the cation and anion was a function of the cation–anion pairings for pure ILs. Moreover, the cation charge was found to vary linearly with anionic concentrations. Improved agreement of predicted density and ionic conductivity with experimental values was found for binary IL mixtures with this approach, in comparison to that when a fixed charge model is employed.

## Introduction

Ionic liquids (ILs) are versatile low-melting salts widely utilized in various fields, including organic^1^ and inorganic^2^ synthesis, catalysis,^3^ energy applications,^4^ gas separations,^5^ etc. Their distinctive properties, such as low volatility, conductivity, and high chemical and electrochemical stability, make them attractive designer solvents.^6^ Although ILs are ideal candidates for their application in electrochemical processes, the exploitation of ILs is hindered because ILs often exhibit low to moderate ionic conductivities. A traditional approach to alleviating this challenge is through a careful design of the cation, anion, and substituent groups on ions. In addition, the mixing of two or more ILs has the potential to boost ionic conductivity with molar ratios of the constituent ILs as a lever. This enhancement results from increased mobile ion concentration due to IL mixing and interactions among IL components that modify the local environment and improve the ion motion dynamics for more efficient ion transport. The choice of ILs for mixing is pivotal, considering chemical compatibility, miscibility, and desired properties. Careful selection of IL components and concentrations enables the customization of the ionic conductivity for IL mixtures, as demonstrated in the machine learning work by Dhakal and Shah, with the possibility that numerous IL mixtures exhibiting ionic conductivity maxima or minima as the molar ratio of constituent ILs is varied.^7^ Moreover, binary IL mixtures have been shown to suppress crystallization temperature and improve electrochemical properties.^8^

Numerous studies have extensively investigated the structure,^9−11^ thermodynamics,^12,13^ and transport behavior^14,15^ of IL mixtures. Extensive experimental^15−21^ and theoretical^10,22−25^ investigations, predominantly focused on imidazolium-based ILs, have demonstrated the crucial role of ion type and composition in determining their properties. These studies, including those by Matthews et al.,^9^ Dhakal et al.,^26^ Otero-Mato et al.,^22^ and Kapoor et al.,^13^ reveal the significant influence of both cation and anion contributions to specific properties, such as ionic conductivity. Voroshylova et al.^27^ observed that small anions, such as tetrafluoroborate ([BF_4_]^−^) or hexafluorophosphate ([PF_6_]^−^), tend to displace larger anions, like bis(trifluoromethanesulfonyl)imide ([NTF_2_]^−^), and approach closer to the cation. Wang et al.^28^ conducted MD simulations of IL mixtures with equimolar proportions of 1-ethyl-3-methylimidazolium ([C_2_mim]^+^) and 1-butyl-3-methylimidazolium ([C_4_mim]^+^) cations with [BF_4_]^−^ anions. They discovered that the imidazolium alkyl chain lengths does not significantly affect intermolecular interactions with [BF_4_]^−^. Still, they lead to slight differences in diffusion rates due to stronger hydrogen bonding with [C_4_mim]^+^. A recent study by Lu et al.^29^ explored how anion size affects association in binary IL mixtures. Their experiments revealed that smaller anions form more ion clusters, promoting association within the mixtures. Interestingly, these mixtures displayed higher conductivity than the pure ILs despite having a lower degree of association themselves.

These findings offer valuable insights into the fundamental intermolecular interactions that are critical for computing the ionic conductivity of IL mixtures. Molecular-level simulations significantly advanced our understanding of IL mixture complexities.^9,25^ Matthews et al.^9^ and Kapoor and Shah^25^ showed preferential interactions between cations and anions with varying hydrogen bonding abilities in mixtures. Additionally, binary IL mixtures have been reported to exhibit nonideal behavior in dielectric constant, with some mixtures showing values nearly twice that of the pure liquids.^30^ Recently, Wang et al.^31^ employed molecular dynamics simulations to investigate the structure, dynamics, and hydrogen bonding in [C_2_mim][BF_4_][NTF_2_] mixtures. Their results showed a weakening interaction between cations and both anions with an increasing [BF_4_]^−^ concentration. Additionally, all ions exhibited faster diffusion at higher [BF_4_]^−^ concentration, with the order of diffusion coefficients being [C_2_mim]^+^ > [BF_4_]^−^ > [NTF_2_]^−^, independent of composition.

Computer simulations are useful for studying phenomena that are difficult to observe through experiments and for predicting properties of interest. However, a suitable force field is required for the system being studied. The use of scaled-charge force fields is widespread in simulating pure ILs. In these force fields, the charges on the ions are adjusted empirically (scaled between 0.7 and 1.2) to approximately represent electronic polarization and charge transfer. This charge scaling has been shown to significantly improve the thermodynamic and transport properties of the liquid state when compared to experimental data.^32,33^ However, the mean-field approximation involved in charge scaling raises concerns when applied to IL mixtures or solutions.^34^

Furthermore, the challenge lies in the inherent variability of IL charges due to factors like charge transfer between ions and polarization from the surrounding environment.^35^ Consequently, developing a separate force field for the IL mixtures is crucial. Ab initio and density functional theory (DFT) can provide precise intra- and intermolecular interaction information for ILs. However, these methods are computationally demanding, typically limiting their application to small systems and short time scales. In contrast, MD simulations based on classical force fields can handle systems with hundreds or thousands of IL pairs over nanoseconds or microseconds. The reliability of such simulations depends heavily on the quality of the force fields, which are typically developed through a combination of quantum calculations and comparsion with experimental data. Several studies have focused on developing force-field parameters for ILs. Canongia Lopes and co-workers used OPLS-AA and Amber to generate force fields for imidazolium-based ILs, while Wang and co-workers developed force fields for imidazolium-based ILs using Amber.^36−40^ Later, Doherty et al. introduced a new force field, OPLS-VSIL, designed explicitly for imidazolium-based pure ILs.^33^ Estimation of ionic conductivity is highly sensitive to the force field parameters. For example, minute differences in the values of nonbonded Lennard-Jones parameters (σ and ϵ) have been shown to yield substantially different conductivity values or sometimes highly agglomerated unphysical representations of the system.^41^ Hence, it is necessary to exercise caution when optimizing force fields solely based on ionic conductivity.

Unlike most works that focus on optimizing force fields through DFT-based partial atomic charges for the prediction of the thermophysical properties of pure ILs, this work focuses on estimating the ionic conductivity of IL mixtures. This work also takes into account the effect of mixture composition on the magnitude of the partial atomic charges, thereby accommodating the polarization effect due to mixing. We applied quantum mechanical DFT calculations to obtain the partial atomic charges of [C_2_mim][BF_4_][DCA], [C_2_mim][BF_4_][NTF_2_], [C_2_mim][BF_4_][TFO], and [C_2_mim][TFO][NTF_2_] in their bulk liquid phases. These bulk charges enable the investigation of ionic conductivity in binary IL mixtures containing the [C_2_mim]^+^ cation and varying mole fractions of [BF_4_]^−^, [DCA]^−^, [NTF_2_]^−^, and [TFO]^−^ anions along with their neat counterparts. We compare the performance of three charge calculation strategies for predicting ionic conductivity in binary IL mixtures. To achieve this, all force field parameters except the partial atomic charges were kept constant. The charges were manipulated using (a) a virtual site model developed by Doherty et al.,^33^ (b) cp2k-charges obtained from condensed phase DFT calculations for trajectories with a given mole fraction of anions, and (c) cation charges linearly interpolated based on the charges obtained for pure ILs from condensed phase DFT calculations while the charge on the anion obtained for pure IL was retained for mixtures.

## Methodology and Simulation Details

### Force Field Details

An OPLS-based force field developed by Doherty et al.^33^ was used to model intermolecular interactions. Nonbonded interactions were modeled using Lennard–Jones (LJ) 12–6 and electrostatic potential. Mathematically, the nonbonded potential can be expressed as follows:
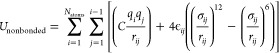
1where *N*_atoms_ is the number of atoms in the system, *C* is the Coulomb constant, *q*_*i*_ and *q*_*j*_ are charges of *i*th and *j*th atoms, σ_*ij*_ and ϵ_*ij*_ are the LJ parameters for the cross interactions between the *i*th and *j*th atoms in the system, respectively, and *r*_*ij*_ is the separation between the atoms. The geometric combining rules, i.e., σ_*ij*_ = (σ_*ii*_σ_*jj*_)^1/2^ and ϵ_*ij*_ = (ϵ_*ii*_ϵ_*jj*_)^1/2^ were used in this work. The intramolecular interactions between atoms separated by exactly three bonds were scaled by a factor of 0.5.

### MD Simulation Details

**Figure 1:**
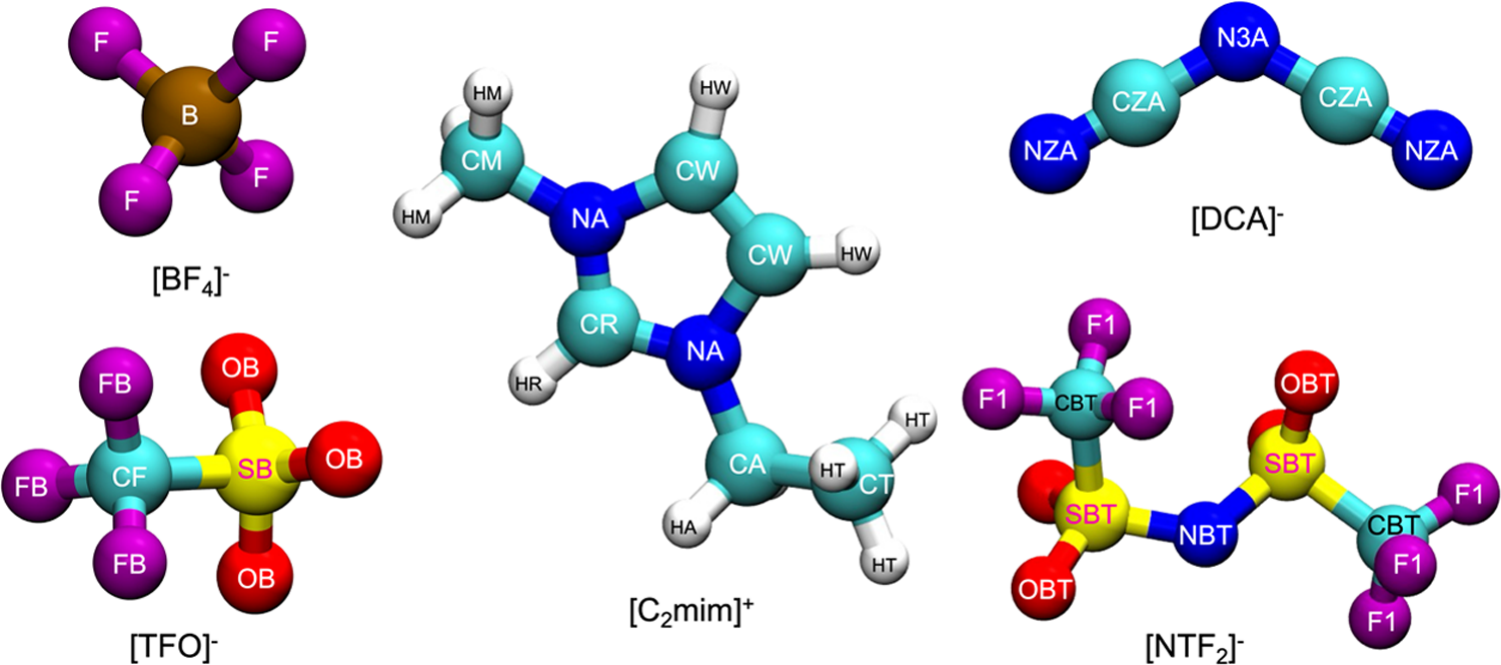
Molecular structure of one cation ([C_2_mim]^+^) and four anions ([BF_4_]^−^, [DCA]^−^, [NTF_2_]^−^, and [TFO]^−^). The colors cyan, white, blue, yellow, red, purple, and dark brown represent carbon, hydrogen, nitrogen, sulfur, oxygen, fluorine, and boron, respectively.

GROMACS 2018 software package was used to conduct MD simulations in the canonical (*NVT*) and isothermal–isobaric (*NPT*) ensembles.^42^ The simulation systems consisted of cubic boxes containing 500 ion pairs, which were generated using Packmol.^43^ The structures of the ions are depicted in Figure 1. Simulation box lengths of pure ILs for initial molecular configurations were obtained from Doherty et al.,^33^ and simulation box lengths were set slightly larger for the IL mixtures. All simulation boxes were periodic in the *x*, *y*, and *z* directions. Initially, an energy minimization was carried out using the steepest descent algorithm for 5000 steps. Subsequently, an annealing process was conducted over 1.5 ns, during which the reference temperature was precisely adjusted. Equilibration runs were continued for 35 ns, starting with a 5 ns *NVT* (298 K) equilibration run using a Berendsen thermostat with a time constant of 1 ps.^44^ This was followed by a 20 ns *NPT* (298 K and 1 bar) equilibration run using the v-rescale thermostat with a time constant of 1 ps and the Berendsen barostat with a time constant of 1 ps.^45^ Another 10 ns *NPT* (298 K and 1 bar) equilibration run was performed using the Nosé–Hoover thermostat^46^ with a time constant of 2 ps and the Parrinello–Rahman barostat^47^ with a time constant of 10 ps.

Finally, *NPT* (298 K and 1 bar) production runs were conducted for 100 ns, with trajectory data collected at 5 ps intervals. The Nosé–Hoover thermostat with a time constant of 2 ps and the Parrinello–Rahman barostat with a time constant of 10 ps were used in the simulation. The LJ cutoff was fixed at 13.0 Å, and the long-range contributions to energy and pressure due to LJ interaction were handled by enabling dispersion correction terms. The electrostatic interactions were decomposed into short-range and long-range interactions. For short-range electrostatic interactions, pairwise interactions were summed, and Particle-Mesh Ewald summations were employed to account for long-range electrostatic interactions with a 13 Å cutoff distance.^48^ The equations of motion were integrated using the leapfrog algorithm with a time step of 1 fs. The covalently attached hydrogen bonds were constrained using the LINCS algorithm.^49^ Three independent simulations were performed. Each simulation employed a unique random seed, allowing for the generation of distinct initial atomic arrangements. The mean and standard deviations of the simulated properties were determined from these three independent calculations.

### Details of the Ionic Conductivity Calculations

The Einstein relation for self-diffusion coefficients of *k*th ionic species is defined as,
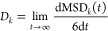
2The MSD, over a time interval *t*, is defined as
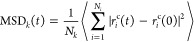
3Here, *N*_*k*_ denotes the number of *k*th ionic species and *r*_*i*_^c^ is the location of the center-of-mass of the ions. The ⟨···⟩ value indicates the ensemble average. The Nernst–Einstein (NE) equation (eq 4) establishes a relationship between the diffusion coefficients and ionic conductivity, which are essential properties for understanding ion transport in ILs:
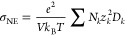
4Here, *e* represents the elementary charge, *V* is the volume of the system, *k*_B_ denotes the Boltzmann constant, and *T* is the system temperature. *N*_*k*_, z_*k*_, and *D*_*k*_ indicate the number, charge, and self-diffusion coefficient of the *k*th ionic species, respectively. In NE-based calculations, it is assumed that the motion of ions within the system is unaffected by the presence of other ions. This assumption disregards ion correlation, and as a result, the ionic conductivity estimated using the NE equation provides an upper limit. This concept is particularly applicable in situations involving dilute solutions or systems, where the assumption of ions moving independently is reasonably valid. On the other hand, Einstein formalism proves more advantageous in scenarios involving concentrated electrolyte systems or instances where ions exhibit strong interactions with each other. This approach considers the impacts of ion–ion interactions, which can substantially influence the ionic conductivity. Recently, we outlined a comprehensive workflow for utilizing Einstein formalism, providing valuable guidance for accurate ionic conductivity computations for ionic liquids.^50^ Mathematically, the Einstein conductivity is calculated as follows:
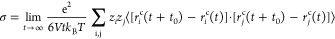
5When we focus solely on self-terms (*i* = *j*), the Einstein conductivity equation can be transformed into the NE equation (eq 4).

### Derivation of Partial Atomic Charges

Quantum mechanical density functional theory calculations were used to calculate the partial atomic charges. To calculate the interpolated-charges for the cation using pure IL charges, we employed the formula: *q*_*i*,mix_ = *xq*_*i*,IL-1_ + (1 – *x*)*q*_*i*,IL-2_. In this formula, *q*_*i*,mix_ denotes the charge of the *i*th atom in the mixture, *x* signifies the composition, *q*_*i*,IL-1_ represents the charge of the *i*th atom in IL-1, and *q*_*i*,IL-2_ represents the charge of the *i*th atom in IL-2. To accurately represent ionic interactions in condensed phase simulations, atomic charges can be derived using the simple AIMD technique developed by Zhang and Maginn.^35^ Their work explored four distinct charge sets, including those obtained from gas-phase quantum mechanical calculations (integer charges) and those derived from liquid-phase AIMD simulations. While a similar methodology was employed earlier by Wendler et al.,^51^ they considered uncorrelated configurations equilibrated using classical MD simulations. Subsequently, they performed wave function optimization, ensuring that the maximum force applied to each atom was 0.004 au. Atomic charges were then computed using Blöchl analysis.^52^ Later, Ishizuka and Matubayasi^53^ developed a similar methodology called self-consistent (iterations of MD followed by bulk DFT calculation) determination of atomic charges. Their work focused on [C_1_mim][NTF_2_] and revealed that the net charges on ions remain consistent with the temperature. Further, Ishii and Matubayasi^54^ extended the same MD-DFT self-consistent methodology to imidazolium-, pyrrolidinium-, and ammonium-based ILs.

There is, however, no unique method for partitioning the electron density into atomic site charges, and numerous charge partitioning schemes have been proposed for IL systems. These include the restrained electrostatic potential (RESP),^55^ repeating electrostatic potential extracted atomic (REPEAT),^56^ grid-based methods like ChelpG,^57,58^ the density-derived atomic point (DDAP) scheme, also known as the Blöchl analysis,^52^ and the density-derived electrostatic and chemical (DDEC) method.^59^ Balasubramanian and co-workers refined the CL&P force field parameters by deriving atomic charges using the DDEC method.^60,61^ They found that the DDEC charge scheme resulted in consistent charges on the [C_n_mim] cations, even with varying alkyl chain lengths. In another study, Matubayasi and co-workers^53,54^ used the Blöchl analysis to compute atomic charges for the IL [C_1_mim][NTF_2_]. However, these studies are limited to pure ILs and do not address the complexities involved in IL mixtures, where the charge distribution can differ significantly due to interactions between multiple ionic species. Here, we extend the MD-DFT self-consistent methodology to imidazolium-based binary IL mixtures. The detailed method and protocol are provided in the Supporting Information, under Section S1. First, the initial configurations were equilibrated using MD simulations. Subsequently, liquid-phase DFT calculations were performed utilizing the cp2k software package.^62^ The partial atomic charges were then derived through the DDAP scheme^52^ implemented in the cp2k software. In the DDAP method, the charge density on the atoms is represented using a Gaussian, and the long-range electrostatic potential is used to fit the partial charges. The generalized gradient approximation (GGA) Perdew–Burke–Ernzerhof (PBE) exchange-correlation functional^63^ was used to calculate the electronic energy, and the valence electrons in atoms were represented using the molecularly optimized BASIS–MOLOPT basis set.^64^ The nuclei and core electrons were represented using Norm–conserving Goedecker–Teter–Hutter (GTH) pseudopotentials.^65^ A 450 Ry plane wave cutoff was used. Dispersion interactions were incorporated using Grimme’s dispersion D3 model.^66^

## Results and Discussion

### Atomic Charges

**Figure 2:**
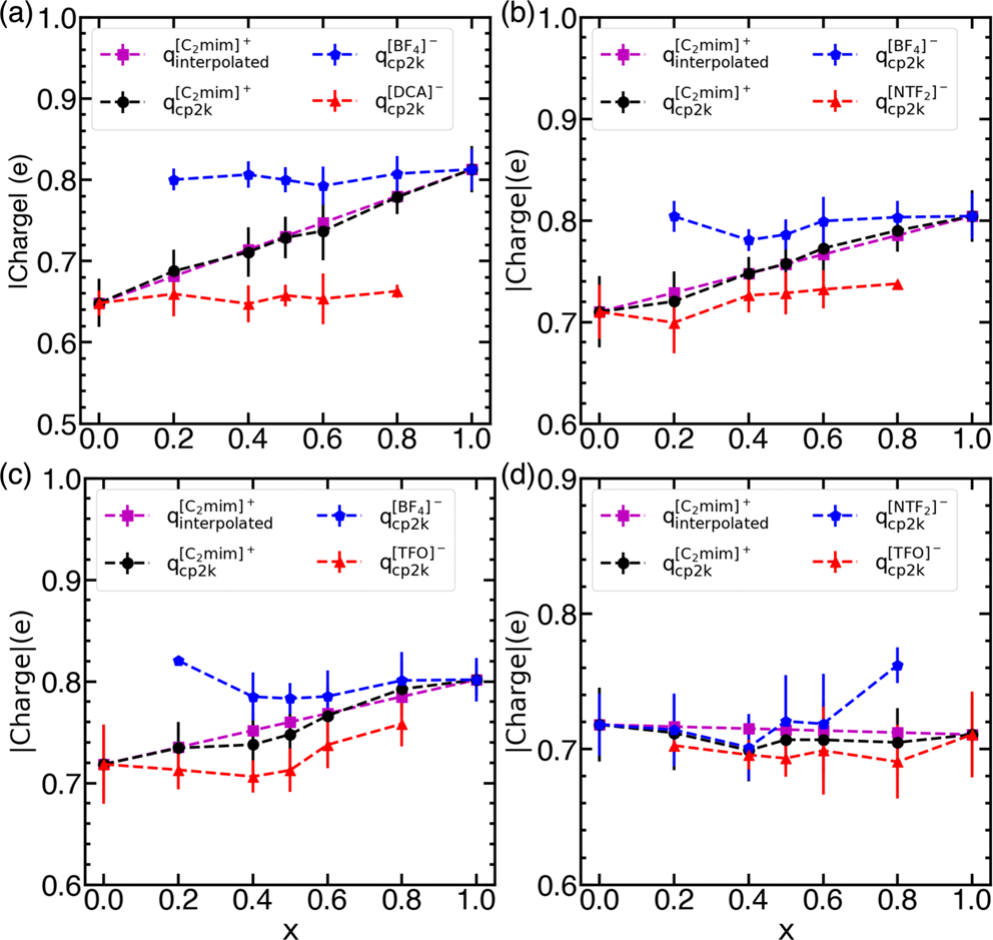
Net charges on cations and anions as a function of anion mole fraction. (a) [C_2_mim][BF_4_][DCA], (b) [C_2_mim][BF_4_][NTF_2_], (c) [C_2_mim][BF_4_][TFO], and (d) [C_2_mim][TFO][NTF_2_] mixture. The charges were derived based on liquid-phase DFT calculations and the DDAP scheme proposed by Blöchl.^52^ The dashed lines act only as a visual guide.

The overall charges on the cation and anion obtained from DFT calculations are plotted in Figure 2 for four different IL mixtures as a function of the IL composition. For pure ILs, we observed that the net charge on the ions varied with the identity of the anion and followed the trend: 0.81 ([BF_4_]^−^) > 0.72 ([NTF_2_]^−^) > 0.71 ([TFO]^−^) > 0.65 ([DCA]^−^), which differs markedly from unity, as observed in many gas-phase calculations due to charge transfer. In the present case, the lowering of the net charge is also due to the presence of other ions and thus represents a combined effect of charge transfer and polarization.^54,61,67−69^

Mondal and Balasubramanian^61^ deduced a similar decrease in the total ion charge from unity, calculated for both the crystal and its corresponding liquid, reflecting the effect of polarization on individual ions due to the surrounding medium. The considerable variation in the net charge indicates that the extent of polarization in [C_2_mim]-based ILs is strongly associated with the type of the anion, which was also reported by Mondal and Balasubramanian^61^ that the overall charge on the anion varied from −0.64 for chloride to −0.79 for [BF_4_]^−^. Later, Matubayasi and co-workers^53,54^ also observed a reduction in net charges for various ILs, ranging from 0.567 to 0.907, for imidazolium-, pyrrolidinium-, and ammonium-based ILs. A comparison of the atomic charges obtained in this work is carried out with those reported for the VSIL force field and by Mondal and Balasubramanian in their work to parametrize the IL force field by deriving partial charges from the DDEC method for [C_4_mim]^+^-based ILs (see Table S1). Surprisingly, the charges calculated in this work are nearly identical to those computed in the work by Mondal and Balasubramanian for [BF_4_]^−^, indicating that the type of the cation and the ab initio method employed for the charge assignment calculation have minimal impact on atomic charges. On the other hand, the atomic charge obtained in this work on the carbon atom in [TFO] differs considerably from that reported in the Mondal and Balasubramanian work. Similarly, the atomic charge on the nitrogen atom in this work is less negative than that reported from the DDEC method for the [NTF_2_] anion, while the carbon atom carries a lower positive charge for our model than that in the force field developed by Mondal and Balasubramanian. Furthermore, the net charges (±0.65) on ions using Blöchl analysis^52^ in [C_2_mim][DCA] are remarkably similar to those reported by Wendler et al.^51^

In a binary IL mixture, it is expected that the net charge on each type of ion would fluctuate to maintain the overall neutrality of the system. Remarkably, our calculations suggested that the net charge on the anions was almost identical across the various mole fractions at a value obtained for the respective pure IL system. The results imply that, in a binary IL mixture comprising two different anions, the net charge on the anions is only minimally impacted irrespective of the identity of the other anions. Such a tendency could be understood from the viewpoint that anion–anion interactions are primarily indirect in nature and often mediated by the cation. As a consequence of the constancy of the net charge of anions, we found that the overall charge on the cation varied between the net charges of the anions. Our calculations suggested that the overall charge on the cation could be approximated as the mole fraction-weighted charges obtained from respective pure ILs, as shown in Figure 2. These findings are well aligned with the work reported by Avula et al.,^70^ although their study focused on the charge environment and hydrogen bond dynamics in [C_4_mim]Cl[BF_4_] and [C_4_mim][NTF_2_][SCN] mixtures. As such, the cation charge varied linearly with the anion composition for the three systems [C_2_mim][BF_4_][DCA], [C_2_mim][BF_4_][NTF_2_], and [C_2_mim][BF_4_][TFO] mixtures due to the fact that there is a significant difference in the overall charge for ILs forming the binary mixture. On the other hand, the net charge for [C_2_mim]^+^ remains nearly constant with increasing [TFO]^−^ concentrations in the [C_2_mim][TFO][NTF_2_] mixture, as the net charges for pure ILs are almost equivalent. This finding enabled the derivation of atomic charges for cations through the interpolation of charges obtained from pure ILs, as shown in Figure 2. This result is quite promising as it implies that, in a binary IL mixture comprising a common cation and two anions, the charge on the cation in the mixture can be easily estimated from the charges in pure ILs, thereby eliminating the need for conducting iterative classical simulations on various IL mixtures of varying compositions and deriving charges using an ab initio method. Henceforth, we will refer to the charges obtained from DFT calculations as cp2k-charges and the charges calculated using the linear combination rule as interpolated-charges.

Relative standard deviation analysis of partial atomic charges derived from cp2k calculations (Figure S1 for visual representation) revealed remarkable consistency across all atomic sites in binary IL mixtures for various concentrations except in atoms CT, HA, and HT for the [C_2_mim][BF_4_][DCA] mixture, atoms CA, HM, CT, HA, HT, and CR for the [C_2_mim][BF_4_][NTF_2_] mixture, atoms CT, HA, HT, and CR for the [C_2_mim][BF_4_][TFO] mixture, and atoms CT, HA, and HT for the [C_2_mim][TFO][NTF_2_] mixture. It can be inferred that the presence of two different anions modifies the electronic environment around each of the atoms in the cation in a subtle way. However, as we will demonstrate later, these subtle changes in charge distribution profoundly influence the transport properties of these mixtures.

### Properties of Pure ILs

To validate the efficacy of the OPLS-based force field combined with charges employed in this work, we initially focused on predictions of density, transport properties (self-diffusion coefficients and ionic conductivity), and liquid structures of pure ILs. Employing GROMACS in-built tools, the average densities were calculated across independent production runs. Self-diffusion coefficients and ionic conductivity were estimated using Python scripts developed in-house.^50^

#### Density

Figure S2 compares the computed densities from our simulations with weighted average experimental data from the literature; numerical values are collected in Table 1. Also depicted in the figure are density predictions from the VSIL force field. It is evident that the density estimates from cp2k-charges are in excellent agreement with the VSIL predictions and experimentally reported values, which conveys that the packing of ions is relatively unaffected by different charge scaling. However, small differences in the density predictions from the two force fields are apparent as a result of different scaling of charges and the absence of a virtual site in the cp2k-charge force field. For example, the predicted density for [C_2_mim][BF_4_] is slightly higher using cp2k-charges than VSIL due to the increased overall charge for the IL, which enables stronger hydrogen bonding interactions (Figure S24(d)). On the other hand, a decrease in the density is noted with cp2k-charges for [C_2_mim][DCA], [C_2_mim][TFO], and [C_2_mim][NTF_2_], which can be attributed to a lowering of the electrostatic interactions due to the reduced overall charge. In fact, revising the charges leads to an improvement agreement of density predictions with experimental data for [C_2_mim][TFO] and [C_2_mim][DCA] ILs. The trends in density variations with scaling charges are in line with those reported in the literature.^71^

**Table 1:** Densities (g/cm^3^) Obtained in this Work Using the cp2k-Charges and VSIL Force Field^33^^,^^a^

IL	cp2k-charges	VSIL	VSIL^a^	exp^a^
[C_2_mim][BF_4_]	1.284	1.277	1.278	1.278
[C_2_mim][DCA]	1.136	1.152	1.149	1.103
[C_2_mim][NTF_2_]	1.595	1.599	1.579	1.517
[C_2_mim][TFO]	1.431	1.455	1.456	1.384

a Densities calculated using the VSIL force field and reported in a study by Doherty et al.^33^

#### Self-Diffusion Coefficients

**Figure 3:**
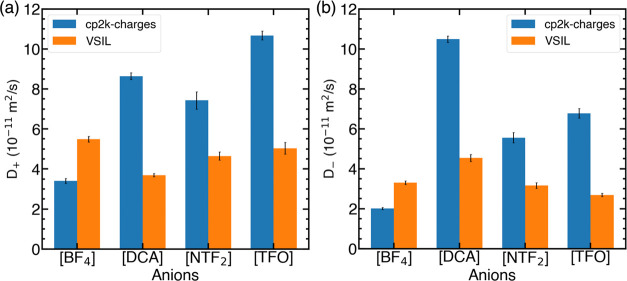
Self-diffusion coefficients of ions in pure ILs ([C_2_mim][BF_4_], [C_2_mim][DCA], [C_2_mim] [NTF_2_], and [C_2_mim][TFO]) at 298 K (a) cation and (b) anion using the cp2k-charges and VSIL force field.

The self-diffusion coefficients of the ions were obtained using the MSD (eq 2) calculated from the production run trajectory, as detailed in the Methodology and Simulation Details Section. A logarithmic plot of the MSD versus time (eq S1) was constructed. The self-diffusion coefficients were then estimated from the slope of the linear region of this plot (see Supporting Information, Section S2.2), following the approach employed by Thorat and Shah.^41^Figure 3 portrays self-diffusion coefficients of cations and anions in pure ILs ([C_2_mim][BF_4_], [C_2_mim][DCA], [C_2_mim][NTF_2_], and [C_2_mim][TFO]) using cp2k-charges and the VSIL force field. It is evident that the self-diffusion coefficients obtained using cp2k-charges are in the same order of magnitude as those calculated with the VSIL force field. Both force fields yield self-diffusion coefficients of the cation that are higher than those for the anion, except in the case of [C_2_mim][DCA], for which an opposite trend is observed, which is consistent with previous computational^8,26^ studies. This anomalous behavior primarily stems from the smaller size and planar geometry of the [DCA]^−^ anion. For all of the ILs except [C_2_mim][BF_4_], the self-diffusion coefficients for the ions are higher for the force field based on cp2k-charges in comparison to those for VSIL, which correlates well with the density. The observation can be attributed to lower cp2k-charges on ions in [C_2_mim][DCA], [C_2_mim][NTF_2_], and [C_2_mim][TFO] than VSIL and slightly higher cp2k-charges on ions in [C_2_mim][BF_4_] than VSIL. An additional comparison of the self-diffusion coefficients determined in this work with the literature values is presented in Table S2. It can be seen that the force field based on cp2k-charges outperforms the VSIL force field for [C_2_mim][DCA] and [C_2_mim][NTF_2_] ILs, while the VSIL force field is preferable for the other two ILs.

#### Ionic Conductivity

**Figure 4:**
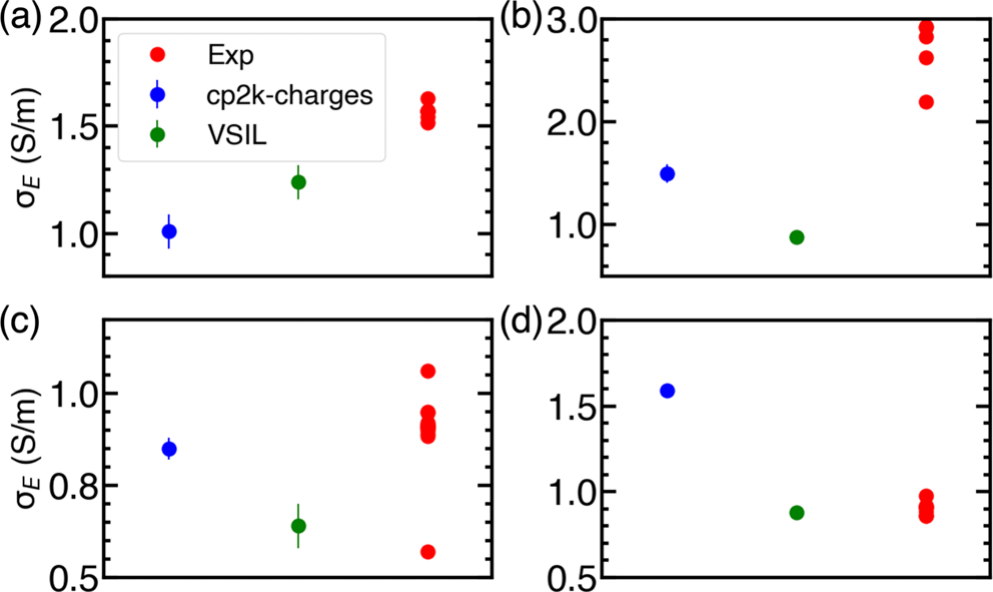
Einstein conductivity (S/m) of pure ILs (a) [C_2_mim][BF_4_], (b) [C_2_mim][DCA], (c) [C_2_mim][NTF_2_], and (d) [C_2_mim][TFO] at 298 K.

The ionic conductivity of all four pure IL was calculated using the NE (eq 4) and Einstein (eq 5) relations and tabulated in Tables S3 and S4 (Supporting Information, Section S2.3). MD simulations employing cp2k-charges and VSIL yielded a distinct conductivity trend for ILs with the [C_2_mim]^+^ cation. As expected, the NE ionic conductivity is higher than the corresponding Einstein conductivities. The predicted Einstein conductivity order for the VSIL force field was [C_2_mim][BF_4_] > [C_2_mim][DCA] ≥ [C_2_mim][TFO] > [C_2_mim][NTF_2_], while cp2k-charged resulted in a trend of ionic conductivity in the order [C_2_mim][TFO] > [C_2_mim][DCA] > [C_2_mim][BF_4_] > [C_2_mim][NTF_2_]. These predictions did not fully align with the experimentally observed order: [C_2_mim][DCA] > [C_2_mim][BF_4_] > [C_2_mim][TFO] > [C_2_mim][NTF_2_]. It is evident from Figure 4 that switching from VSIL charges to cp2k-charges results in an increase in the Einstein conductivity for [C_2_mim][DCA], [C_2_mim][TFO], and [C_2_mim][NTF_2_] while there is a decrease in the Einstein conductivity for [C_2_mim][BF_4_]. Moreover, the agreement with experimental conductivity improves for [C_2_mim][DCA], while the quantitative agreement with experimental data is obtained for [C_2_mim][NTF_2_]. On the other hand, the Einstein conductivity obtained with VSIL shows better agreement with the experimental observations for [C_2_mim][BF_4_] and [C_2_mim][TFO] ILs. These observations suggest that further refinement of the cp2k-charge force field is necessary to match ionic conductivity predictions with experimental values.

#### Radial Distribution Functions

The resulting radial distribution functions (RDFs) between the center-of-mass (COM) of the cation and anion for each of the ILs are shown in Figures S11–S14 (see Supporting Information, Section S2.4). As expected, ionic interactions in these systems induce periodicity in RDFs, reflecting alternating layers of ions with opposite charges. The well-defined first peaks in the cation–anion COM RDFs, compared to the broader peaks for anion–anion and cation–cation contacts, highlight the dominant role of cation–anion interactions in forming discrete ionic shells. Additionally, the varying radius of the first solvation shell underscores the influence of anion size on the overall IL structure. Notably, the positions of the first maxima and the general behavior of the cation–anion COM RDFs align with existing data for similar ILs.^28^ Among the four anions considered, Figures S11–S14 reveal a trend in interaction strength with the [C_2_mim]^+^ cation. The value of the first peak indicates a progressively weaker interaction in the order: [BF_4_]^−^ > [TFO]^−^ > [NTF_2_]^−^ > [DCA]^−^.

The COM RDFs, for [C_2_mim][BF_4_] (Figure S11), predicted by the VSIL force field and those obtained with cp2k-charges are very similar in all respects, with a slightly narrower first solvation peak for the cation–cation RDF with cp2k-charges, which can be rationalized on the basis that the overall charge for this IL is nearly identical for both the force fields. In the case of [C_2_mim][DCA] (Figure S12), the [DCA]^−^ – [DCA]^−^ RDF computed using cp2k-charges exhibits a distinct prepeak followed by a strong peak within the first solvation shell. On the other hand, the [DCA]^−^ – [DCA]^−^ RDF computed using VSIL displays a shoulder followed by a strong peak. The primary reason for the difference in the prepeak intensity stems from reduced electrostatic repulsion between the anions, as the net charge is almost 75% in the cp2k-derived charges versus that in the VSIL force field. The cation–anion and cation–cation RDFs for [C_2_mim][TFO] are identical when compared to the two force fields. The anion–anion RDF, however, shows a subtle difference in the first solvation shell (Figure S13). The cp2k-charges yield a split in the first peak while it is flatter for the VSIL force field, suggesting a slightly more diffuse first solvation shell for the anion. cp2k-charges show a flatter peak for the [C_2_mim]^+^ – [NTF_2_]^−^ pair, as shown in Figure S14, suggesting a stronger and more localized interaction compared to the shoulder observed in the VSIL model. This can be due to the overlay of all possible ion–ion arrangements, especially characteristic of bulky multiatomic [NTF_2_]^−^ ions. These observations suggest that, in general, ionic arrangements are not radically influenced by the variation in the net charge on the cation and anion; however, subtle rearrangement of the anion–anion can be expected, due to the effect of electronic polarizability as seen in several ab initio molecular dynamics simulations.^72,73^

**Figure 5:**
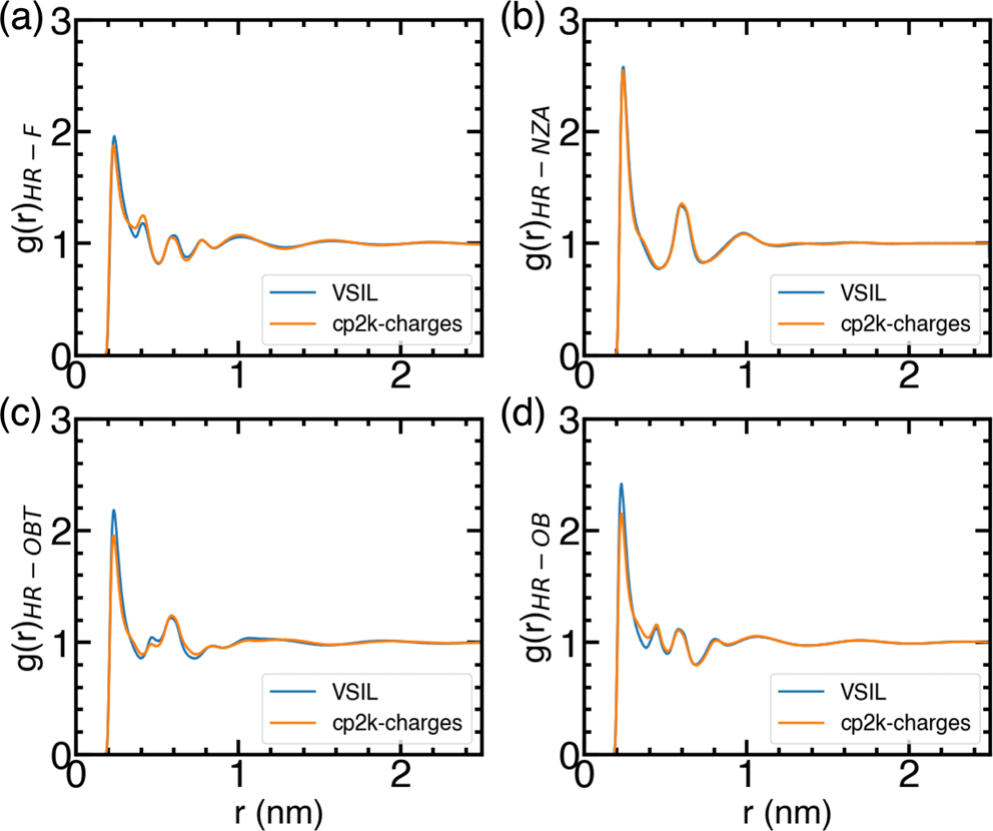
Atom–Atom RDFs in pure ILs representing hydrogen bonding: (a) [C_2_mim][BF_4_], (b) [C_2_mim][DCA], (c) [C_2_mim][NTF_2_], and (d) [C_2_mim][TFO].

As the hydrogen bonding interactions in imidazolium-based ILs are responsible for many physicochemical properties, the RDFs between the HR atom of the imidazolium ring and electronegative atom in the anion (HR-F, HR-NZA, HR-OBT, and HR-OB in [BF_4_]^−^, [DCA]^−^, [NTF_2_]^−^, and [TFO]^−^, respectively (see Figure 1 for the nomenclature) are investigated (see Figure 5). Despite almost identical net charges on ions in [C_2_mim][BF_4_] for both cp2k-charges and VSIL force fields, partial atomic charges are more negative (F atoms) or positive (HR atoms) for cp2k-charges than VSIL (see Table S1), leading to a shorter distance between HR and F atoms in [C_2_mim][BF_4_] by ∼0.1 Å. This enhances the hydrogen bonding interaction between [C_2_mim]^+^ and [BF_4_]^−^, lowering the self-diffusion coefficients (Table S2) and ionic conductivity (Figure 4). Interestingly, the peak height corresponding to the first maximum in the HR-F RDF is predicted to be slightly lower in cp2k-charges than that in the VSIL force field because of steric repulsion between [BF_4_]^−^.

Although the net charges on ions are significantly different in [C_2_mim][DCA], for both cp2k-charges and VSIL, the distance between HR and NZA atoms are found to be similar, resulting in similar hydrogen bonding interactions in both cp2k-charges and VSIL. This result is somewhat surprising given that the partial atomic charges on NZA atoms in [C_2_mim][DCA] are lower for cp2k-charges than VSIL, while charges on HR atoms are comparable for both cp2k-charges than VSIL. Despite similar hydrogen bonding interactions, the self-diffusion coefficients and the ionic conductivity are markedly higher for cp2k-charges. This observation suggests that the overall reduction in the electrostatic interactions in cp2k-charges probably plays a more important role than the hydrogen bonding interactions in determining transport properties.

In the case of [C_2_mim][NTF_2_] and [C_2_mim][TFO], the peak heights are lower when calculated using cp2k-charges compared to the VSIL force field. However, the peak distance, which represents the average distance between HR and OBT atoms in [C_2_mim][NTF_2_] and HR and OB atoms in [C_2_mim][TFO], is similar for both cp2k-charges and the VSIL force field. This suggests comparable hydrogen bonding interactions between both cp2k-charges and the VSIL. Nevertheless, the ionic conductivities are found to be higher when calculated using cp2k-charges compared to that with the VSIL force field (Figure 4). The higher ionic conductivity achieved from cp2k-charges is attributed to the weaker electrostatic interactions that facilitate faster ion diffusion, ultimately leading to higher ionic conductivity.

The suitability of the chosen OPLS-based VSIL force field and cp2k-charges for studying pure ILs was assessed by comparing the simulated density, structural properties, self-diffusion coefficient, and ionic conductivity to both experimental data and established computational results. Remarkably, excellent agreement was observed, indicating the validity of our chosen methodology. Consequently, the selected OPLS-based VSIL force field and cp2k-charges prove to be suitable for our further study. Thus, the above properties for the binary IL mixtures will be predicted using three sets of overall charges on the cation and anion: (a) VSIL, (b) cp2k-charges obtained directly from condensed phase DFT calculations for trajectories with a given mole fraction of anions, and (c) the interpolated-charges also referred to as interpolated-charges in which cation charges are linearly interpolated based on the charges obtained for pure ILs while the charge on the anion obtained for pure IL was retained for mixtures.

### Properties of IL Mixtures

#### Density

The liquid-phase density of four binary IL mixtures was simulated using cp2k-charges, interpolated-charges, and VSIL force fields at 298 K and 1 bar. The results for the density of these mixtures are tabulated in Tables S5–S8 and presented in Figure S15 as a function of [BF_4_]^−^ or [TFO]^−^ mole fractions. Density variation for [C_2_mim][BF_4_][DCA] is found to be linear across the entire composition range. As opposed to this behavior, a nonlinear trend in the density variation can be detected for the other three IL mixtures. The density trends predicted by all of the force field models are similar. Comparing predicted values of densities across different force fields, it can be seen that the densities obtained by VSIL force fields are consistently higher for [C_2_mim][BF_4_][DCA], [C_2_mim][BF_4_][TFO], and [C_2_mim][TFO][NTF_2_] mixtures, suggesting a more dense packing of ions for these systems for the VSIL force field. However, for [C_2_mim][BF_4_][NTF_2_], the density was found to be nearly identical for the three sets of force fields at a given composition, implying that the effects of packing are represented similarly across the force fields. Furthermore, the predicted densities of [C_2_mim][BF_4_][DCA] and [C_2_mim][BF_4_][NTF_2_] mixtures are in good agreement with experimentally measured values reported by Stoppa et al.^20^ and Trenzado et al.,^74^ respectively (Figure S15). Although experimental values for the other two mixtures are not available, we suspect that the density trends and values would be in good agreement with the predicted densities, as the predicted densities of pure counterparts closely match the experimental values (Table 1). Moreover, a linear trend in density predictions was also observed for the IL mixtures of [C_4_mim]Cl methyl sulfate [MeSO_4_] due to similar hydrogen bonding ability of the anions, while a nonlinear density variation was noted for [C_4_mim]Cl[NTF_2_] mixtures for which the hydrogen bonding ability of the anions differed considerably.^13^ A large difference in the pure IL molar volume is also a contributing factor.

#### Self-Diffusion Coefficient

Self-diffusion coefficients for binary IL mixtures were computed using cp2k-charges and interpolated-charges and are tabulated in Tables S9–S12, along with those determined for the VSIL force field. Corresponding figures are provided in Figure S18, Supporting Information, and Section S3.4. As it is evident, the trends and numerical values for two different methods of assigning charges (cp2k-charges and interpolated-charges) are very similar. Therefore, the following discussion focuses on interpolated-charges, henceforth referred to as the interpolated-charge force field.

**Figure 6:**
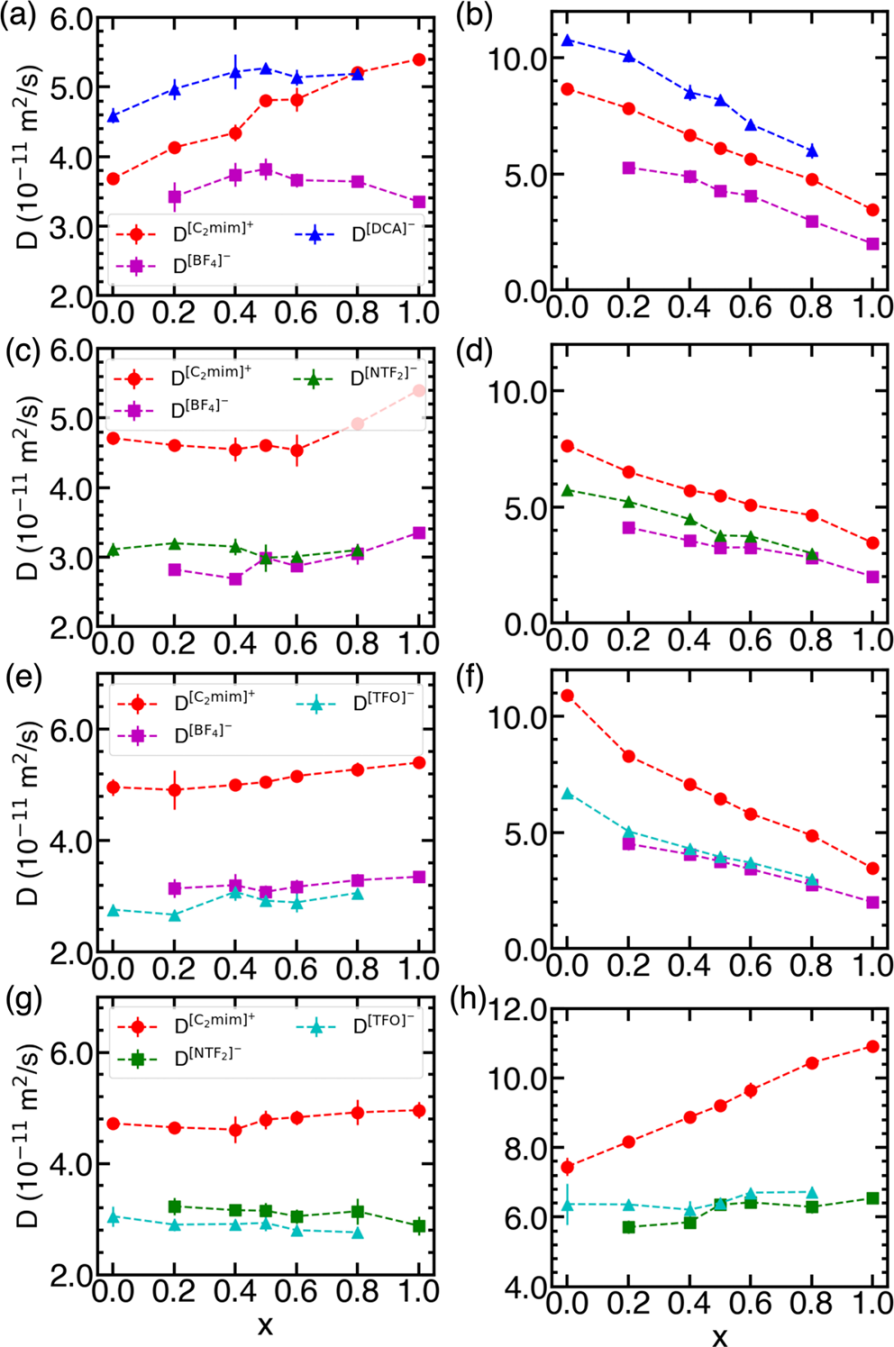
Self-diffusion coefficients of ions in (a, b) [C_2_mim][BF_4_][DCA], (c, d) [C_2_mim][BF_4_][NTF_2_], (e, f) [C_2_mim][BF_4_][TFO], and (g, h) [C_2_mim][TFO][NTF_2_] computed using VSIL (left pane) and interpolated-charges (right pane). The dashed lines act only as a visual guide.

Figure 6 collects the self-diffusion coefficients of the cation and anion for the IL mixtures studied in this work. It can be deduced that the self-diffusion coefficients of ions are comparable in magnitude to those observed for pure ILs, and they are bracketed by the self-diffusion coefficients of ions in the corresponding pure ILs. The extent of variation and trend in the self-diffusion coefficient is markedly different for the interpolated-charges from the VSIL force field. In particular, across all of the mixtures, the cation/anion self-diffusion coefficients decrease (increase) with an increase in the [BF_4_]^−^ ([TFO]^−^) concentrations for interpolated-charges. In [C_2_mim][BF_4_][DCA] mixtures, [DCA]^−^ diffuses the fastest, followed by [C_2_mim]^+^, and the slowest diffusing species is [BF_4_]^−^. For all other mixtures, the self-diffusion coefficients of the two anions are nearly identical, and the cation is the fastest diffusing species, which is consistent with the observations reported by Kapoor and Shah.^11,13,31^

Interpretation of VSIL predictions is rather complex, as the trends in the self-diffusion coefficient appear to be dependent on the type of IL mixture. For example, in the case of [C_2_mim][BF_4_][DCA] mixtures, the self-diffusion coefficient of the cation and [DCA]^−^ increases with respect to the [BF_4_]^−^ concentration, while the self-diffusion coefficient of [BF_4_]^−^ passes through a maximum at equimolar concentration. Also, the self-diffusion coefficient of [DCA]^−^ is almost identical to that for [C_2_mim]^+^ at *x* = 0.8, implying that the cation and [DCA]^−^ diffuse together. On the other hand, the self-diffusion coefficient of the cation shows minimal variation up to equimolar concentrations in [C_2_mim][BF_4_][NTF_2_] mixtures and increases in the [BF_4_]^−^-rich mixtures. The variation in the self-diffusion coefficients of anions in mixtures of [C_2_mim][BF_4_][TFO] and [C_2_mim][TFO][NTF_2_] is minimal.

#### Ionic Conductivity

The ionic conductivity of binary IL mixtures was calculated using the NE (eq 4) and Einstein formalism (eq 5). Similar to self-diffusion coefficients, the ionic conductivities for binary IL mixtures were determined using two distinct sets of total charges for the cation and anion. Ionic conductivities calculated using these two types of charges, along with those determined for the VSIL force field, are presented in Tables S13–S16 and Figure S19 (see Supporting Information, Section S3.5). As expected, the NE ionic conductivity consistently exceeds the Einstein conductivity due to the neglect of ion–ion correlations. The ionic conductivities observed are comparable in magnitude to those obtained with cp2k and interpolated-charge force fields, showing a consistent trend across both cp2k-charges and interpolated-charges (see Figure S19). While minor variations in conductivity appear for some IL mixtures at specific concentrations, these differences are minimal and do not substantially affect the overall trends.

**Figure 7:**
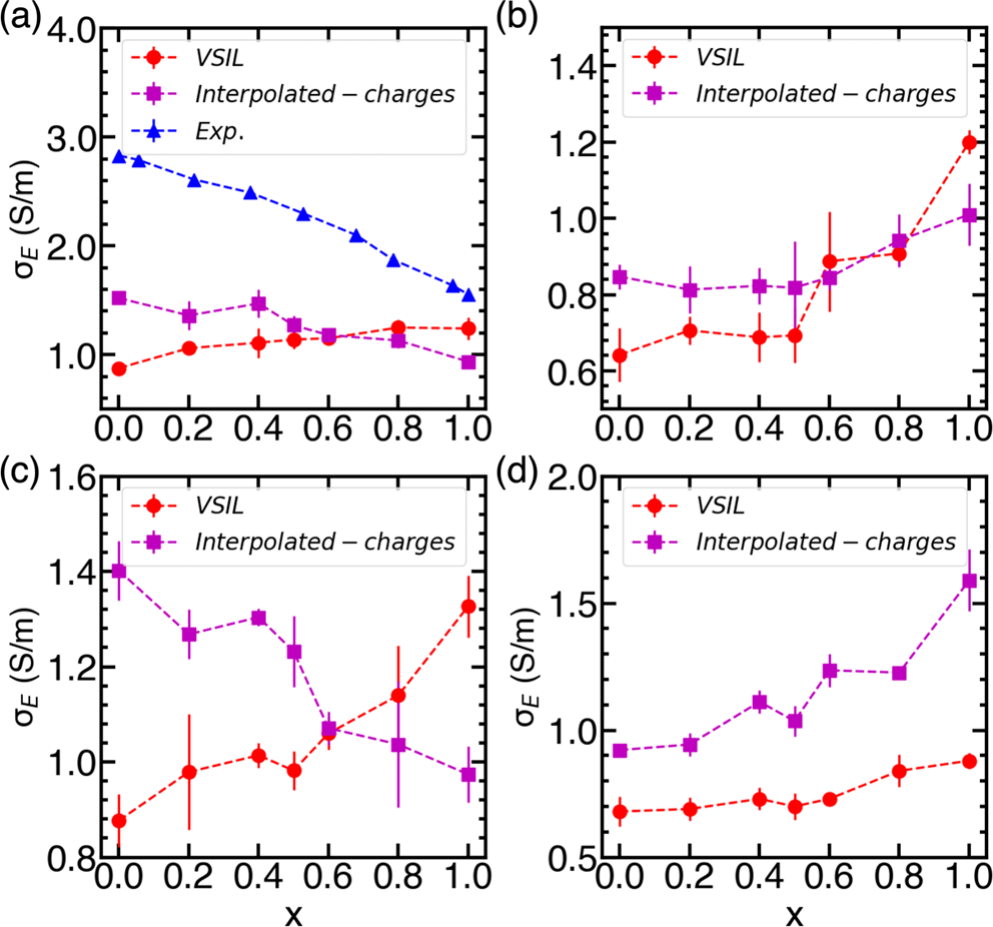
Einstein conductivity of (a) [C_2_mim][BF_4_][DCA], (b) [C_2_mim][BF_4_][NTF_2_], (c) [C_2_mim][BF_4_][TFO], and (d) [C_2_mim][TFO][NTF_2_] computed using VSIL and interpolated-charges. The dashed lines only act as a visual guide.

Figure 7 illustrates the Einstein conductivities, calculated using interpolated-charge and VSIL force fields. Although the ionic conductivity values of the binary IL mixtures, similar to their self-diffusion coefficients, fall within the ionic conductivity range of pure ILs, the degree of variation and the trends in ionic conductivity differ markedly between predictions from the interpolated-charges and VSIL force fields. In the case of [C_2_mim][BF_4_][DCA], the Einstein conductivity for binary IL mixture decreases with the concentration of [BF_4_]^−^ in the case of interpolated-charges consistent with the experimental observations.^20^ However, the predicted values are lower than the experimental values at all concentrations. On the other hand, the ionic conductivity computed using the VSIL force field shows an opposite trend. Similarly, the trends in the ionic conductivity as a function of [BF_4_]^−^ concentrations are opposite for the two sets of charges for the [C_2_mim][BF_4_][TFO] systems. However, the trend of ionic conductivity is identical for [C_2_mim][BF_4_][NTF_2_] and [C_2_mim][TFO][NTF_2_] mixtures, irrespective of the force field employed.

The predicted Einstein conductivities align well with experimental values for [C_2_mim][BF_4_][DCA] mixtures using the VSIL force field in [BF_4_]^−^-rich and interpolated-charges in [DCA]^−^-rich mixtures, which can be understood on the basis that the VSIL force field is more accurate in predicting the ionic conductivity for pure [C_2_mim][BF_4_] while the interpolated-charge force field yields better accuracy for [C_2_mim][DCA]. Although experimental data for other mixtures are unavailable, comparisons with predicted conductivities and existing experimental data for pure ILs allow further interpretation. For example, it can be anticipated that, for mixtures of [C_2_mim][BF_4_][NTF_2_] or [C_2_mim][DCA][TFO], the VSIL force field outperforms interpolated-charges in estimating ionic conductivity in the [BF_4_]^−^-rich or [TFO]^−^-rich mixtures, while interpolated-charges may yield more accurate results in [NTF_2_]^−^-rich or [DCA]^−^-rich mixtures, respectively. In particular, for [C_2_mim][BF_4_][TFO], VSIL could provide more reliable conductivity trends and values. On the other hand, in [C_2_mim][DCA][NTF_2_] mixtures, interpolated-charges may offer improved accuracy for conductivity trends as well as values. Furthermore, analogous to the observations for [C_2_mim][BF_4_][DCA] mixtures, VSIL may deliver better accuracy for [TFO]^−^-rich mixture, whereas interpolated-charges are expected to yield more precise conductivity predictions for [NTF_2_]^−^-rich [C_2_mim][TFO][NTF_2_] mixtures.

#### Radial Distribution Functions

**Figure 8:**
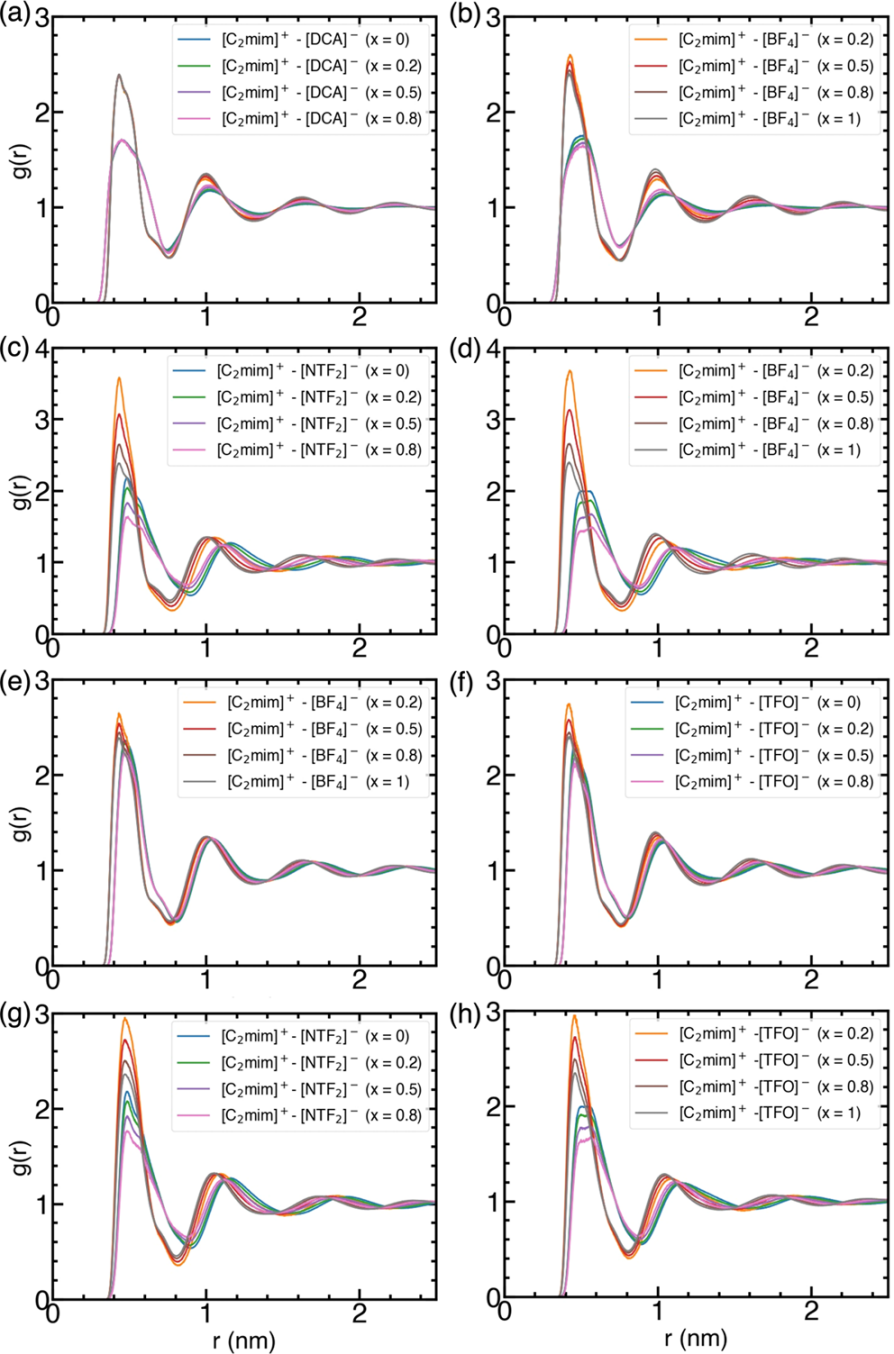
COM-COM RDFs for cation–anion computed using VSIL (left pane) and interpolated-charges (right pane). (a, b) [C_2_mim][BF_4_][DCA], (c, d) [C_2_mim][BF_4_][NTF_2_], (e, f) [C_2_mim][BF_4_][TFO], and (g, h) [C_2_mim][TFO][NTF_2_] mixtures with varying concentrations.

Figure 8 represents RDFs between the COM of the cation and anion for each IL mixture with varying concentrations of [BF_4_]^−^ or [TFO]^−^. As observed for the pure IL, the charge ordering is preserved in IL mixtures as well. The positions of the first peak do not vary significantly as a function of anion composition for all of the studied mixtures and are similar to those found for respective pure IL counterparts. Peak positions for [C_2_mim]^+^ – [BF_4_]^−^ RDFs are predicted to be nearly identical with both sets of charges. A similar observation holds for the [C_2_mim]^+^ – [TFO]^−^ RDFs. Contrary to this observation, interpolated-charges lead to peak positions at a greater distance for [C_2_mim]^+^ – [DCA]^−^ RDFs and [C_2_mim]^+^ – [NTF_2_]^−^ RDFs in comparison to those for the VSIL force field. These differences highlight the contribution of electrostatic interactions governed by partial charges and sizes of the anions.

Delving deeper into the cation–anion RDFs, different trends in the height of the first peak can be discerned based on the type of charges employed. In the case of [C_2_mim][BF_4_][DCA] mixtures, the peak height remains roughly constant for both [C_2_mim]^+^ – [BF_4_]^−^ and [C_2_mim]^+^ – [DCA]^−^ RDFs when the VSIL force field is implemented, implying that there is no influence of [BF_4_]^−^ on the localization of [DCA]^−^ and vice versa. On the other hand, the peak height of [C_2_mim]^+^ – [BF_4_]^−^ decreases with [BF_4_]^−^ concentration, which indicates that [BF_4_]^−^ preferentially interacts with [C_2_mim]^+^ at the lowest [BF_4_]^−^ concentration. With an increase in the concentrations, [BF_4_]^−^ begins to occupy additional binding sites, which lead to a drop in the intensity. A concomitant decrease in the peak height of [DCA]^−^ points to the displacement of [DCA]^−^ from its favorable binding site as the [BF_4_]^−^ concentration increases.

Furthermore, RDFs between the HR atom in the ring of [C_2_mim]^+^ and F, NZA, OBT, and OB atoms in anions ([BF_4_]^−^, [DCA]^−^, [NTF_2_]^−^, and [TFO]^−^, respectively) are obtained to investigate how hydrogen bonding interactions are impacted in IL mixtures (see Figures S22–S25). The positions of the first peak in the RDFs exhibit minimal variation with changes in the anion concentration across the studied mixtures, generally aligning with trends noted for the COM-COM RDF positions. A notable exception, however, occurs with [C_2_mim][BF_4_][DCA] mixtures in the absence of the [DCA]^−^ anion. In this IL mixture, as the concentration of [BF_4_]^−^ anions increases, the height of the initial peak in both the HR-NZA and HR-F RDFs decreases. A significant difference in peak height in the HR-F RDFs is observed when comparing interpolated-charge to the VSIL force field, highlighting the considerable effect of the force field choice on the representation of hydrogen bonding strength. On the other hand, the difference in peak height for the HR-NZA RDFs remains insignificant, indicating minimal sensitivity to the selected force field in hydrogen bonding strength. Furthermore, the HR-F RDF peak position for interpolated-charge models is 0.08 Å lower compared to that observed with the VSIL force field at *x* = 1.0, indicating a stronger hydrogen bonding interaction in pure [C_2_mim][BF_4_]. Additionally, a sharp decline in the HR-F RDF peak is observed at *x* = 1.0 when an interpolated-charge force field was employed.

Interestingly, [BF_4_]^−^ tends to stay closer to the [C_2_mim]^+^ cation as the size of the second anion in the mixture increases. This behavior is correlated with differences in molar volume: as the differences in molar volume between the two ILs become larger, the [BF_4_]^−^ anion moves closer to the [C_2_mim]^+^ cation. This effect is observable in the shift of the HR-F RDF peak position, which reflects a more compact local structure between [BF_4_]^−^ and [C_2_mim]^+^. The preferential association of [BF_4_]^−^ with [C_2_mim]^+^ likely results from the spatial effects introduced by the larger coanion. Similar observations were also made in various IL mixtures reported through molecular dynamics simulations ([C_4_mim][PF_6_][Cl] and [C_4_mim][PF_6_][BF_4_]) and ab initio molecular dynamics simulations (binary mixture of [C_2_mim][Cl] and [C_2_mim][SCN]) involving smaller anions that exhibited a stronger tendency to displace larger anions. This resulted in the formation of hydrogen bonds with the more acidic H atom of the imidazolium cation ring (e.g., [C_2_mim]^−^ or [C_4_mim]^+^).^75−77^

#### Coordination Numbers

**Figure 9:**
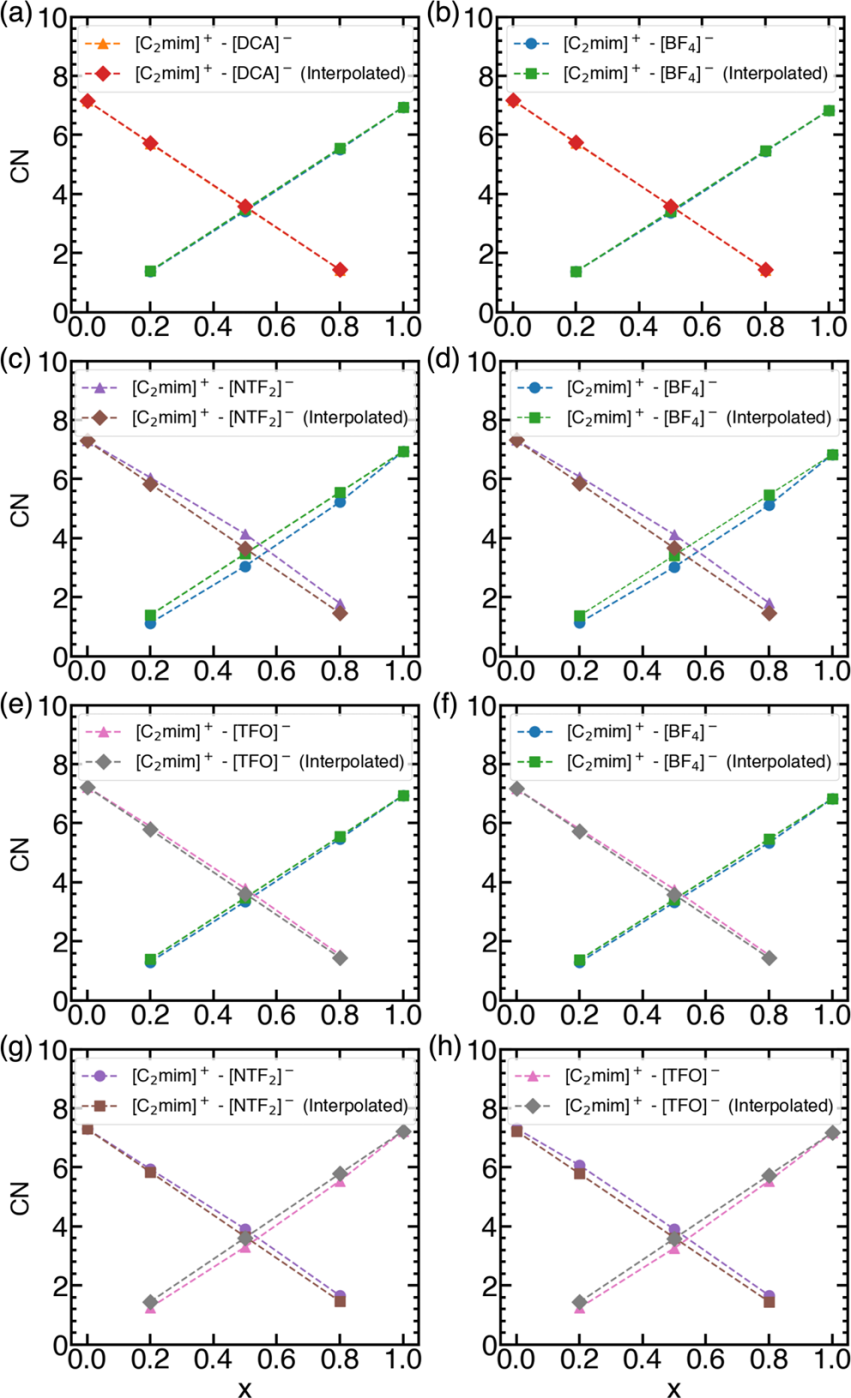
First shell coordination numbers as a function of composition for (a, b) [C_2_mim][BF_4_][DCA], (c, d) [C_2_mim][BF_4_][NTF_2_], (e, f) [C_2_mim][BF_4_][TFO], and (g, h) [C_2_mim][TFO][NTF_2_] mixture using VSIL (left pane) and interpolated-charges (right pane). Interpolated first shell coordination numbers are obtained by multiplying the coordination numbers in pure ILs by their concentrations.

In binary IL mixtures, the coordination number (CN) of the ions surrounding a reference ion can be significantly altered by varying the mole fraction of one anion. This arises from an interplay of factors, including size differences, charge distribution and polarizability, specific interactions, and changes in local density. Understanding this relationship between mole fraction and coordination number is crucial for elucidating the structure and behavior of this mixture. CNs of the anions COM relative to the [C_2_mim]^+^ COM as a function of radius are shown in Figures S30 and S31 (see Supporting Information, Section S3.7). Looking at the CNs in the first coordination shell, the CNs of the second anion decrease with an increase in the concentration of the first anion. As the composition of the first anion increases, the competition for coordination sites around the reference ion ([C_2_mim]^+^) also increases (Figure 9). This can lead to displacement of the second anion and a change in the overall coordination number. The linear variation of the number of anions in the first coordination shell of the cation probably explains why the charge on the cation varies linearly with the concentration. Upon further investigation into CNs, it can be observed that there is a linear relationship between CN and concentration for [C_2_mim][BF_4_][DCA] Figure 9(a,b). Hence, the ratio of CN of ions to that in pure ILs is expected to be proportional to its concentration, which indicates a simple exchange of ions in the first coordination shell of the cation with concentration.

In Figure 9(c,d), the variation in the CN of [BF_4_]^−^ and [NTF_2_]^−^ ions surrounding [C_2_mim]^+^ is depicted as a function of [BF_4_]^−^ concentration. Similar to [C_2_mim][BF_4_][DCA], CNs of [BF_4_]^−^ increases while CNs of [NTF_2_]^−^ decreases. However, variations in CNs are not linear, as seen for [C_2_mim][BF_4_][DCA]. Moreover, similar variations in CNs are seen in other IL mixtures (Figure 9(e–h)). Similar observations were made for various IL mixtures, such as [C_4_mim][PF_6_][BF_4_] and [C_4_mim][PF_6_][Cl], where both anions are coordinated to the H_2_ site to an extent that is nearly proportional to their mole fractions in the [C_4_mim][PF_6_][BF_4_] mixture.^75^

## Conclusions

This study investigated binary mixtures of ILs sharing a common [C_2_mim]^+^ cation using MD simulations with the objective of predicting the ionic conductivity of these mixtures using a force field with fixed charge scaling of 0.8 (VSIL) and a model in which the variable charge scaling was employed, mimicking polarization effects obtained via periodic DFT calculations. Our results showed that the charge scaling for a given IL depended on the type of the anion and varied between 0.65 and 0.81, underscoring the anion-dependent polarization in ILs. Consistent with the magnitude of charge scaling with respect to that used in VSIL, densities were predicted to be higher when the charge scaling was higher and vice versa. The difference in densities obtained with two force fields, however, was less than a few percentages, suggesting that the different distribution of charges does not significantly influence the density of ILs. On the other hand, transport properties such as self-diffusion coefficients and ionic conductivities are markedly dependent on the magnitude of charge scaling with deviation from the VSIL force field as large as 100% observed. From the calculations, an acceleration in system dynamics could be inferred with a lowering of charge scaling. Our results highlight that the physical origin of the differing transport properties is dependent on the type of IL. In [C_2_mim][BF_4_], stronger hydrogen bonding in cp2k-charges reduces conductivity, whereas weaker electrostatic interactions in cp2k-charges for [C_2_mim][DCA], [NTF_2_], and [TFO] increase ion diffusion and conductivity. While hydrogen bonding distances are often comparable between cp2k-charges and VSIL, the weaker interactions in cp2k-charges consistently lead to higher ionic conductivities.

Furthermore, charges were derived for IL mixtures as a function of anion concentrations, which indicated that the net charge on the anion remained nearly identical to that found in pure IL. However, the cation charge was observed to vary with the composition. In fact, the net charge on the cation could be approximated as a mole fraction-weighted average of the pure IL cation charges. Using the new sets of charges, compositional dependence of various physicochemical properties, such as density, excess molar volume, self-diffusion coefficients, ionic conductivities, and structural properties, was probed. Densities obtained using the VSIL force field consistently show higher values for [C_2_mim][BF_4_][DCA], [C_2_mim][BF_4_][TFO][C_2_mim], and [TFO][NTF_2_] mixtures in comparison to those obtained with new sets of charges. This suggests a more dense packing of ions for these systems when using the VSIL force field. However, for [C_2_mim][BF_4_][NTF_2_], the density was found to be nearly identical for the three sets of force fields at a given concentration. This implies that the effects of packing are represented similarly across the force fields. Additionally, the predicted densities of the [C_2_mim][BF_4_][DCA] and [C_2_mim][BF_4_][NTF_2_] mixtures were in good agreement with experimentally measured values. Furthermore, the excess molar volumes for all of the studied systems were less than 1 cc/mol, indicating that the mixing process is ideal and that the packing in pure ILs is not significantly perturbed.

The self-diffusion coefficients of binary IL mixtures were observed to be between those of their pure counterparts. The self-diffusion coefficients of cations were higher than those of anions except for the mixture containing [DCA]^−^ anions. Similarly, the ionic conductivity values of binary IL mixtures fall within the ionic conductivity range of pure ILs. Consequently, the degree of variation and the trends in ionic conductivity differ significantly between predictions from interpolated-charges and VSIL force fields. The radial distribution functions for the cation–anion and the HR-M (M: F, NZA, OBT, and OB) pair indicate that the smaller anion tends to form hydrogen bonds with the most acidic proton (HR). The probability of this event increases with decreasing concentrations of the smaller anion. Moreover, the dependence of these quantities on anion concentration is more pronounced for a larger difference in anion size.

Both cp2k-charges and interpolated-charge models consistently yielded similar values for the properties investigated here. Moreover, they successfully reproduced the experimentally observed compositional dependence of densities for [C_2_mim][BF_4_][DCA] and [C_2_mim][BF_4_][NTF_2_] mixtures along with Einstein conductivities for [C_2_mim][BF_4_][DCA] mixtures. This success suggests that calculating partial charges for pure ILs using condensed-phase DFT calculations, followed by interpolation for mixtures, provides a reliable approach for predicting ionic conductivity trends in binary IL mixtures. This strategy offers a computationally efficient alternative to full charge calculations for mixtures while maintaining accuracy.

Our results demonstrate that the conductivity of IL mixtures obtained using partial atomic charges derived from condensed phase DFT calculations serves as a good initial approximation. Although the new set of charges seems to improve the ionic conductivity predictions for certain mixtures, the force field is not optimized to reproduce other properties, such as the heat of vaporization, and the performance of the force field in predicting viscosity remains to be evaluated. Other works focus on the optimization of Lennard-Jones parameters to achieve improved accuracy of predictions for ionic conductivity for [C_2_mim][BF_4_] and [C_2_mim][TFO] ionic liquids, as proposed in several recent publications.^60,78^ In addition, the work is currently underway on investigating the effect of polarization in IL mixtures containing two cations and a common anion to assess if the linearity of net charges with the mole fraction holds for the anion in such mixtures. We also encourage researchers to measure the physicochemical properties of IL–IL mixtures for benchmarking force fields specifically derived for IL mixtures.

## Data Availability

Raw data and simulation files are hosted on https://github.com/ShahResearchGroup/JPCB_IL_Mixtures_2025.

## Supporting Information Available

Method, protocol, partial atomic charges, density of pure ILs; self-diffusion coefficients of pure ILs; Nernst–Einstein and Einstein conductivity of pure ILs; radial distribution function of pure ILs; density of IL mixtures; molar volume of IL mixtures; excess molar volume of IL mixtures; self-diffusion coefficients of IL mixtures; Nernst–Einstein and Einstein conductivity of IL mixtures; radial distribution functions of IL mixtures; and coordination numbers of IL mixtures; spatial distribution function of IL mixtures (PDF)
