# Supplementary material for: Predicting Ionic Conductivity of Imidazolium-Based Ionic Liquid Mixtures Using Quantum-Mechanically Derived Partial Charges in the Condensed Phase
Source: J Phys Chem B. 2025 Feb 21;129(9):2546–59. doi: 10.1021/acs.jpcb.4c08275 (PMC11891901; doi:10.1021/acs.jpcb.4c08275)
Supplement: Supplementary file 1 — jp4c08275_si_001.pdf [file jp4c08275_si_001.pdf]

Supporting Information
Predicting Ionic Conductivity of imidazolium-based Ionic Liquid Mixtures Using Quantum-Mechanically Derived Partial Charges in Condensed Phase

Ashutosh Kumar Verma, Amey S. Thorat, Jindal K. Shah*

School of Chemical Engineering, Oklahoma State University, Stillwater, OK 74078, United States

E-mail: jindal.shah@okstate.edu

Contents

S1 Method and Protocol for Charge Calculation **S3**

S1.1 Method S3

S1.2 Protocol S3

S1.3 Partial Atomic Charges S4

S2 Pure Ionic Liquids **S6**

S2.1 Density S6

S2.2 Self-diffusion Coefficients S6

S2.3 Nernst-Einstein and Einstein Conductivity S15

S2.4 Radial Distribution Functions S16

S3 Binary Ionic Liquid Mixtures **S18**

S3.1 Density S18

S3.2 Molar Volume S20

S3.3 Excess Molar Volume S20

S3.4 Self-Diffusion Coefficients S22

S3.5 Nernst-Einstein and Einstein Ionic Conductivity S24

S3.6 Radial Distribution Functions S26

S3.7 Coordination Numbers S37

S3.8 Spatial Distribution Function S39

S1 Method and Protocol for Charge Calculation

S1.1 Method

Partial charges were computed through quantum mechanical DFT calculations. 10 ion pairs were packed in a cubic box using Packmol.[1] We employed GROMACS to equilibrate the structure using molecular simulation.[2] Subsequently, the equilibrated configuration was utilized for calculating atomic partial charges with cp2k.[3] The crystal structure was simulated in NVT followed by NPT ensemble at 300 K and 1 bar for 35 ns for equilibration. The initial 25 ns of the simulation trajectory were considered equilibration, while snapshots were captured from the last 10 ns of the production run at 1 ns intervals. The derivation of atomic charges involved fitting the long-range electrostatic potential using the DDAP method,[4] averaging over equivalent atoms over 11 snapshots. The generalized gradient approximation (GGA) Perdew-Burke-Ernzerhof (PBE) exchange-correlation functional[5] was used for the electronic energy and the BASIS-MOLOPT basis set[6] to represent the valence electrons in atoms. Norm-conserving Goedecker-Teter-Hutter (GTH) pseudopotentials[7] represented the nuclei and core electrons. A plane wave cut-off of 450 Ry was used. Dispersion interactions were incorporated using Grimme's dispersion D3 model.[8]

S1.2 Protocol

1. Utilize the VSIL force field to conduct MD simulations for generating equilibrated structures.

2. Perform DFT calculations with cp2k to determine partial atomic charges.

3. Employ the partial atomic charges obtained in step 2 and conduct MD simulations to generate equilibrated structures.

4. Perform DFT calculations using cp2k to compute partial atomic charges.

5. Employ the partial atomic charges obtained in step 4 and conduct MD simulations to generate equilibrated structures.

6. Perform DFT calculations using cp2k to compute partial atomic charges.

7. Calculate the charge differences between the partial atomic charges for each atom acquired in steps 6 and 4. If these differences fall within their standard deviations (or $\Delta q \leq 0.03$), no additional calculations are necessary. If the differences exceed their standard deviations (or $\Delta q \geq 0.03$), continue the calculations until the charge differences of each atom fall within their standard deviations (or $\Delta q \leq 0.03$).

S1.3 Partial Atomic Charges

Table S1: Atomic site charges (e) for the anions.

Atoms	cp2k-charges	VSIL charges	Mondal and Balasubramanian[9]
B ($[BF_4]^-$)	1.0080	0.6620	1.0100
F ($[BF_4]^-$)	-0.4530	-0.3655	–0.4500
S ($[CF_3SO_3]^-$)	1.0958	0.9510	1.0900
O ($[CF_3SO_3]^-$)	-0.5533	-0.5245	–0.6000
C ($[CF_3SO_3]^-$)	0.2876	0.2154	0.4400
F ($[CF_3SO_3]^-$)	-0.1448	-0.1310	–0.1700
N ($[NTF_2]^-$)	-0.5888	-0.5280	–0.7400
S ($[NTF_2]^-$)	1.0848	0.8160	1.0900
O ($[NTF_2]^-$)	-0.5125	-0.4240	–0.5450
C ($[NTF_2]^-$)	0.2680	0.2800	0.4450
F ($[NTF_2]^-$)	-0.1308	-0.1280	–0.1550

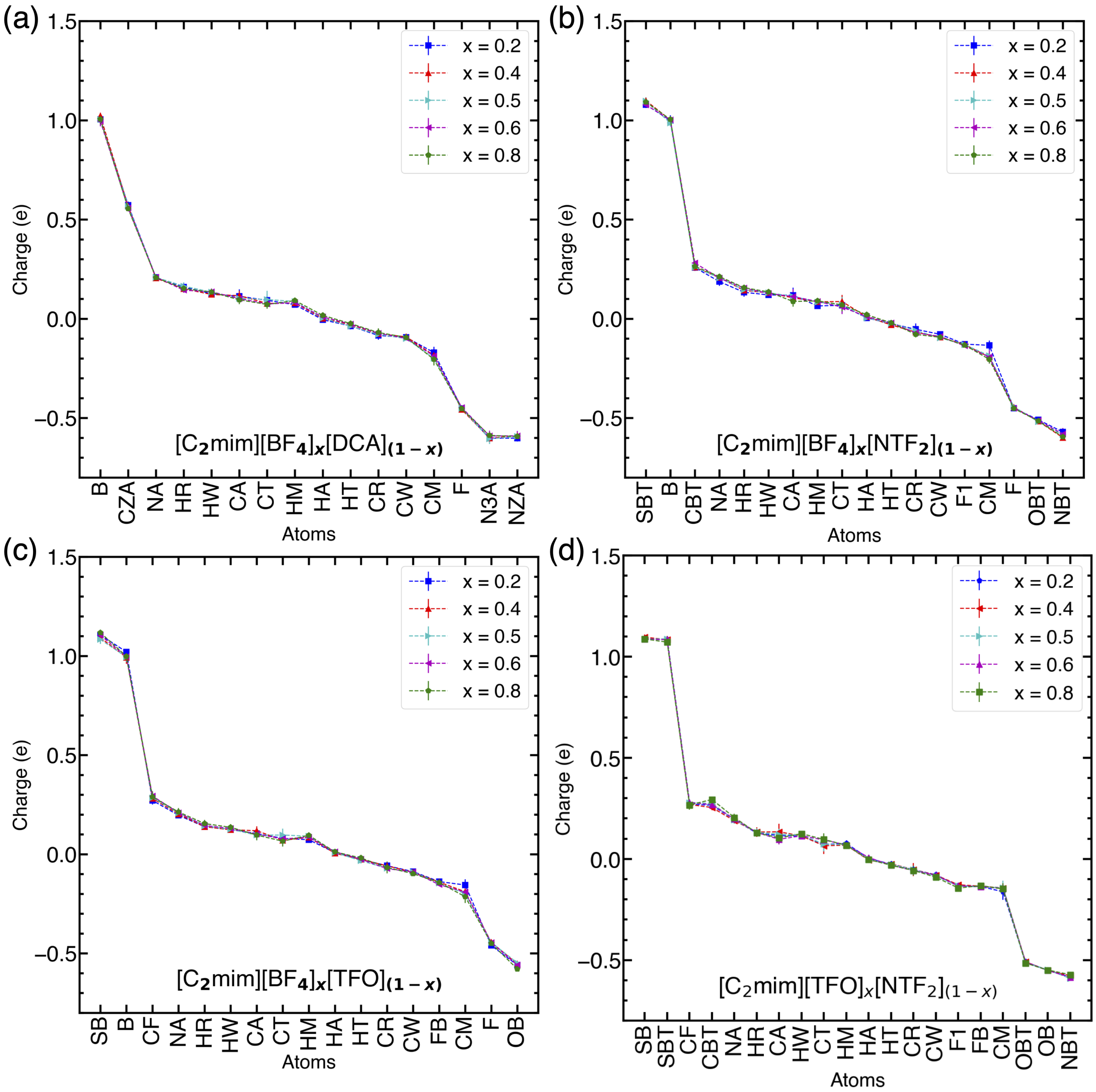

Figure S1: Partial atomic charges of four IL mixtures. The colors blue, red, cyan, magenta, and green represent the composition of $x = 0.2$, $x = 0.4$, $x = 0.5$, $x = 0.6$, $x = 0.8$, respectively. The dashed lines are only to act as a visual guide.

S2 Pure Ionic Liquids

S2.1 Density

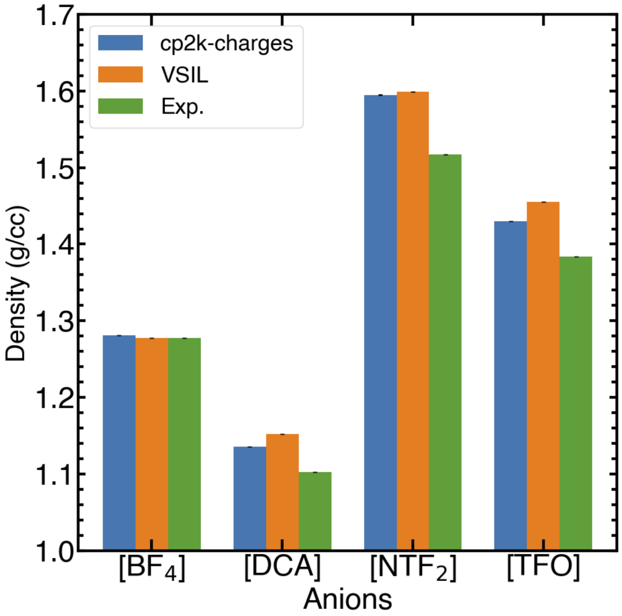

Figure S2: Simulated liquid phase density (g/cc) of pure ILs ([C_2mim][BF_4], [C_2mim][DCA], [C_2mim] [NTF_2] and [C_2mim][TFO]) along with weighted experimental average values at 298 K.

S2.2 Self-diffusion Coefficients

To determine the linear region for the fit, the MSD values were divided into three blocks spanning 0-10 ns, 10-90 ns, and 90-100 ns for the production run. Over these blocks, we computed the non-Gaussian parameter β(t):

$$\beta(t) = \frac{dln(MSD_k(t))}{dln(t)} \tag{S1}$$

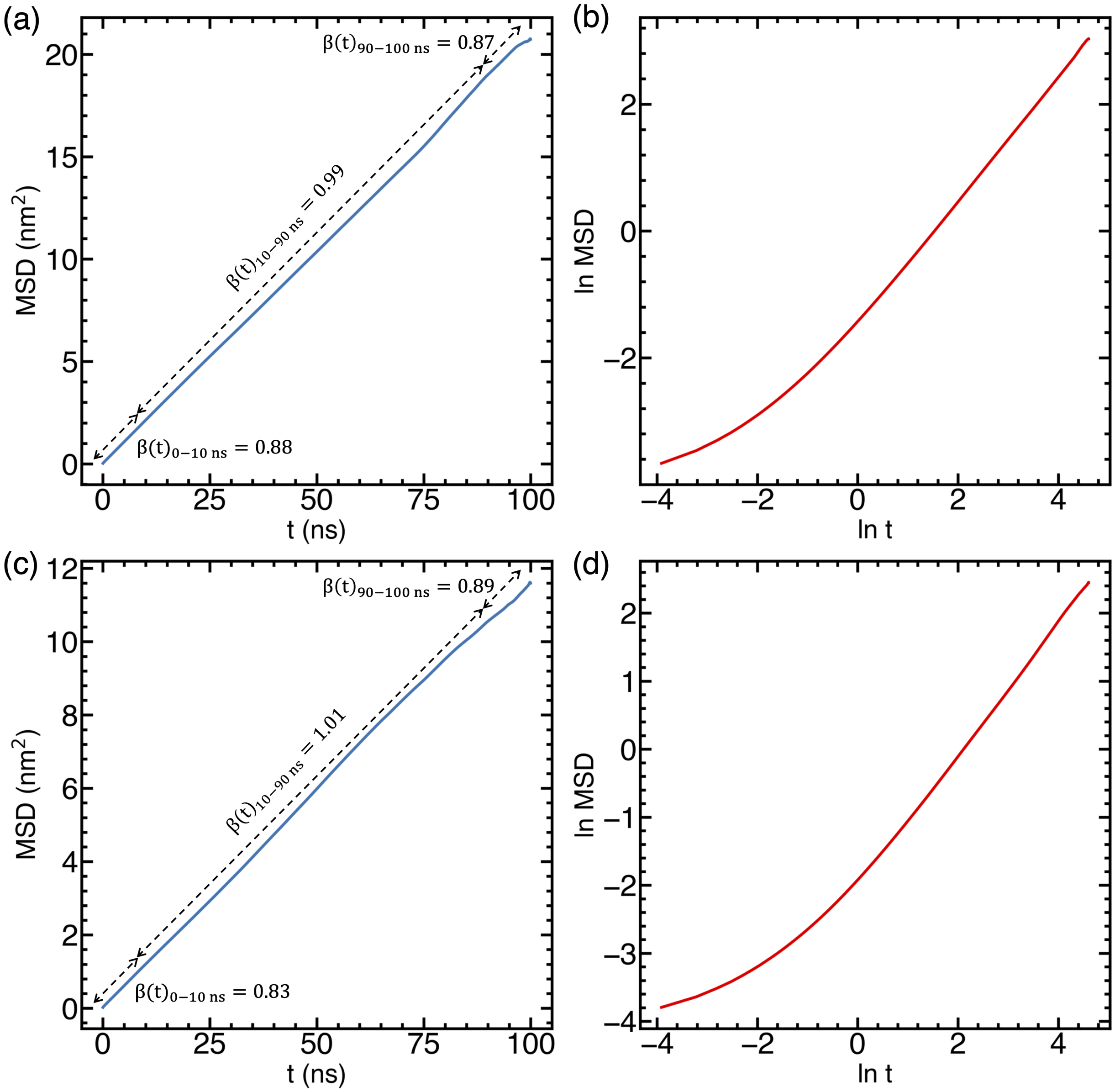

Figure S3: Mean-squared-displacement (MSD) as a function of time and ln MSD vs. ln t for $[C_2mim]^+$ (top pane) and for $[BF_4]^-$ (bottom pane) for pure $[C_2mim][BF_4]$ at 298 K modeled using cp2k-charges.

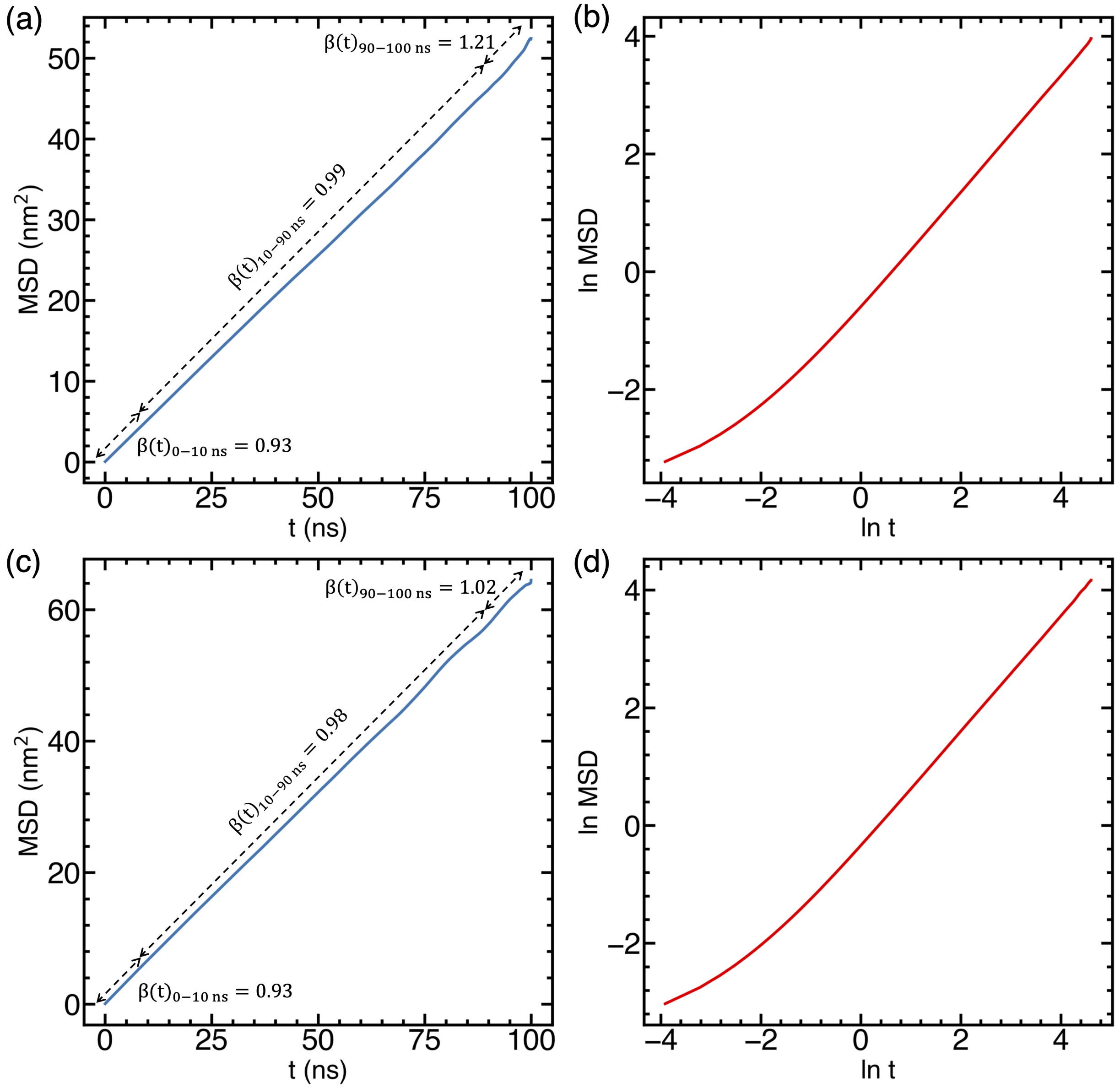

Figure S4: Mean-squared-displacement (MSD) as a function of time and ln MSD vs. ln t for $[C_2mim]^+$ (top pane) and for $[DCA]^-$ (bottom pane) for pure $[C_2mim][DCA]$ at 298 K modeled using cp2k-charges.

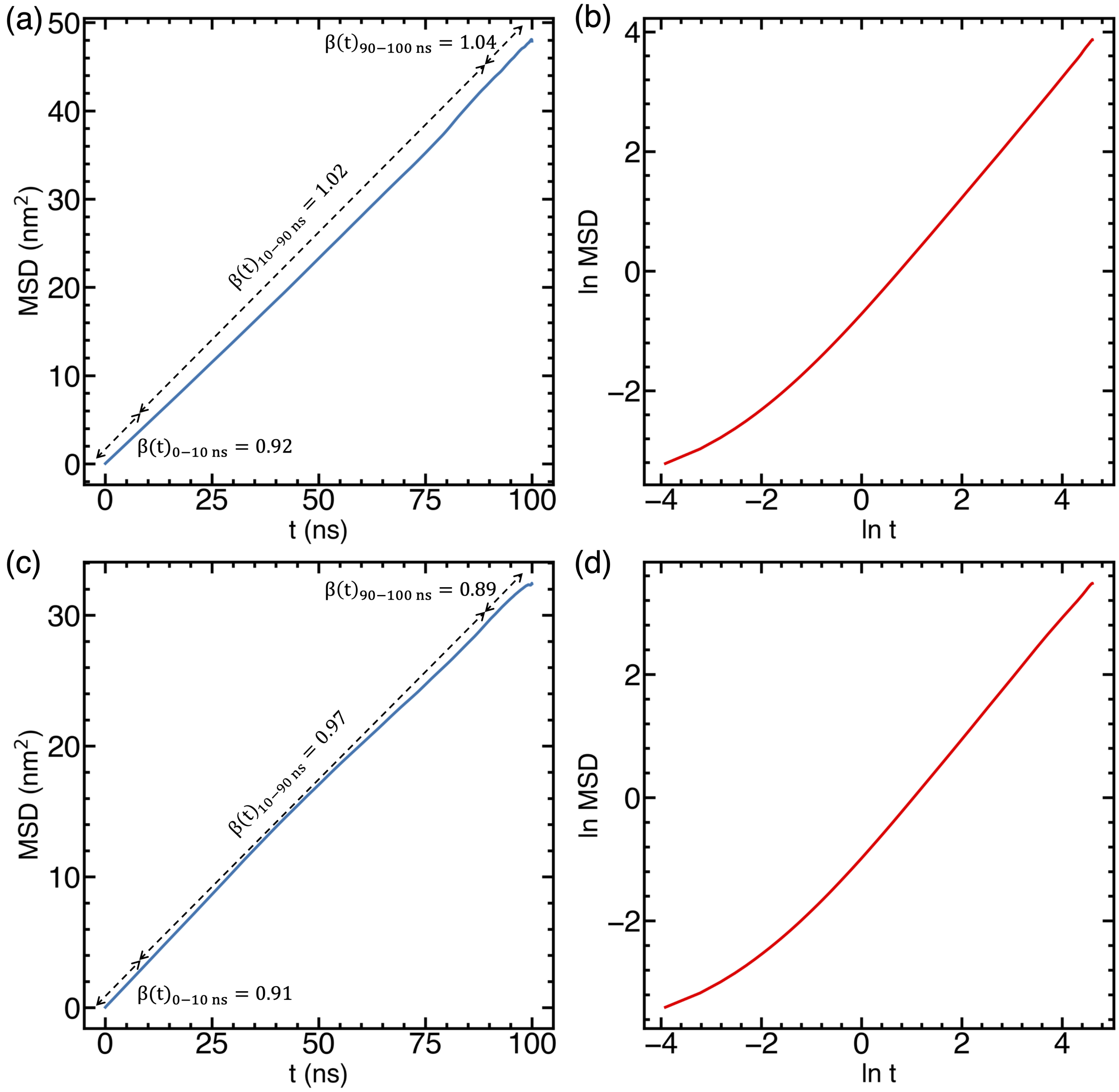

Figure S5: Mean-squared-displacement (MSD) as a function of time and ln MSD vs. ln t for $[C_2mim]^+$ (top pane) and for $[NTF_2]^-$ (bottom pane) for pure $[C_2mim][NTF_2]$ at 298 K modeled using cp2k-charges.

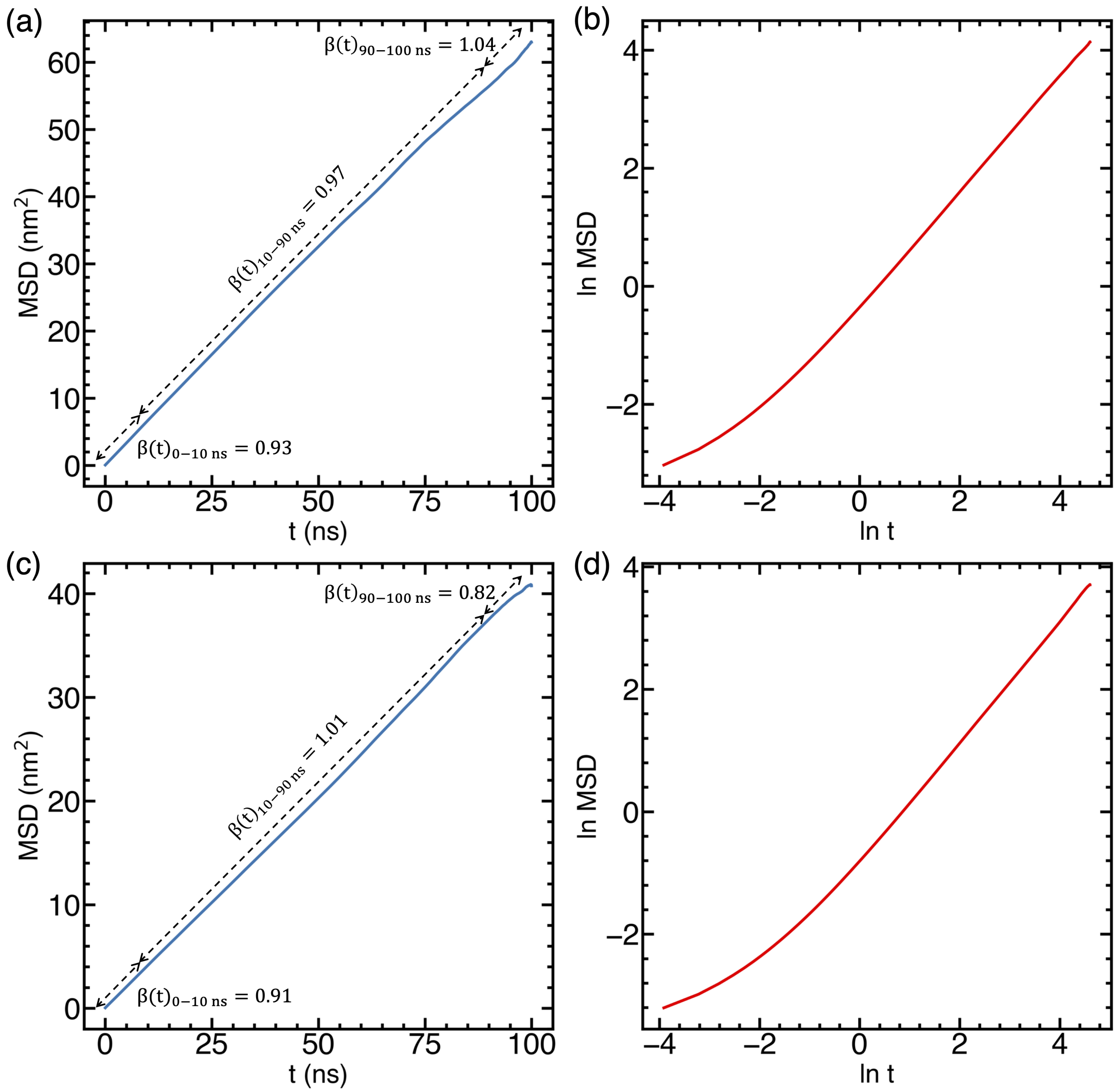

Figure S6: Mean-squared-displacement (MSD) as a function of time and ln MSD vs. ln t for $[C_2mim]^+$ (top pane) and for $[TFO]^-$ (bottom pane) for pure $[C_2mim][TFO]$ at 298 K modeled using cp2k-charges.

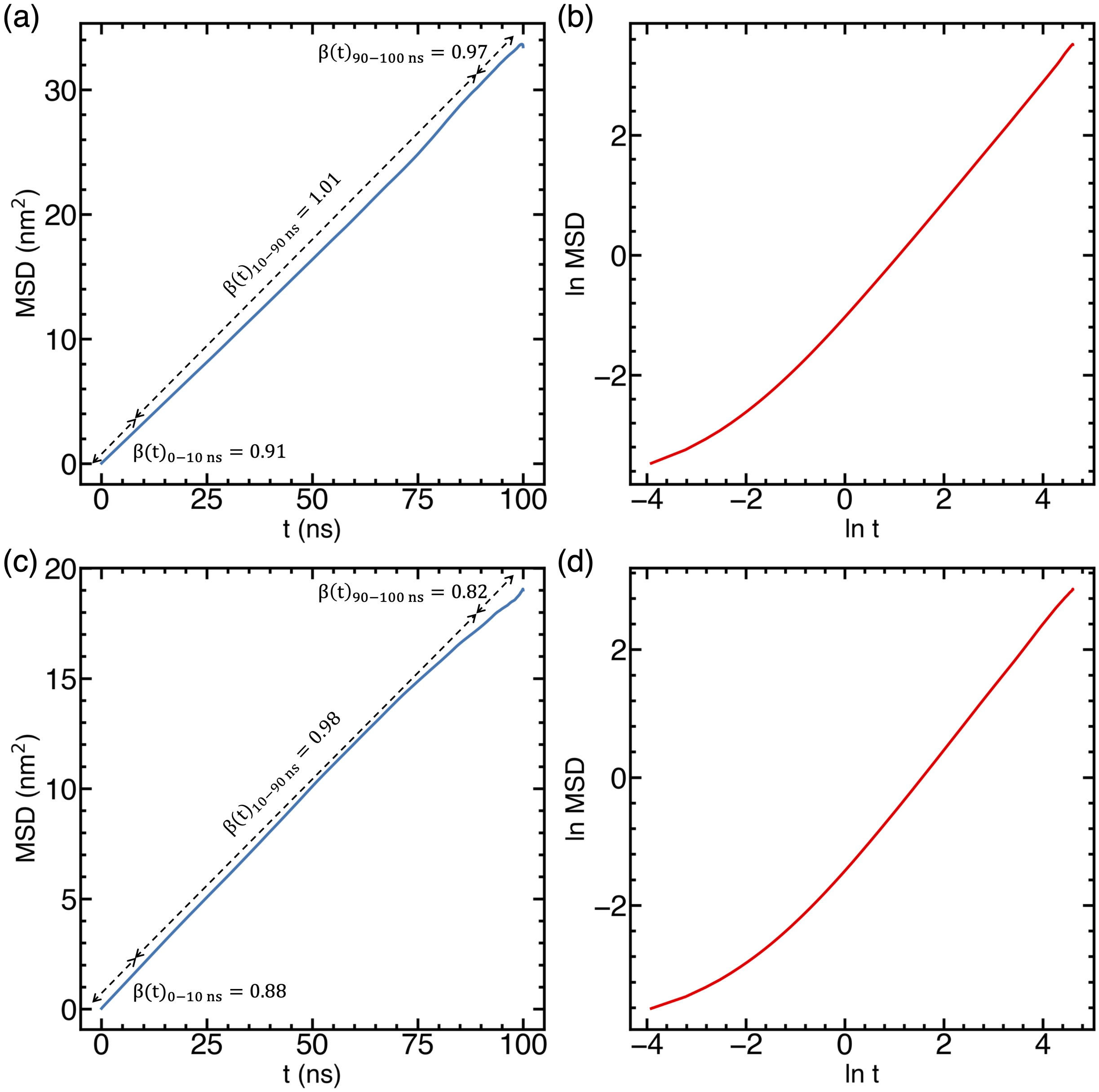

Figure S7: Mean-squared-displacement (MSD) as a function of time and ln MSD vs. ln t for $[C_2mim]^+$ (top pane) and for $[BF_4]^-$ (bottom pane) for pure $[C_2mim][BF_4]$ at 298 K modeled using VSIL.

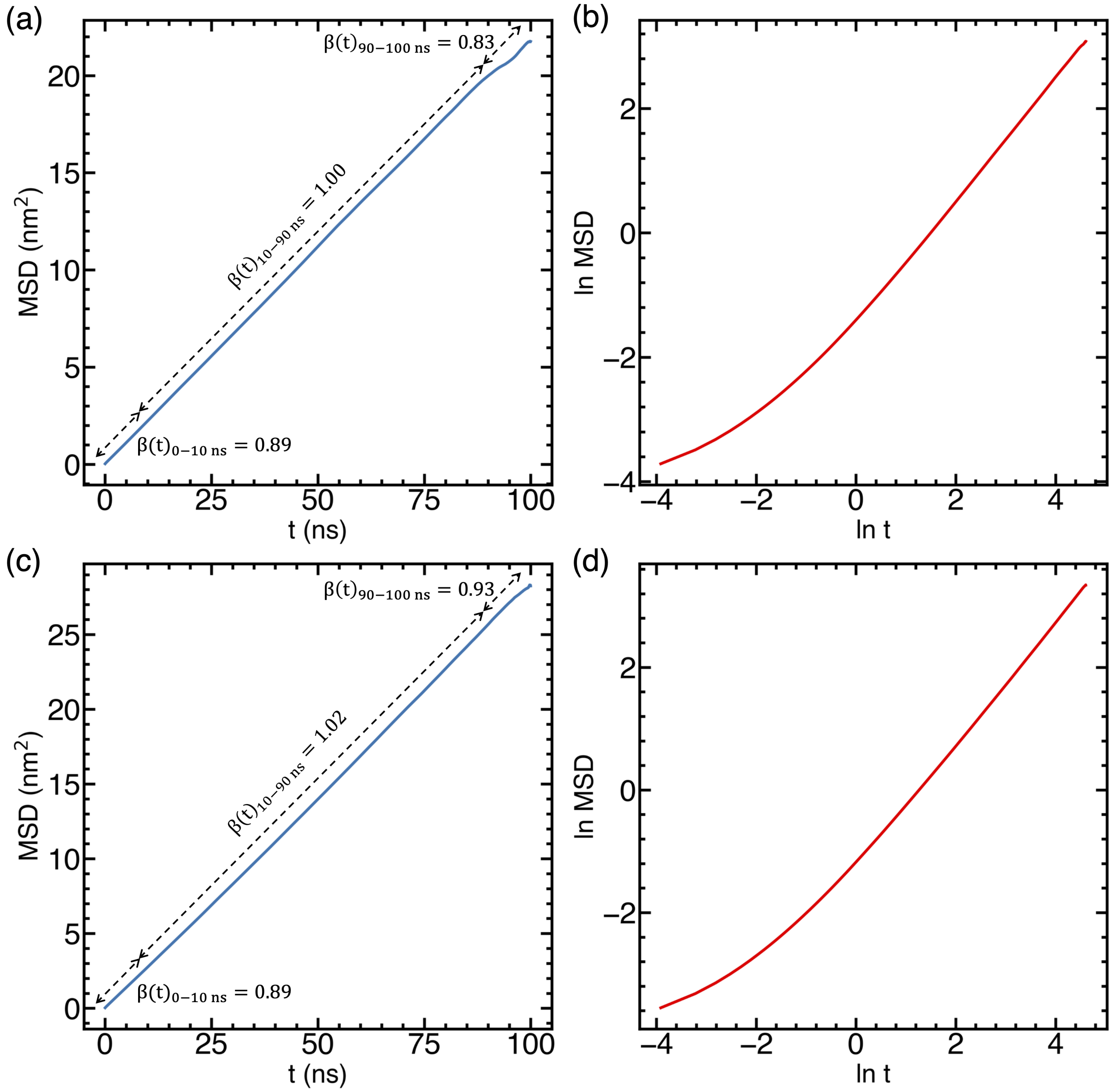

Figure S8: Mean-squared-displacement (MSD) as a function of time and ln MSD vs. ln t for $[C_2mim]^+$ (top pane) and for $[DCA]^-$ (bottom pane) for pure $[C_2mim][DCA]$ at 298 K modeled using VSIL.

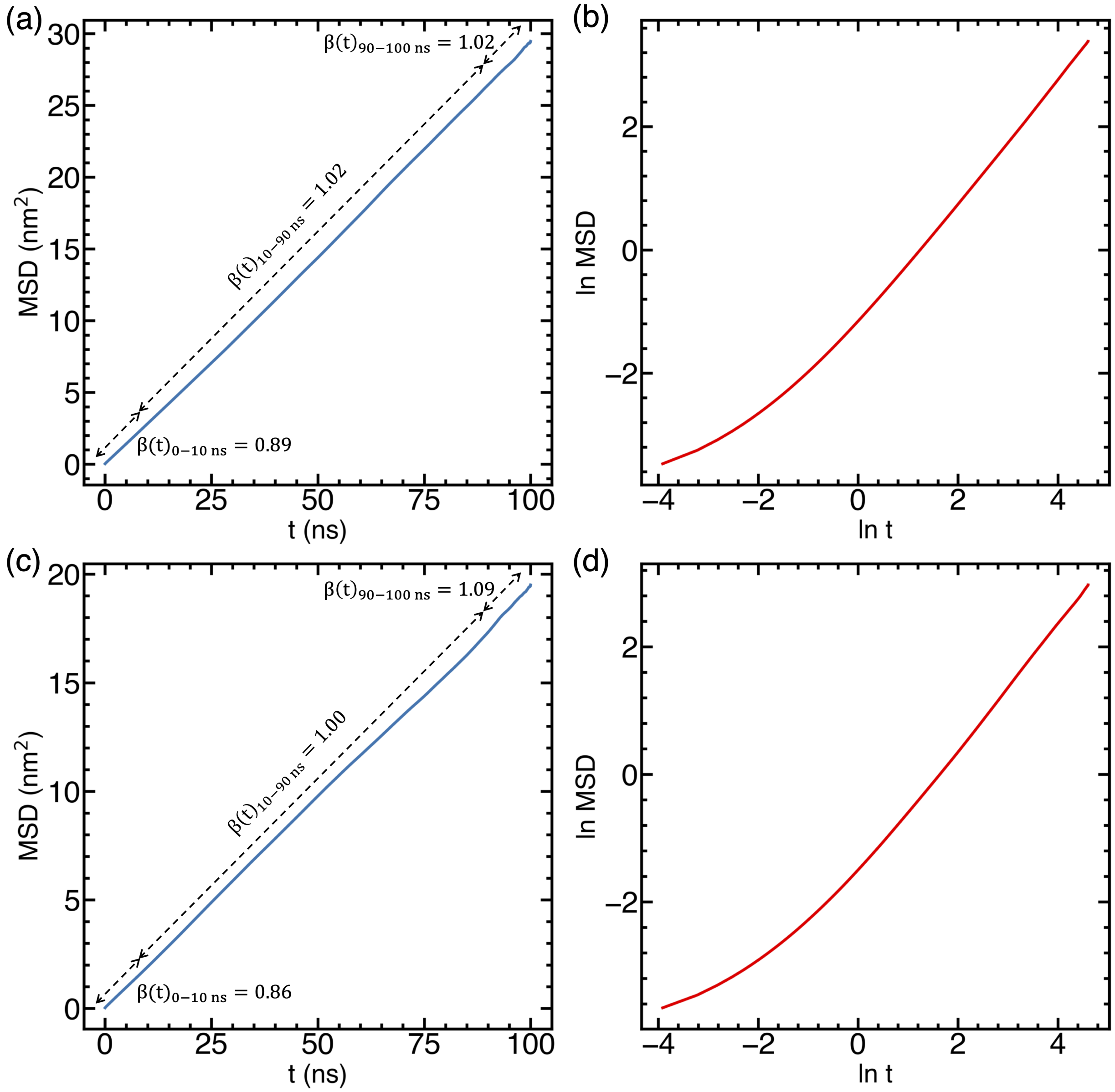

Figure S9: Mean-squared-displacement (MSD) as a function of time and ln MSD vs. ln t for $[C_2mim]^+$ (top pane) and for $[NTF_2]^-$ (bottom pane) for pure $[C_2mim][NTF_2]$ at 298 K modeled using VSIL.

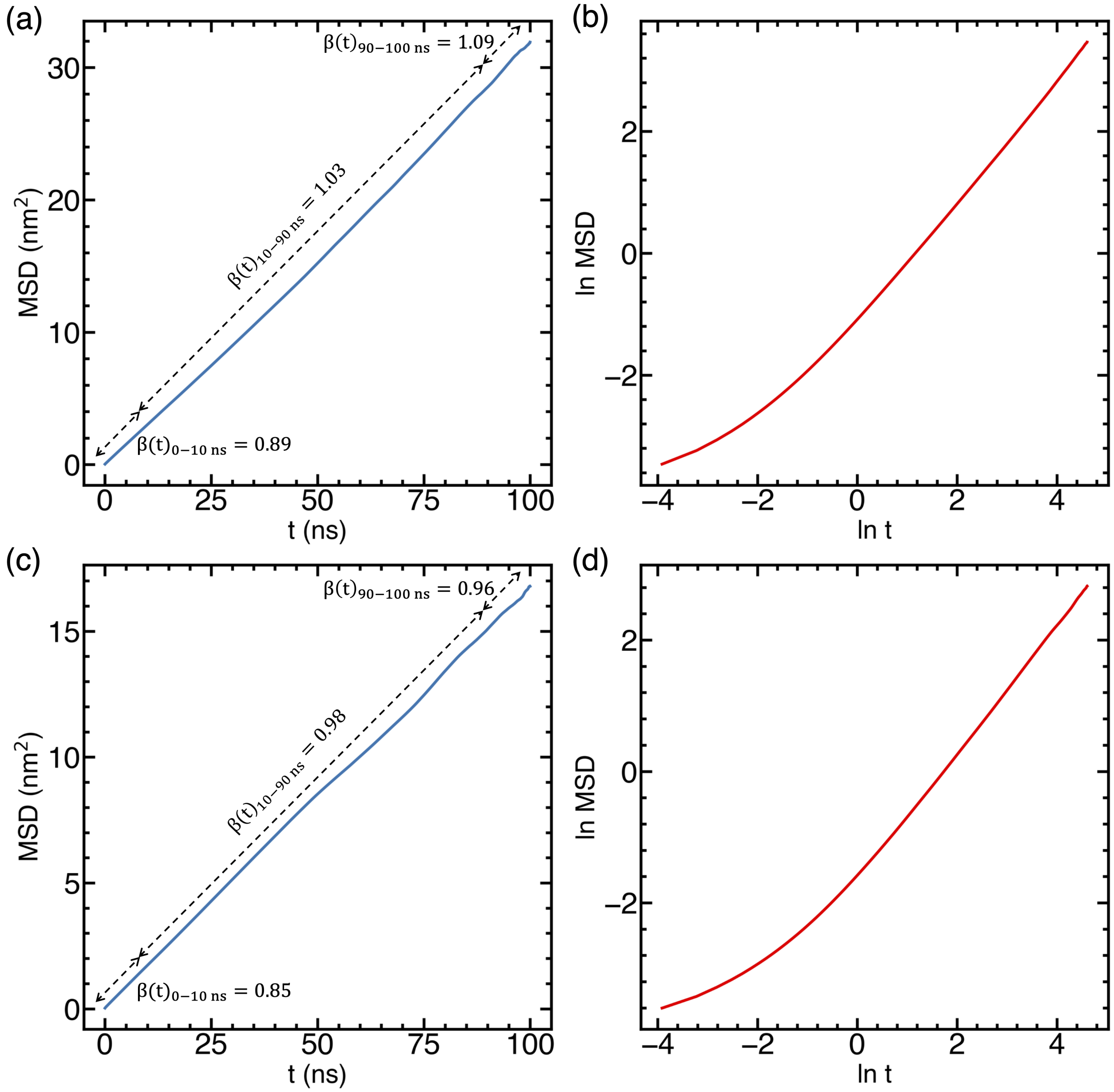

Figure S10: Mean-squared-displacement (MSD) as a function of time and ln MSD vs. ln t for $[C_2mim]^+$ (top pane) and for $[TFO]^-$ (bottom pane) for pure $[C_2mim][TFO]$ at 298 K modeled using VSIL.

Table S2: Self-diffusion coefficients (10^{-11} m^2/s) of cation and anions predicted from the force field proposed in this work and those determined with the VSIL force field.[10] *a*: Experimental values using VFT,[11] *b*: Simulated values by Haskins et al.,[12] *c*: Simulated values by Borodin,[13] *d*: Simulated values by Yang et al.,[14] *e*: Experimental values by Pitawela and Shaw.[15]

IL	cp2k-charges		VSIL		Reported	
	D^+	D^-	D^+	D^-	D^+	D^-
$[C_2mim][BF_4]$	3.4 ± 0.1	2.0 ± 0.1	5.5 ± 0.1	3.3 ± 0.1	4.97^a, 7^b	4.16^a, 6^b
$[C_2mim][DCA]$	8.6 ± 0.2	10.7 ± 0.2	3.7 ± 0.1	4.5 ± 0.2	9.0^c	14.1^c
$[C_2mim][NTF_2]$	7.4 ± 0.4	5.6 ± 0.3	4.7 ± 0.2	3.2 ± 0.1	6.3^a, 2.8^d	3.5^a, 3.8^d
$[C_2mim][TFO]$	10.7 ± 0.2	6.8 ± 0.2	5.0 ± 0.3	2.7 ± 0.1	4.3^e	2.6^e

S2.3 Nernst-Einstein and Einstein Conductivity

Table S3: Nernst-Einstein conductivity (S/m) of pure ionic liquids at 298 K.

ILs →	$[C_2mim][BF_4]$	$[C_2mim][DCA]$	$[C_2mim][NTF_2]$	$[C_2mim][TFO]$
cp2k-charges	1.03 ± 0.01	2.02 ± 0.01	1.04 ± 0.01	1.87 ± 0.00
VSIL	1.38 ± 0.01	1.31 ± 0.01	0.79 ± 0.00	1.07 ± 0.01

Table S4: Einstein conductivity (S/m) of pure ionic liquids at 298 K.

ILs →	$[C_2mim][BF_4]$	$[C_2mim][DCA]$	$[C_2mim][NTF_2]$	$[C_2mim][TFO]$
cp2k-charges	1.01 ± 0.08	1.50 ± 0.09	0.85 ± 0.03	1.59 ± 0.01
VSIL	1.24 ± 0.08	0.88 ± 0.02	0.64 ± 0.06	0.88 ± 0.04

S2.4 Radial Distribution Functions

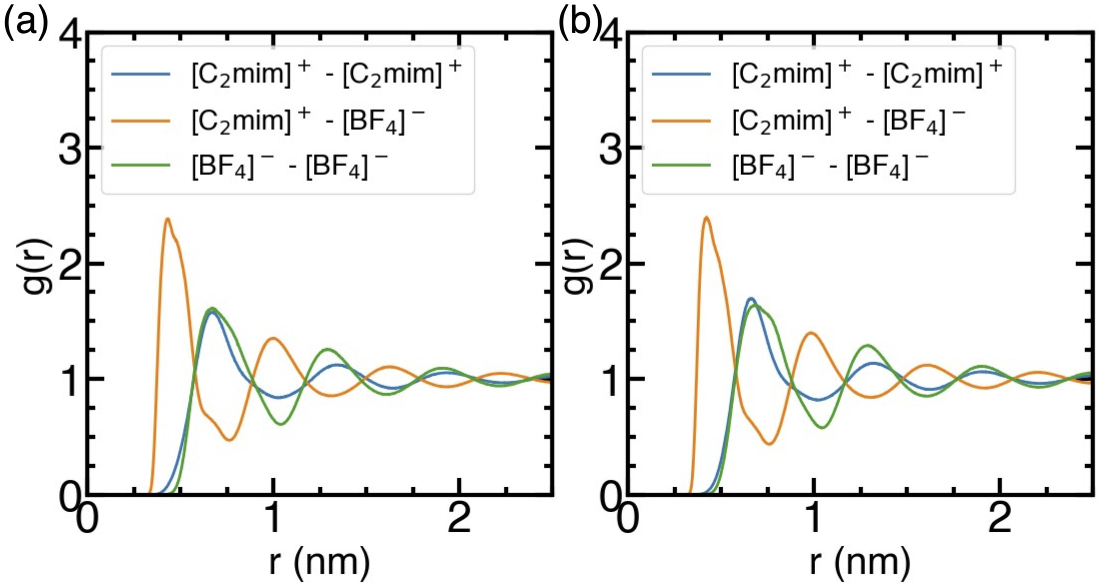

Figure S11: COM-COM RDFs depicting the cation-cation, cation-anion, and anion-anion interactions in the $[C_2mim][BF_4]$, computed using VSIL (a) and cp2k-charges (b).

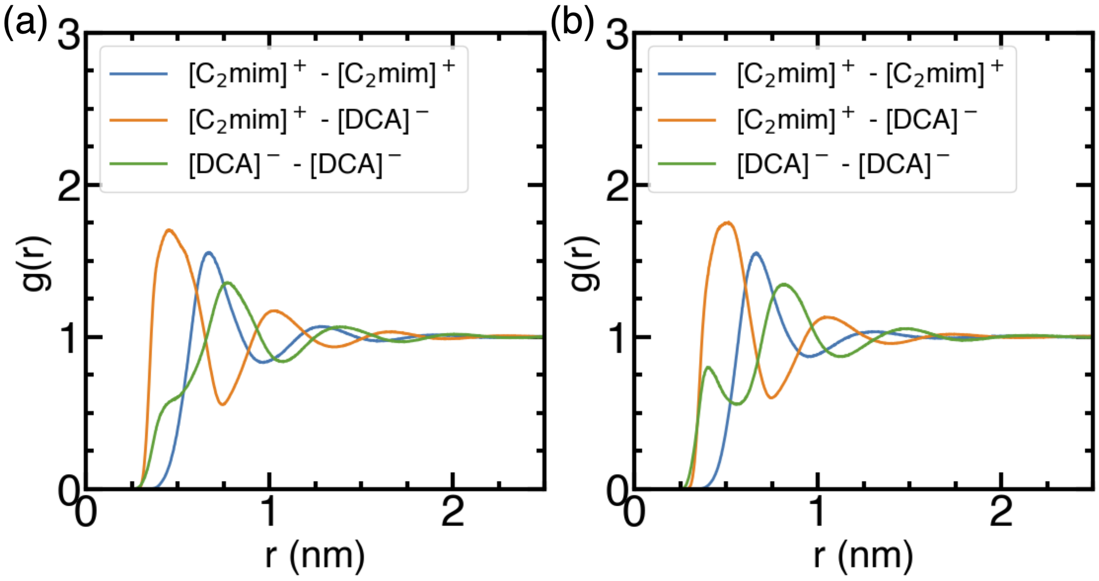

Figure S12: COM-COM RDFs depicting the cation-cation, cation-anion, and anion-anion interactions in the $[C_2mim]$[DCA], computed using VSIL (a) and cp2k-charges (b).

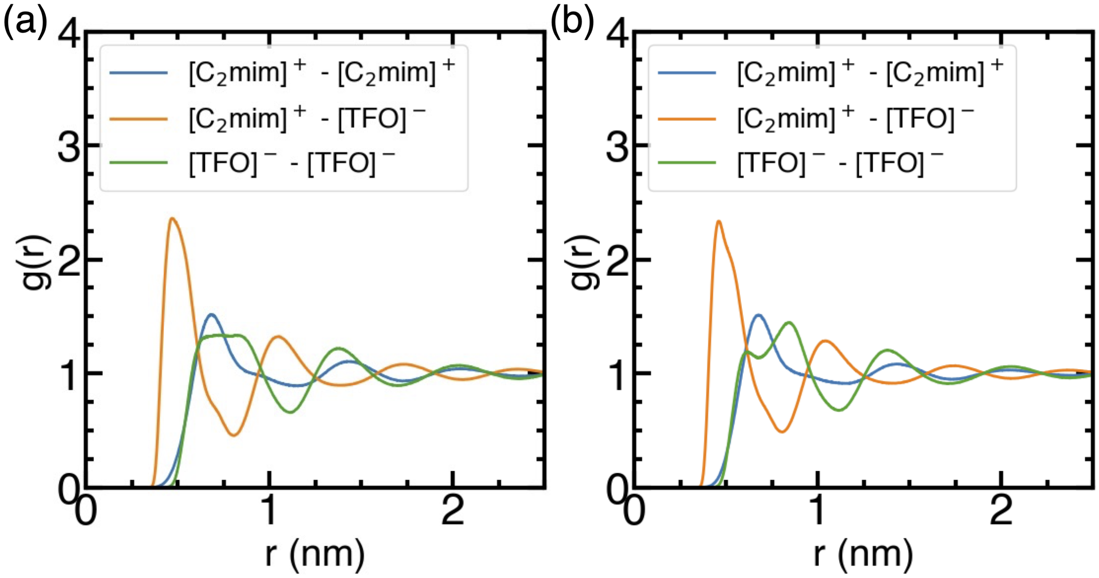

Figure S13: COM-COM RDFs depicting the cation-cation, cation-anion, and anion-anion interactions in the $[C_2mim][TFO]$ computed using VSIL (a) and cp2k-charges (b).

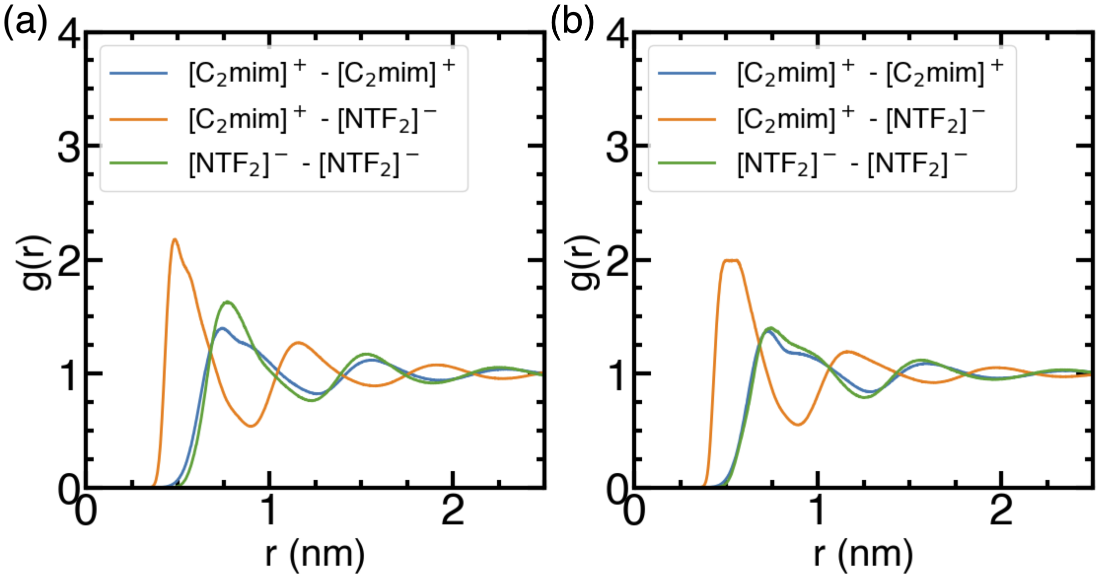

Figure S14: COM-COM RDFs depicting the cation-cation, cation-anion, and anion-anion interactions in the $[C_2mim][NTF_2]$, computed using VSIL (a) and cp2k-charges (b).

S3 Binary Ionic Liquid Mixtures

S3.1 Density

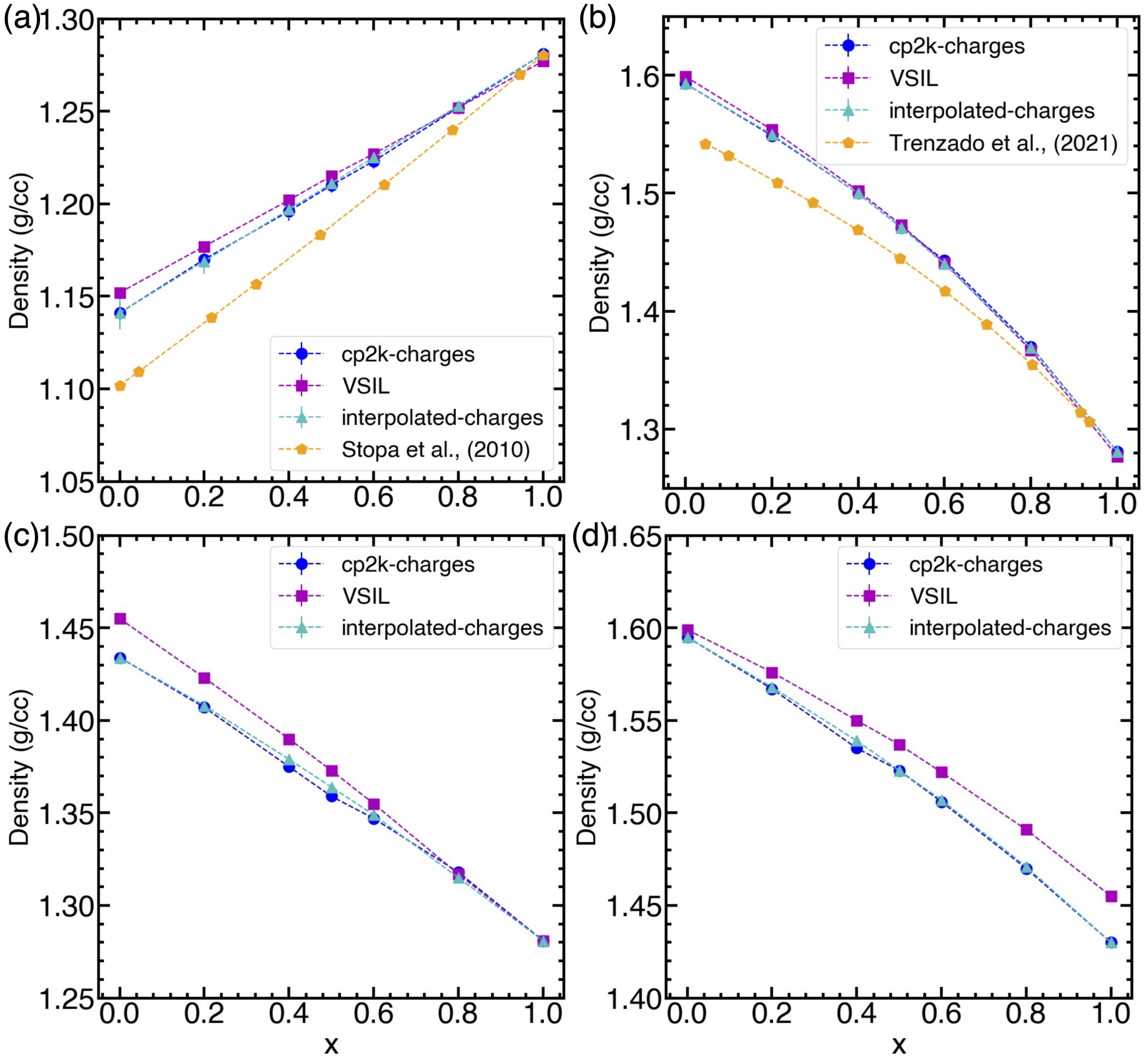

Figure S15: Liquid phase density of IL mixture at 298 K as a function of compositions. (a) $[C_2mim][BF_4]_x[DCA]_{(1-x)}$, (b) $[C_2mim][BF_4]_x[NTF_2]_{(1-x)}$, (c) $[C_2mim][BF_4]_x[TFO]_{(1-x)}$, and (d) $[C_2mim][TFO]_x[NTF_2]_{(1-x)}$. The solid lines represent experimentally observed density, and the dashed lines are only to act as a visual guide.

Table S5: Density of $[C_2mim][BF_4]_x[DCA]_{(1-x)}$ mixture with varying concentration.

Density (kg/m^3) → Compositions (x) ↓	VSIL	cp2k-charges	Interpolated-charges
0.0	1151.750 ± 0.078	1136.153 ± 0.021	1136.153 ± 0.021
0.2	1176.980 ± 0.056	1166.887 ± 0.046	1164.930 ± 0.044
0.4	1202.037 ± 0.045	1193.617 ± 0.035	1193.923 ± 0.058
0.5	1214.530 ± 0.030	1207.970 ± 0.082	1208.510 ± 0.082
0.6	1227.023 ± 0.058	1220.417 ± 0.101	1223.233 ± 0.021
0.8	1252.073 ± 0.047	1251.743 ± 0.078	1252.947 ± 0.012
1.0	1277.390 ± 0.121	1280.907 ± 0.118	1280.907 ± 0.118

Table S6: Density of $[C_2mim][BF_4]_x[NTF_2]_{(1-x)}$ mixture with varying concentration.

Density (kg/m^3) → Compositions (x) ↓	VSIL	cp2k-charges	Interpolated-charges
0.0	1599.143 ± 0.031	1595.023 ± 0.086	1595.023 ± 0.086
0.2	1554.153 ± 0.023	1548.503 ± 0.012	1549.997 ± 0.045
0.4	1502.017 ± 0.084	1500.213 ± 0.040	1499.733 ± 0.057
0.5	1472.687 ± 0.038	1471.473 ± 0.148	1471.263 ± 0.076
0.6	1440.647 ± 0.076	1442.557 ± 0.049	1440.240 ± 0.053
0.8	1367.220 ± 0.000	1370.400 ± 0.044	1368.727 ± 0.099
1.0	1277.390 ± 0.121	1280.907 ± 0.118	1280.907 ± 0.118

Table S7: Density of $[C_2mim][BF_4]_x[TFO]_{(1-x)}$ mixture with varying concentration.

Density (kg/m^3) → Compositions (x) ↓	VSIL	cp2k-charges	Interpolated-charges
0.0	1454.830 ± 0.026	1434.150 ± 0.035	1434.150 ± 0.035
0.2	1423.457 ± 0.045	1407.260 ± 0.040	1407.633 ± 0.078
0.4	1390.193 ± 0.136	1374.697 ± 0.074	1379.130 ± 0.020
0.5	1372.797 ± 0.091	1359.150 ± 0.061	1364.103 ± 0.032
0.6	1354.877 ± 0.107	1346.733 ± 0.067	1348.563 ± 0.038
0.8	1317.370 ± 0.044	1317.727 ± 0.055	1315.450 ± 0.078
1.0	1277.390 ± 0.121	1280.907 ± 0.118	1280.907 ± 0.118

Table S8: Density of $[C_2mim][TFO]_x[NTF_2]_{(1-x)}$ mixture with varying concentration.

Density (kg/m^3) → Compositions (x) ↓	VSIL	cp2k-charges	Interpolated-charges
0.0	1599.143 ± 0.031	1595.023 ± 0.086	1595.023 ± 0.086
0.2	1575.993 ± 0.176	1567.220 ± 0.072	1568.357 ± 0.045
0.4	1550.490 ± 0.101	1534.950 ± 0.040	1539.147 ± 0.059
0.5	1536.697 ± 0.035	1522.767 ± 0.021	1523.310 ± 0.080
0.6	1522.120 ± 0.131	1505.533 ± 0.035	1506.777 ± 0.038
0.8	1490.507 ± 0.059	1470.193 ± 0.021	1470.903 ± 0.085
1.0	1454.830 ± 0.026	1434.150 ± 0.035	1434.150 ± 0.035

S3.2 Molar Volume

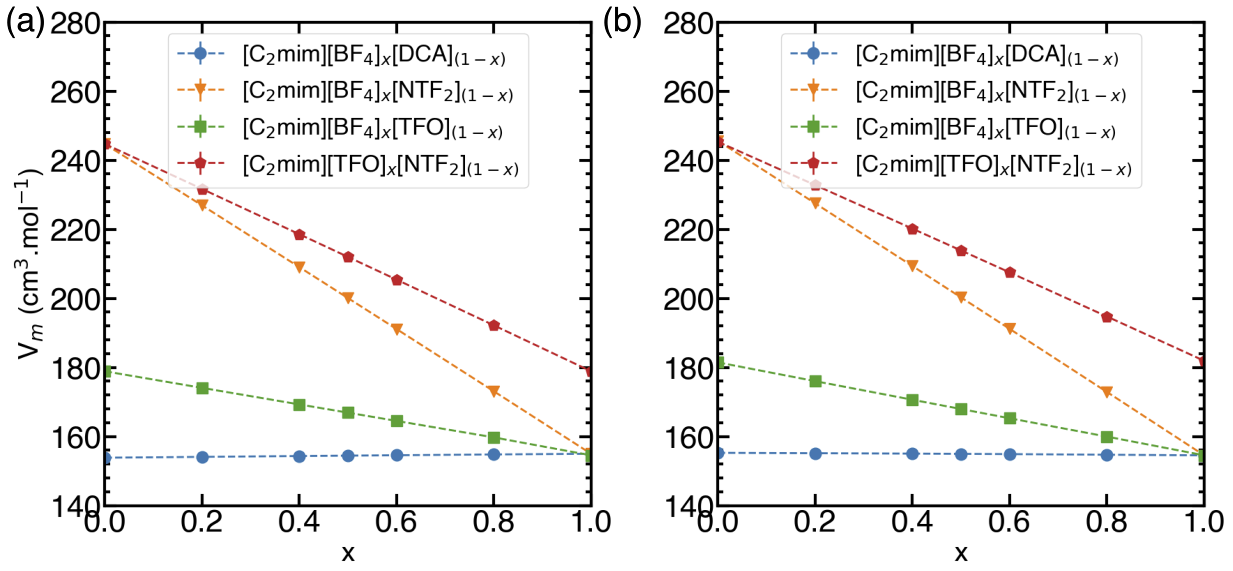

Figure S16: Molar volumes for the binary ionic liquid mixture systems as a function of concentration at 298 K computed using VSIL (a) and interpolated-charges (b).

S3.3 Excess Molar Volume

Excess molar volume is defined as the difference between the simulated mixture's molar volume and the ideal mixing molar volume. Mathematically, excess molar volume is expressed as

$$V_m^E = \frac{M_{mix}}{\rho_{mix}} - x_1\frac{M_1}{\rho_1} - x_2\frac{M_2}{\rho_2} \tag{S2}$$

where ρ_i, x_i, and M_i are the density, composition, and molecular weight of the ith ILs. Note that the M_{mix} can be expressed as $M_{mix} = x_1 M_1 + x_2 M_2$.

Calculations of excess molar volumes provide a clue into the packing efficiency of IL mixtures and calculated using Eq. S2. Typically, IL mixtures exhibit ideal behavior in terms of volume mixing with small excess molar volumes. This was also observed for all the systems studied in this work (Figure S17). The small differences arise either due to the difference in the hydrogen bonding ability of the anions or the difference in molar volumes of ILs, as suggested by Kapoor and Shah.[16] Using the interpolated-charges and VSIL force field yield similar qualitative trends in excess molar volumes: (a) slight positive excess molar volumes for [C_2mim][BF_4][DCA], [C_2mim][BF_4][NTF_2], and [C_2mim][BF_4][TFO]; (b) negative excess molar volumes for [C_2mim][TFO][NTF_2]. Moreover, the slightly positive excess molar volume observed for [C_2mim][BF_4][NTf_2] aligns well with the findings reported by Trenzado et al.[17] and exhibits maximum at an equimolar composition.

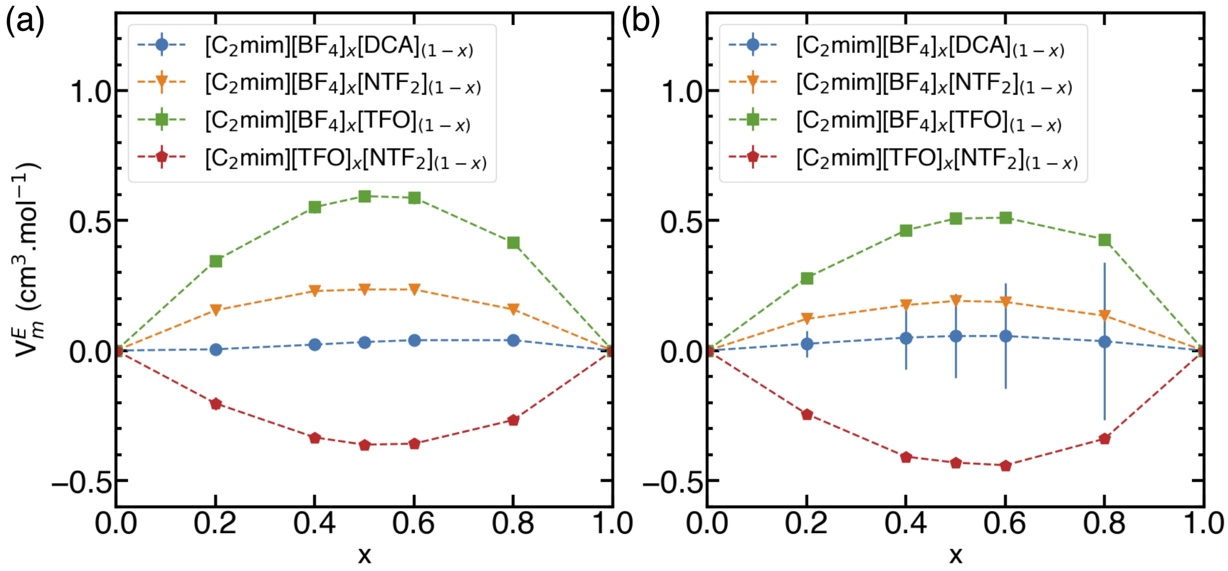

Figure S17: Excess molar volume of IL mixtures at 298 K as a function of concentration, computed using (a) VSIL, and (b) interpolated-charges. The dashed lines only act as a visual guide.

S3.4 Self-Diffusion Coefficients

Table S9: Self-diffusivity of ions in $[C_2mim][BF_4]_x[DCA]_{(1-x)}$ mixture with varying concentration.

D x 10^{11} (m^2/s) →	VSIL			cp2k-charges			Interpolated-charges		
Compositions (x) ↓	$D^+_{C_2mim}$	$D^-_{BF_4}$	D^-_{DCA}	$D^+_{C_2mim}$	$D^-_{BF_4}$	D^-_{DCA}	$D^+_{C_2mim}$	$D^-_{BF_4}$	D^-_{DCA}
0.0	3.68 ± 0.08		4.59 ± 0.11	8.67 ± 0.12		10.78 ± 0.10	8.67 ± 0.12		10.78 ± 0.10
0.2	4.13 ± 0.08	3.42 ± 0.21	4.97 ± 0.15	6.62 ± 0.05	5.21 ± 0.15	8.43 ± 0.05	7.83 ± 0.07	5.28 ± 0.22	10.09 ± 0.26
0.4	4.34 ± 0.12	3.74 ± 0.17	5.22 ± 0.25	6.64 ± 0.23	5.06 ± 0.14	9.19 ± 0.15	6.67 ± 0.10	4.89 ± 0.28	8.52 ± 0.35
0.5	4.81 ± 0.08	3.82 ± 0.16	5.27 ± 0.07	5.96 ± 0.11	4.56 ± 0.11	7.39 ± 0.10	6.12 ± 0.14	4.26 ± 0.10	8.21 ± 0.08
0.6	4.82 ± 0.17	3.66 ± 0.10	5.14 ± 0.11	6.05 ± 0.16	4.34 ± 0.17	7.60 ± 0.30	5.65 ± 0.07	4.07 ± 0.08	7.15 ± 0.22
0.8	5.21 ± 0.04	3.64 ± 0.03	5.19 ± 0.07	4.77 ± 0.10	3.15 ± 0.20	6.37 ± 0.38	4.76 ± 0.14	2.98 ± 0.13	6.01 ± 0.33
1.0	5.40 ± 0.08	3.35 ± 0.03		3.46 ± 0.05	2.00 ± 0.03		3.46 ± 0.05	2.00 ± 0.03	

Table S10: Self-diffusivity of ions in $[C_2mim][BF_4]_x[NTF_2]_{(1-x)}$ mixture with varying concentration.

D x 10^{11} (m^2/s) →	VSIL			cp2k-charges			Interpolated-charges		
Compositions (x) ↓	$D^+_{C_2mim}$	$D^-_{BF_4}$	$D^-_{NTF_2}$	$D^+_{C_2mim}$	$D^-_{BF_4}$	$D^-_{NTF_2}$	$D^+_{C_2mim}$	$D^-_{BF_4}$	$D^-_{NTF_2}$
0.0	4.71 ± 0.09		3.11 ± 0.10	7.65 ± 0.10		5.73 ± 0.03	7.65 ± 0.10		5.73 ± 0.03
0.2	4.61 ± 0.08	2.82 ± 0.06	3.20 ± 0.05	6.76 ± 0.19	4.37 ± 0.19	5.48 ± 0.18	6.51 ± 0.18	4.12 ± 0.10	5.23 ± 0.05
0.4	4.55 ± 0.17	2.69 ± 0.06	3.15 ± 0.12	5.69 ± 0.12	3.87 ± 0.15	4.32 ± 0.16	5.72 ± 0.12	3.55 ± 0.02	4.48 ± 0.03
0.5	4.61 ± 0.05	2.99 ± 0.13	2.99 ± 0.20	5.51 ± 0.09	3.62 ± 0.13	4.11 ± 0.20	5.50 ± 0.14	3.25 ± 0.10	3.78 ± 0.15
0.6	4.54 ± 0.23	2.87 ± 0.05	3.01 ± 0.06	5.06 ± 0.08	3.16 ± 0.21	3.65 ± 0.07	5.09 ± 0.24	3.26 ± 0.17	3.74 ± 0.11
0.8	4.92 ± 0.09	3.05 ± 0.15	3.10 ± 0.09	4.25 ± 0.04	2.64 ± 0.02	2.73 ± 0.15	4.64 ± 0.11	2.82 ± 0.09	3.01 ± 0.09
1.0	5.40 ± 0.08	3.35 ± 0.03		3.46 ± 0.05	2.00 ± 0.03		3.46 ± 0.05	2.00 ± 0.03	

Table S11: Self-diffusivity of ions in $[C_2mim][BF_4]_x[TFO]_{(1-x)}$ mixture with varying concentration.

D x 10^{11} (m^2/s) →	VSIL			cp2k-charges			Interpolated-charges		
Compositions (x) ↓	$D^+_{C_2mim}$	$D^-_{BF_4}$	D^-_{TFO}	$D^+_{C_2mim}$	$D^-_{BF_4}$	D^-_{TFO}	$D^+_{C_2mim}$	$D^-_{BF_4}$	D^-_{TFO}
0.0	4.96 ± 0.15		2.76 ± 0.10	10.91 ± 0.08		6.72 ± 0.17	10.91 ± 0.08		6.72 ± 0.17
0.2	4.91 ± 0.35	3.14 ± 0.17	2.67 ± 0.09	8.17 ± 0.15	4.63 ± 0.05	5.33 ± 0.13	8.30 ± 0.22	4.51 ± 0.27	5.05 ± 0.07
0.4	5.00 ± 0.07	3.20 ± 0.20	3.08 ± 0.16	7.98 ± 0.16	4.99 ± 0.07	5.32 ± 0.12	7.07 ± 0.08	4.06 ± 0.05	4.31 ± 0.13
0.5	5.05 ± 0.04	3.08 ± 0.12	2.92 ± 0.06	7.38 ± 0.12	4.61 ± 0.14	4.91 ± 0.27	6.46 ± 0.08	3.76 ± 0.07	3.95 ± 0.09
0.6	5.16 ± 0.02	3.17 ± 0.13	2.89 ± 0.17	6.01 ± 0.03	3.74 ± 0.13	3.71 ± 0.22	5.81 ± 0.17	3.42 ± 0.05	3.70 ± 0.09
0.8	5.28 ± 0.12	3.29 ± 0.11	3.06 ± 0.09	4.57 ± 0.19	2.62 ± 0.13	2.62 ± 0.19	4.88 ± 0.08	2.75 ± 0.08	3.01 ± 0.12
1.0	5.40 ± 0.08	3.35 ± 0.03		3.46 ± 0.05	2.00 ± 0.03		3.46 ± 0.05	2.00 ± 0.03	

Table S12: Self-diffusivity of ions in $[C_2mim][TFO]_x[NTF_2]_{(1-x)}$ mixture with varying concentration.

D x 10^{11} (m^2/s) →	VSIL			cp2k-charges			Interpolated-charges		
Compositions (x) ↓	$D^+_{C_2mim}$	$D^-_{NTF_2}$	D^-_{TFO}	$D^+_{C_2mim}$	$D^-_{NTF_2}$	D^-_{TFO}	$D^+_{C_2mim}$	$D^-_{NTF_2}$	D^-_{TFO}
0.0	4.71 ± 0.09	3.11 ± 0.10		7.65 ± 0.10	5.73 ± 0.03		7.65 ± 0.10	5.73 ± 0.03	
0.2	4.65 ± 0.05	3.16 ± 0.05	3.05 ± 0.18	8.51 ± 0.12	6.47 ± 0.26	6.09 ± 0.36	8.16 ± 0.15	5.85 ± 0.14	6.37 ± 0.59
0.4	4.61 ± 0.24	3.15 ± 0.14	2.90 ± 0.11	9.65 ± 0.20	6.72 ± 0.16	6.47 ± 0.22	8.87 ± 0.12	6.35 ± 0.03	6.36 ± 0.11
0.5	4.79 ± 0.17	3.05 ± 0.13	2.91 ± 0.05	8.90 ± 0.11	6.22 ± 0.07	6.64 ± 0.05	9.21 ± 0.15	6.42 ± 0.08	6.21 ± 0.26
0.6	4.83 ± 0.13	3.14 ± 0.23	2.93 ± 0.14	10.29 ± 0.22	6.36 ± 0.11	6.94 ± 0.35	9.65 ± 0.23	6.29 ± 0.18	6.40 ± 0.11
0.8	4.92 ± 0.23	2.88 ± 0.17	2.80 ± 0.11	10.23 ± 0.06	6.55 ± 0.32	6.60 ± 0.14	10.44 ± 0.10	6.54 ± 0.00	6.70 ± 0.09
1.0	4.96 ± 0.15		2.76 ± 0.10	10.91 ± 0.08		6.72 ± 0.17	10.91 ± 0.08		6.72 ± 0.17

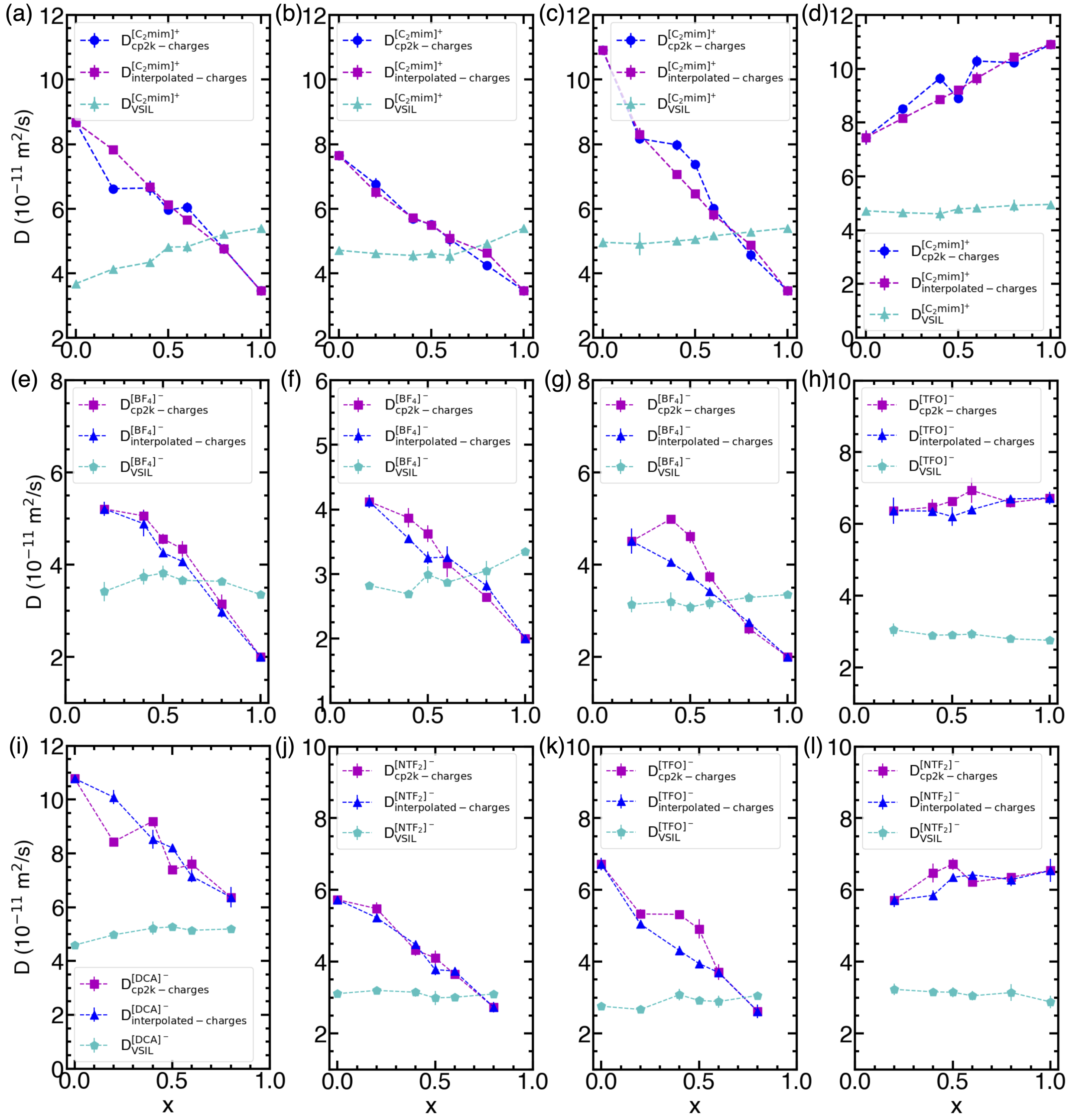

Figure S18: Self-diffusivity of cations and anions in $[C_2mim][BF_4]_x[DCA]_{(1-x)}$ (first column), $[C_2mim][BF_4]_x[NTF_2]_{(1-x)}$, (second column) $[C_2mim][BF_4]_x[TFO]_{(1-x)}$ (third column), and $[C_2mim][TFO]_x[NTF_2]_{(1-x)}$ (fourth column), with a focus on the cations (upper pane), first anions (middle pane), and second anions (lower pane) self-diffusivity as a function of compositions. The dashed lines are only to act as a visual guide.

S3.5 Nernst-Einstein and Einstein Ionic Conductivity

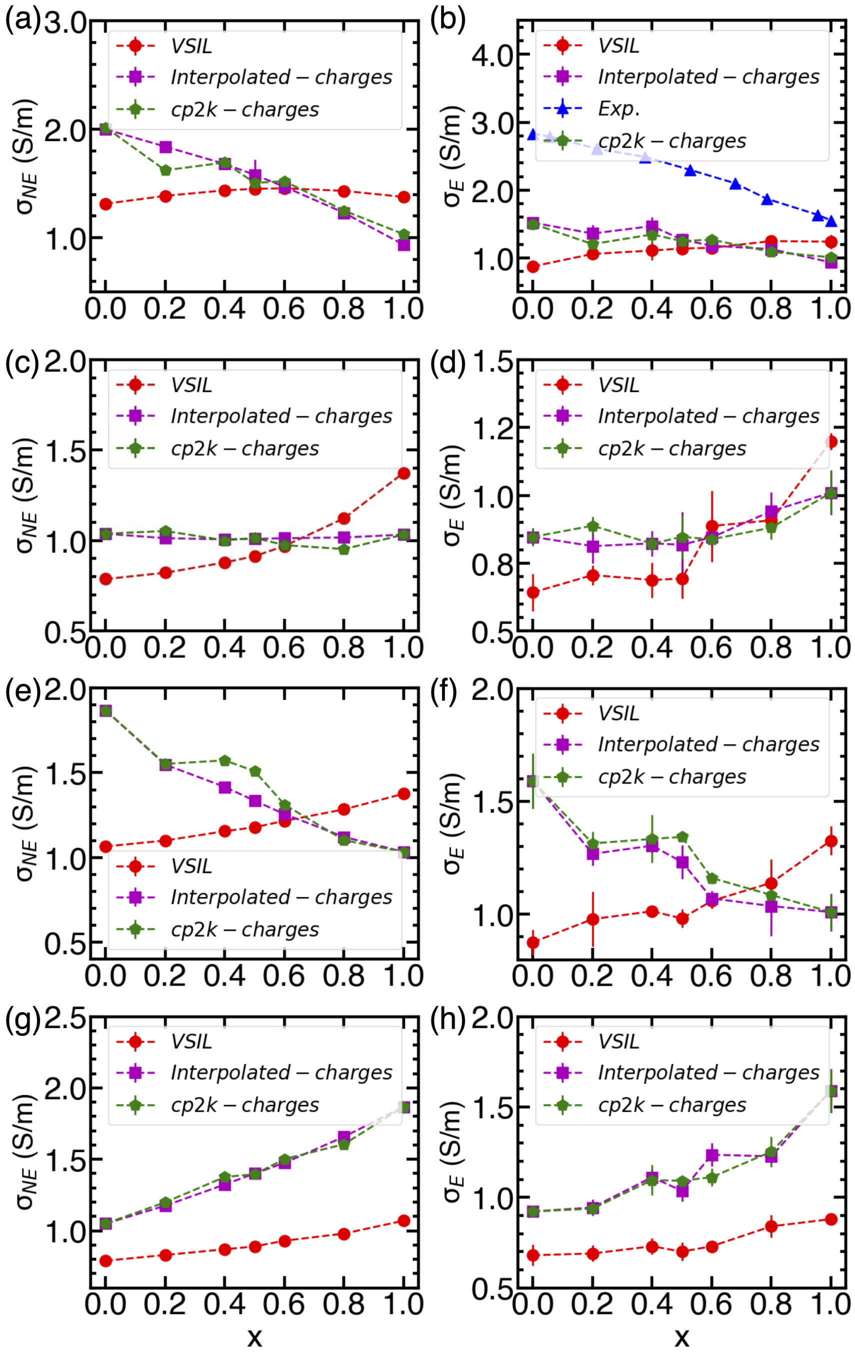

Figure S19: Nernst-Einstein (left pane) and Einstein (right pane) conductivity of (a, b) $[C_2mim][BF_4]_x[DCA]_{(1-x)}$, (c, d) $[C_2mim][BF_4]_x[NTF_2]_{(1-x)}$, (e, f) $[C_2mim][BF_4]_x[TFO]_{(1-x)}$, and (g, h) $[C_2mim][TFO]_x[NTF_2]_{(1-x)}$ as a function of compositions. The dashed lines are only to act as a visual guide.

Table S13: Ionic conductivity of $[C_2mim][BF_4]_x[DCA]_{(1-x)}$ mixture with varying concentration.

σ (S/m) →	VSIL		cp2k-charges		Interpolated-charges	
Compositions (x) ↓	σ_{NE}	σ_E	σ_{NE}	σ_E	σ_{NE}	σ_E
0.0	1.31 ± 0.01	0.88 ± 0.02	2.02 ± 0.01	1.50 ± 0.09	2.02 ± 0.01	1.50 ± 0.09
0.2	1.39 ± 0.01	1.06 ± 0.02	1.62 ± 0.00	1.20 ± 0.02	1.84 ± 0.01	1.36 ± 0.13
0.4	1.44 ± 0.01	1.11 ± 0.11	1.69 ± 0.00	1.35 ± 0.14	1.68 ± 0.00	1.47 ± 0.13
0.5	1.45 ± 0.00	1.14 ± 0.07	1.50 ± 0.00	1.25 ± 0.04	1.58 ± 0.01	1.27 ± 0.09
0.6	1.46 ± 0.01	1.15 ± 0.02	1.52 ± 0.01	1.27 ± 0.07	1.47 ± 0.00	1.18 ± 0.02
0.8	1.43 ± 0.00	1.25 ± 0.06	1.25 ± 0.01	1.09 ± 0.05	1.23 ± 0.01	1.13 ± 0.07
1.0	1.38 ± 0.01	1.24 ± 0.08	1.03 ± 0.01	1.01 ± 0.08	1.03 ± 0.01	1.01 ± 0.08

Table S14: Ionic conductivity of $[C_2mim][BF_4]_x[NTF_2]_{(1-x)}$ mixture with varying concentration.

σ (S/m) →	VSIL		cp2k-charges		Interpolated-charges	
Compositions (x) ↓	σ_{NE}	σ_E	σ_{NE}	σ_E	σ_{NE}	σ_E
0.0	0.79 ± 0.00	0.64 ± 0.06	1.04 ± 0.01	0.85 ± 0.03	1.04 ± 0.01	0.85 ± 0.03
0.2	0.83 ± 0.00	0.71 ± 0.03	1.05 ± 0.00	0.89 ± 0.03	1.01 ± 0.00	0.81 ± 0.06
0.4	0.88 ± 0.00	0.69 ± 0.05	1.00 ± 0.00	0.82 ± 0.03	1.01 ± 0.01	0.82 ± 0.05
0.5	0.91 ± 0.00	0.69 ± 0.06	1.02 ± 0.00	0.85 ± 0.08	1.01 ± 0.00	0.82 ± 0.12
0.6	0.97 ± 0.00	0.86 ± 0.07	0.98 ± 0.01	0.84 ± 0.01	1.01 ± 0.01	0.84 ± 0.03
0.8	1.12 ± 0.01	0.91 ± 0.02	0.95 ± 0.01	0.88 ± 0.04	1.02 ± 0.01	0.94 ± 0.07
1.0	1.38 ± 0.01	1.24 ± 0.08	1.03 ± 0.01	1.01 ± 0.08	1.03 ± 0.01	1.01 ± 0.08

Table S15: Ionic conductivity of $[C_2mim][BF_4]_x[TFO]_{(1-x)}$ mixture with varying concentration.

σ (S/m) →	VSIL		cp2k-charges		Interpolated-charges	
Compositions (x) ↓	σ_{NE}	σ_E	σ_{NE}	σ_E	σ_{NE}	σ_E
0.0	1.06 ± 0.01	0.88 ± 0.04	1.71 ± 0.00	1.40 ± 0.06	1.71 ± 0.00	1.40 ± 0.06
0.2	1.10 ± 0.01	0.98 ± 0.10	1.55 ± 0.01	1.31 ± 0.05	1.55 ± 0.01	1.27 ± 0.05
0.4	1.16 ± 0.01	0.99 ± 0.04	1.57 ± 0.01	1.33 ± 0.11	1.42 ± 0.01	1.30 ± 0.02
0.5	1.18 ± 0.01	0.98 ± 0.03	1.51 ± 0.01	1.34 ± 0.02	1.33 ± 0.00	1.23 ± 0.07
0.6	1.22 ± 0.01	1.06 ± 0.03	1.31 ± 0.00	1.16 ± 0.02	1.26 ± 0.02	1.07 ± 0.03
0.8	1.28 ± 0.01	1.14 ± 0.08	1.10 ± 0.00	1.09 ± 0.02	1.12 ± 0.00	1.04 ± 0.13
1.0	1.38 ± 0.01	1.24 ± 0.08	1.03 ± 0.01	1.01 ± 0.08	1.03 ± 0.01	1.01 ± 0.08

Table S16: Ionic conductivity of $[C_2mim][TFO]_x[NTF_2]_{(1-x)}$ mixture with varying concentration.

σ (S/m) →	VSIL		cp2k-charges		Interpolated-charges	
Compositions (x) ↓	σ_{NE}	σ_E	σ_{NE}	σ_E	σ_{NE}	σ_E
0.0	0.79 ± 0.00	0.64 ± 0.06	1.04 ± 0.01	0.85 ± 0.03	1.04 ± 0.01	0.85 ± 0.03
0.2	0.83 ± 0.01	0.69 ± 0.05	1.20 ± 0.01	0.94 ± 0.03	1.17 ± 0.00	0.94 ± 0.04
0.4	0.87 ± 0.00	0.73 ± 0.04	1.38 ± 0.00	1.10 ± 0.08	1.32 ± 0.01	1.11 ± 0.05
0.5	0.89 ± 0.01	0.70 ± 0.05	1.39 ± 0.01	1.09 ± 0.03	1.40 ± 0.01	1.04 ± 0.06
0.6	0.93 ± 0.01	0.73 ± 0.01	1.50 ± 0.00	1.11 ± 0.05	1.47 ± 0.00	1.24 ± 0.07
0.8	0.98 ± 0.01	0.84 ± 0.06	1.61 ± 0.01	1.25 ± 0.08	1.66 ± 0.01	1.23 ± 0.01
1.0	1.06 ± 0.01	0.88 ± 0.04	1.71 ± 0.00	1.40 ± 0.06	1.71 ± 0.00	1.40 ± 0.06

S3.6 Radial Distribution Functions

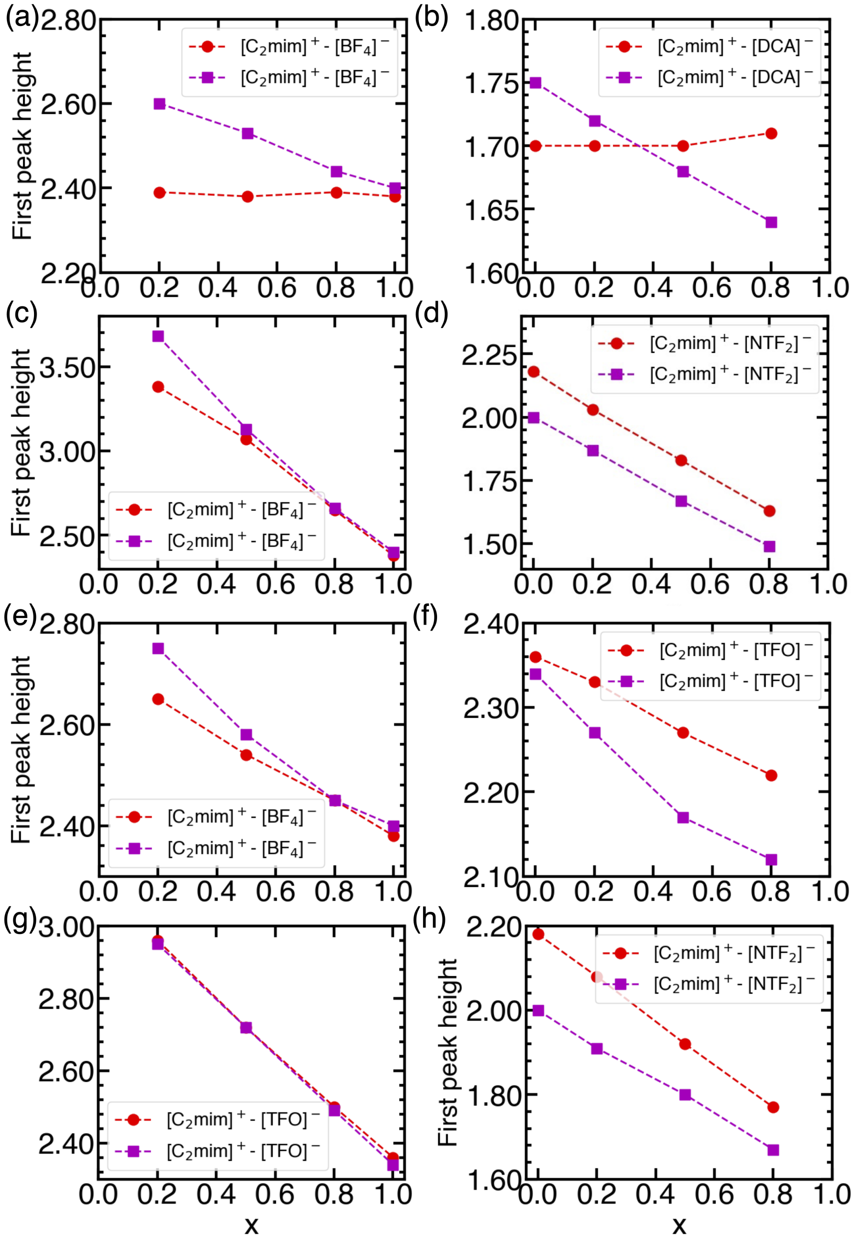

Figure S20: COM-COM cation-anion RDFs first peak height: (a, b) $[C_2mim][BF_4]_x[DCA]_{(1-x)}$, (c, d) $[C_2mim][BF_4]_x[NTF_2]_{(1-x)}$, (e, f) $[C_2mim][BF_4]_x[TFO]_{(1-x)}$ and (g, h) $[C_2mim][TFO]_x[NTF_2]_{(1-x)}$ mixtures with varying concentrations. The colors red and magenta represent VSIL and interpolated-charges.

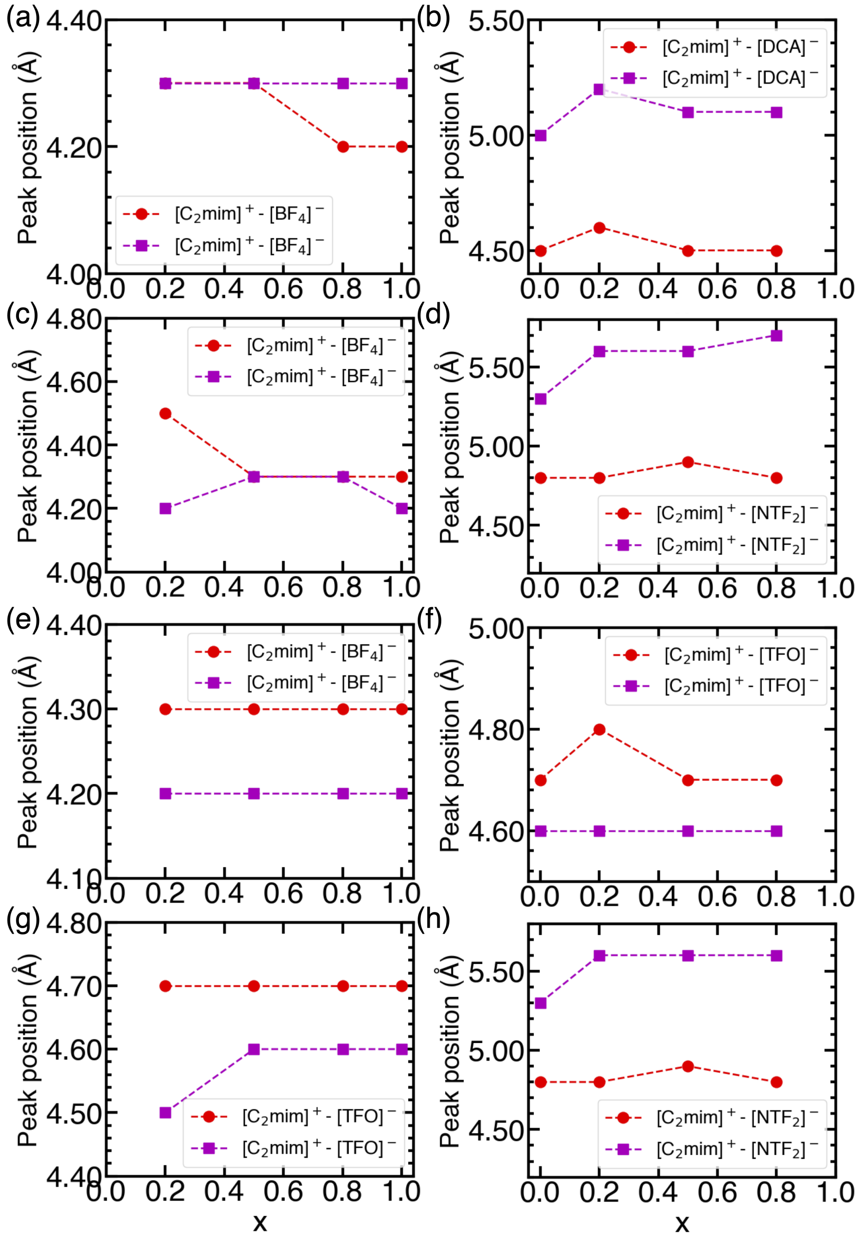

Figure S21: COM-COM cation-anion RDFs first peak position: (a, b) $[C_2mim][BF_4]_x[DCA]_{(1-x)}$, (c, d) $[C_2mim][BF_4]_x[NTF_2]_{(1-x)}$, (e, f) $[C_2mim][BF_4]_x[TFO]_{(1-x)}$ and (g, h) $[C_2mim][TFO]_x[NTF_2]_{(1-x)}$ mixtures with varying concentrations. The colors red and magenta represent VSIL and interpolated-charges.

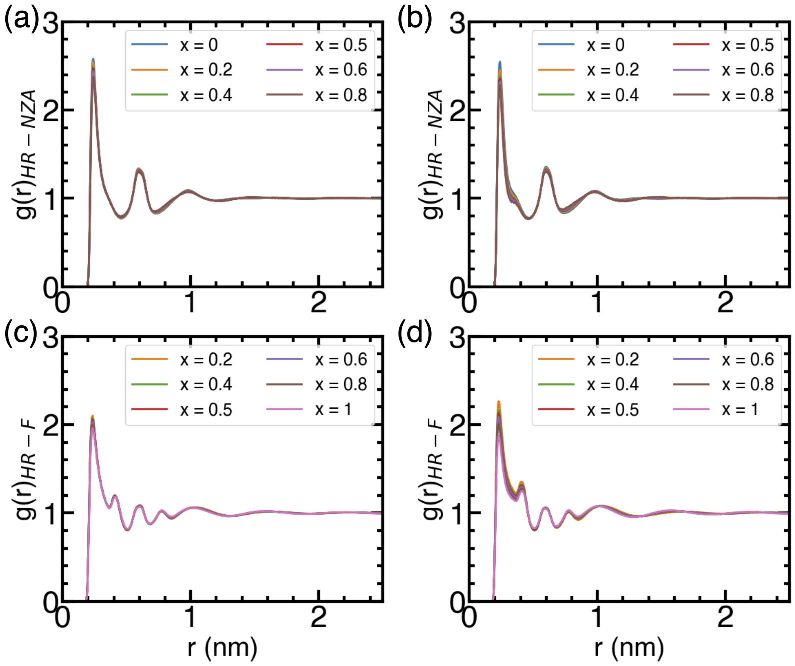

Figure S22: Atom-Atom RDFs depicting the hydrogen bonding interactions in $[C_2mim][BF_4]_x[DCA]_{(1-x)}$ mixture using VSIL (left) and interpolated-charges (right): (a, b) HR-NZA and (c, d) HR-F.

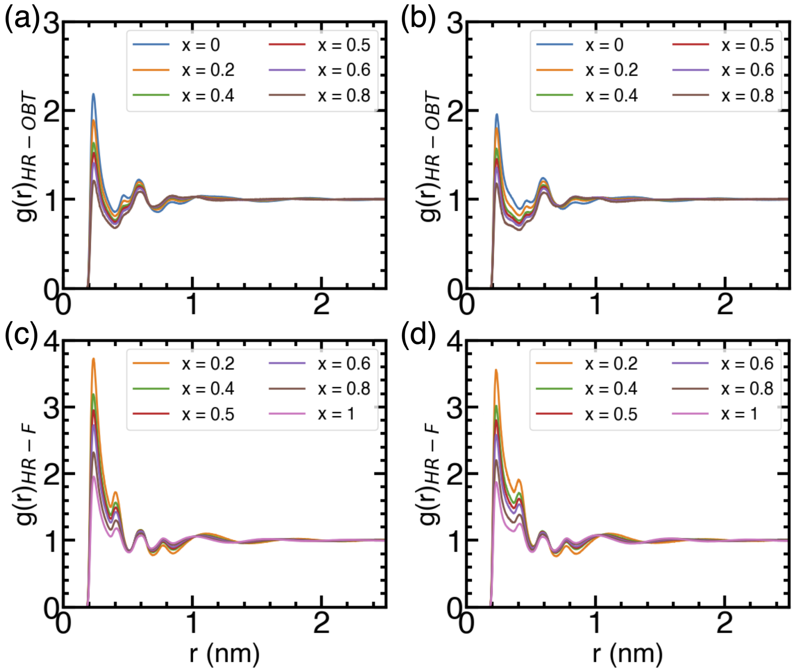

Figure S23: Atom-Atom RDFs depicting the hydrogen bonding interactions in $[C_2mim][BF_4]_x[NTF_2]_{(1-x)}$ mixture using VSIL (left) and interpolated-charges (right): (a, b) HR-OBT and (c, d) HR-F.

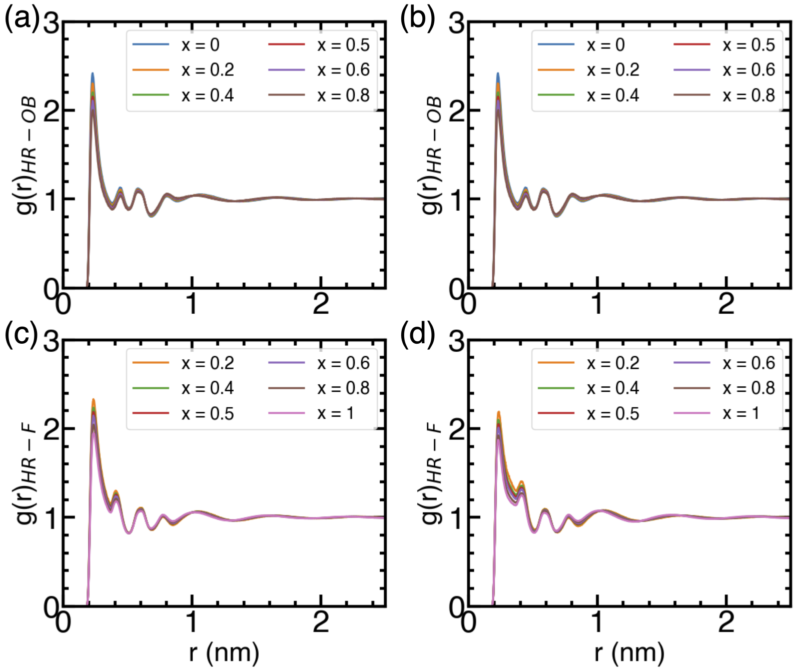

Figure S24: Atom-Atom RDFs depicting the hydrogen bonding interactions in $[C_2mim][BF_4]_x[TFO]_{(1-x)}$ mixture using VSIL (left) and interpolated-charges (right): (a, b) HR-OB and (c, d) HR-F.

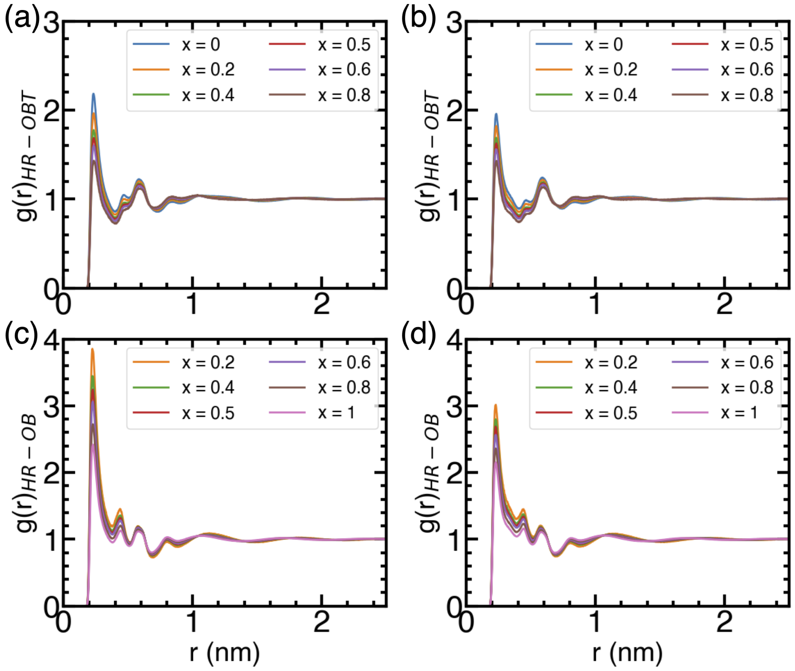

Figure S25: Atom-Atom RDFs depicting the hydrogen bonding interactions in $[C_2mim][TFO]_x[NTF_2]_{(1-x)}$ mixture using VSIL (left) and interpolated-charges (right): (a, b) HR-OB and (c, d) HR-OBT.

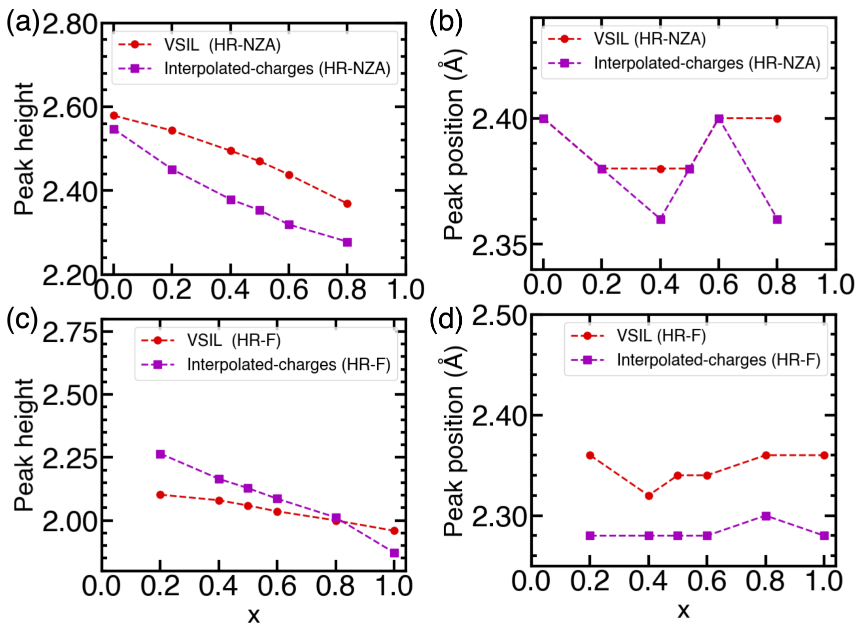

Figure S26: Atom-Atom RDFs first peak height (left) and position (right) in $[C_2mim][BF_4]_x[DCA]_{(1-x)}$ mixtures with varying concentrations: (a, b) HR-NZA and (c, d) HR-F.

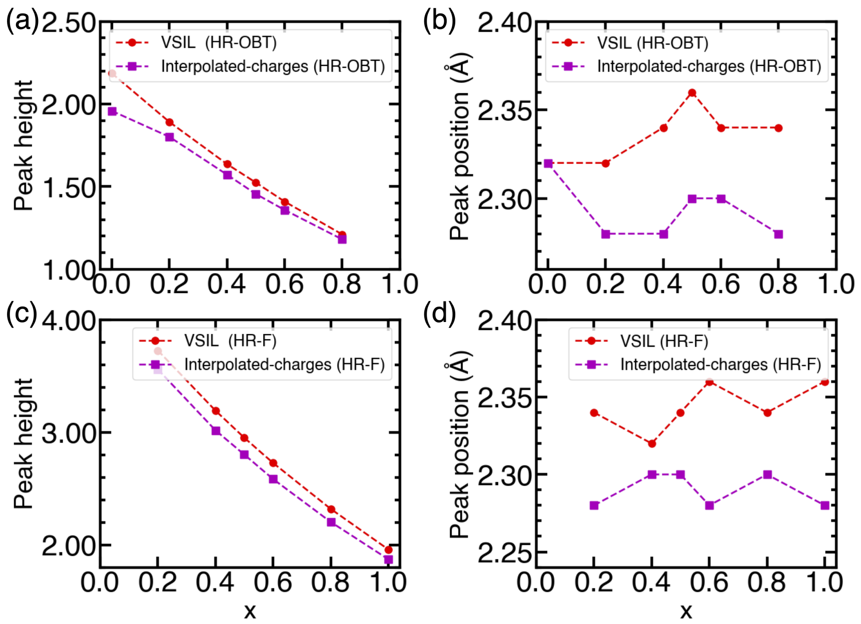

Figure S27: Atom-Atom RDFs first peak height (left) and position (right) in $[C_2mim][BF_4]_x[NTF_2]_{(1-x)}$ mixtures with varying concentrations: (a, b) HR-OBT and (c, d) HR-F.

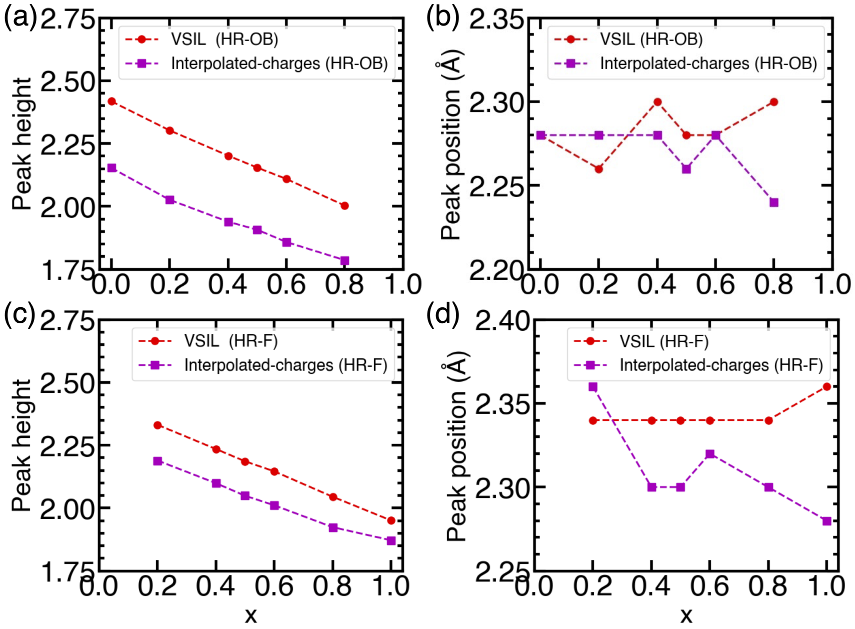

Figure S28: Atom-Atom RDFs first peak height (left) and position (right) in $[C_2mim][BF_4]_x[TFO]_{(1-x)}$ mixtures with varying concentrations: (a, b) HR-OB and (c, d) HR-F.

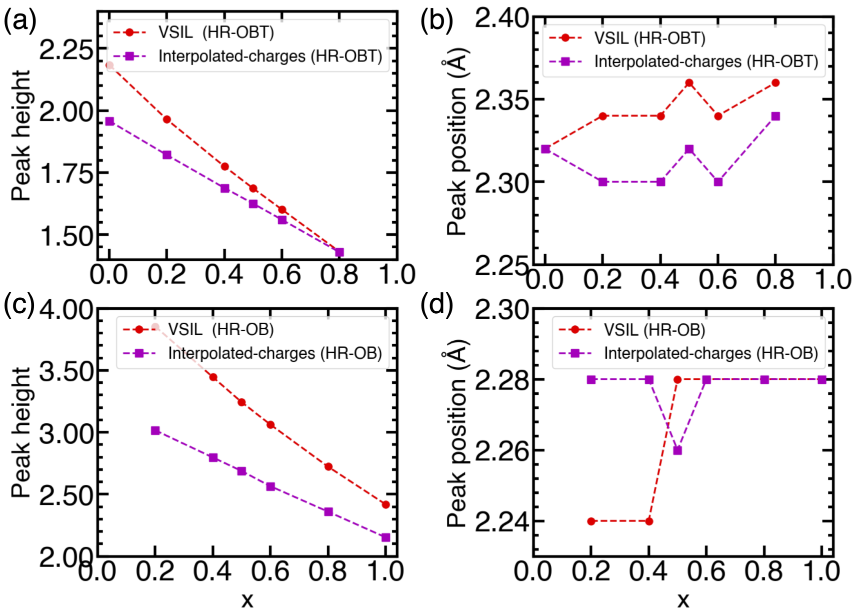

Figure S29: Atom-Atom RDFs first peak height (left) and position (right) in $[C_2mim][TFO]_x[NTF_2]_{(1-x)}$ mixtures with varying concentrations: (a, b) HR-OB and (c, d) HR-OBT.

Table S17: First peak RDF values in $[C_2mim][BF_4]_x[DCA]_{(1-x)}$ mixture: Composition-Dependent Anion Distribution around $[C_2mim]^+$.

x →	0	0.2		0.5		0.8		1
Anions →	$[DCA]^-$	$[DCA]^-$	$[BF_4]^-$	$[DCA]^-$	$[BF_4]^-$	$[DCA]^-$	$[BF_4]^-$	$[BF_4]^-$
cp2k-charges	1.75	1.75	2.59	1.70	2.49	1.64	2.41	2.40
Interpolated-charges	1.75	1.72	2.60	1.68	2.53	1.64	2.44	2.40
VSIL	1.70	1.70	2.39	1.70	2.38	1.71	2.39	2.38

Table S18: First peak RDF values in $[C_2mim][BF_4]_x[NTF_2]_{(1-x)}$ mixture: Composition-Dependent Anion Distribution around $[C_2mim]^+$.

x →	0	0.2		0.5		0.8		1
Anions →	$[NTF_2]^-$	$[NTF_2]^-$	$[BF_4]^-$	$[NTF_2]^-$	$[BF_4]^-$	$[NTF_2]^-$	$[BF_4]^-$	$[BF_4]^-$
cp2k-charges	2.00	1.87	3.76	1.71	3.10	1.52	2.64	2.40
Interpolated-charges	2.00	1.87	3.68	1.67	3.13	1.49	2.66	2.40
VSIL	2.18	2.03	3.38	1.83	3.07	1.63	2.65	2.38

Table S19: First peak RDF values in $[C_2mim][BF_4]_x[TFO]_{(1-x)}$ mixture: Composition-Dependent Anion Distribution around $[C_2mim]^+$.

x →	0	0.2		0.5		0.8		1
Anions →	$[TFO]^-$	$[TFO]^-$	$[BF_4]^-$	$[TFO]^-$	$[BF_4]^-$	$[TFO]^-$	$[BF_4]^-$	$[BF_4]^-$
cp2k-charges	2.34	2.25	2.81	2.18	2.55	2.16	2.45	2.40
Interpolated-charges	2.34	2.27	2.75	2.17	2.58	2.12	2.45	2.40
VSIL	2.36	2.33	2.65	2.27	2.54	2.22	2.45	2.38

Table S20: First peak RDF values in $[C_2mim][TFO]_x[NTF_2]_{(1-x)}$ mixture: Composition-Dependent Anion Distribution around $[C_2mim]^+$.

x →	0	0.2		0.5		0.8		1
Anions →	$[NTF_2]^-$	$[TFO]^-$	$[NTF_2]^-$	$[TFO]^-$	$[NTF_2]^-$	$[TFO]^-$	$[NTF_2]^-$	$[TFO]^-$
cp2k-charges	2.00	2.95	1.91	2.67	1.81	2.45	1.73	2.34
Interpolated-charges	2.00	2.95	1.91	2.72	1.80	2.49	1.67	2.34
VSIL	2.18	2.96	2.08	2.72	1.92	2.50	1.77	2.36

Table S21: Positions of the 1st peak (nm) for RDFs in $[C_2mim][BF_4]_x[DCA]_{(1-x)}$ mixture as a function of compositions.

x →	0	0.2		0.5		0.8		1
r (nm) →	$[C_2mim]^+$-$[DCA]^-$	$[C_2mim]^+$-$[DCA]^-$	$[C_2mim]^+$-$[BF_4]^-$	$[C_2mim]^+$-$[DCA]^-$	$[C_2mim]^+$-$[BF_4]^-$	$[C_2mim]^+$-$[DCA]^-$	$[C_2mim]^+$-$[BF_4]^-$	$[C_2mim]^+$-$[BF_4]^-$
cp2k-charges	0.50	0.52	0.43	0.52	0.43	0.50	0.42	0.42
Interpolated-charges	0.50	0.52	0.43	0.51	0.43	0.51	0.42	0.42
VSIL	0.45	0.46	0.43	0.45	0.43	0.45	0.43	0.43

Table S22: Positions of the 1st peak (nm) for RDFs in $[C_2mim][BF_4]_x[NTF_2]_{(1-x)}$ mixture as a function of compositions.

x →	0	0.2		0.5		0.8		1
r (nm) →	$[C_2mim]^+$-$[NTF_2]^-$	$[C_2mim]^+$-$[NTF_2]^-$	$[C_2mim]^+$-$[BF_4]^-$	$[C_2mim]^+$-$[NTF_2]^-$	$[C_2mim]^+$-$[BF_4]^-$	$[C_2mim]^+$-$[NTF_2]^-$	$[C_2mim]^+$-$[BF_4]^-$	$[C_2mim]^+$-$[BF_4]^-$
cp2k-charges	0.53	0.56	0.43	0.56	0.42	0.57	0.43	0.42
Interpolated-charges	0.53	0.56	0.42	0.56	0.43	0.57	0.43	0.42
VSIL	0.48	0.48	0.45	0.49	0.43	0.48	0.43	0.43

Table S23: Positions of the 1st peak (nm) for RDFs in $[C_2mim][BF_4]_x[TFO]_{(1-x)}$ mixture as a function of compositions.

x →	0	0.2		0.5		0.8		1
r (nm) →	$[C_2mim]^+$-$[TFO]^-$	$[C_2mim]^+$-$[TFO]^-$	$[C_2mim]^+$-$[BF_4]^-$	$[C_2mim]^+$-$[TFO]^-$	$[C_2mim]^+$-$[BF_4]^-$	$[C_2mim]^+$-$[TFO]^-$	$[C_2mim]^+$-$[BF_4]^-$	$[C_2mim]^+$-$[BF_4]^-$
cp2k-charges	0.46	0.43	0.43	0.46	0.42	0.43	0.43	0.42
Interpolated-charges	0.46	0.46	0.42	0.46	0.42	0.46	0.42	0.42
VSIL	0.47	0.48	0.43	0.47	0.43	0.47	0.43	0.43

Table S24: Positions of the 1st peak (nm) for RDFs in $[C_2mim][TFO]_x[NTF_4]_{(1-x)}$ mixture as a function of compositions.

x →	0	0.2		0.5		0.8		1
r (nm) →	$[C_2mim]^+$- $[NTF_2]^-$	$[C_2mim]^+$- $[TFO]^-$	$[C_2mim]^+$- $[NTF_2]^-$	$[C_2mim]^+$- $[TFO]^-$	$[C_2mim]^+$- $[NTF_2]^-$	$[C_2mim]^+$- $[TFO]^-$	$[C_2mim]^+$- $[NTF_2]^-$	$[C_2mim]^+$- $[TFO]^-$
cp2k-charges	0.53	0.46	0.56	0.46	0.56	0.46	0.56	0.46
Interpolated-charges	0.53	0.45	0.56	0.46	0.56	0.46	0.56	0.46
VSIL	0.48	0.47	0.48	0.47	0.49	0.47	0.48	0.47

Table S25: Radius (nm) of the 1st solvation shell (Cation-Anion) in $[C_2mim][BF_4]x[DCA]_{(1-x)}$ mixture as a function of compositions.

x →	0	0.2		0.5		0.8		1
r (nm) →	$[C_2mim]^+$-$[DCA]^-$	$[C_2mim]^+$-$[DCA]^-$	$[C_2mim]^+$-$[BF_4]^-$	$[C_2mim]^+$-$[DCA]^-$	$[C_2mim]^+$-$[BF_4]^-$	$[C_2mim]^+$-$[DCA]^-$	$[C_2mim]^+$-$[BF_4]^-$	$[C_2mim]^+$-$[BF_4]^-$
cp2k-charges	0.74	0.74	0.74	0.74	0.73	0.51	0.74	0.75
Interpolated-charges	0.74	0.74	0.74	0.74	0.74	0.52	0.75	0.75
VSIL	0.74	0.73	0.74	0.74	0.74	0.74	0.75	0.75

Table S26: Radius (nm) of the 1st solvation shell (Cation-Anion) in $[C_2mim][BF_4]_x[NTF_2]_{(1-x)}$ mixture as a function of compositions.

x →	0	0.2		0.5		0.8		1
r (nm) →	$[C_2mim]^+$-$[NTF_2]^-$	$[C_2mim]^+$-$[NTF_2]^-$	$[C_2mim]^+$-$[BF_4]^-$	$[C_2mim]^+$-$[NTF_2]^-$	$[C_2mim]^+$-$[BF_4]^-$	$[C_2mim]^+$-$[NTF_2]^-$	$[C_2mim]^+$-$[BF_4]^-$	$[C_2mim]^+$-$[BF_4]^-$
cp2k-charges	0.88	0.87	0.75	0.86	0.75	0.86	0.75	0.75
Interpolated-charges	0.88	0.87	0.75	0.86	0.75	0.86	0.75	0.75
VSIL	0.88	0.79	0.76	0.87	0.76	0.86	0.75	0.75

Table S27: Radius (nm) of the 1st solvation shell (Cation-Anion) in $[C_2mim][BF_4]_x[TFO]_{(1-x)}$ mixture as a function of compositions.

x →	0	0.2		0.5		0.8		1
r (nm) →	$[C_2mim]^+$-$[TFO]^-$	$[C_2mim]^+$-$[TFO]^-$	$[C_2mim]^+$-$[BF_4]^-$	$[C_2mim]^+$-$[TFO]^-$	$[C_2mim]^+$-$[BF_4]^-$	$[C_2mim]^+$-$[TFO]^-$	$[C_2mim]^+$-$[BF_4]^-$	$[C_2mim]^+$-$[BF_4]^-$
cp2k-charges	0.79	0.79	0.75	0.79	0.75	0.79	0.75	0.75
Interpolated-charges	0.79	0.79	0.75	0.79	0.75	0.79	0.75	0.75
VSIL	0.79	0.79	0.76	0.79	0.75	0.79	0.75	0.75

Table S28: Radius (nm) of the 1st solvation shell (Cation-Anion) in $[C_2mim][TFO]_x[NTF_2]_{(1-x)}$ mixture as a function of compositions.

x →	0	0.2		0.5		0.8		1
r (nm) →	$[C_2mim]^+$-$[NTF_2]^-$	$[C_2mim]^+$-$[TFO]^-$	$[C_2mim]^+$-$[NTF_2]^-$	$[C_2mim]^+$-$[NTF_2]^-$	$[C_2mim]^+$-$[NTF_2]^-$	$[C_2mim]^+$-$[TFO]^-$	$[C_2mim]^+$-$[NTF_2]^-$	$[C_2mim]^+$-$[TFO]^-$
cp2k-charges	0.88	0.80	0.88	0.80	0.87	0.79	0.86	0.79
Interpolated-charges	0.88	0.80	0.52	0.80	0.87	0.79	0.86	0.79
VSIL	0.88	0.80	0.88	0.80	0.87	0.79	0.86	0.79

Table S29: Radius (nm) of the 1st solvation shell ($[C_2mim]^+$-$[C_2mim]^+$) in $[C_2mim][BF_4]_x[DCA]_{(1-x)}$ mixture as a function of compositions.

x →	0	0.2	0.5	0.8	1
cp2k-charges	0.94	0.95	0.98	1.00	1.01
Interpolated-charges	0.94	0.95	0.98	1.01	1.01
VSIL	0.95	0.96	0.99	1.01	1.02

Table S30: Radius (nm) of the 1st solvation shell ($[C_2mim]^+$-$[C_2mim]^+$) in $[C_2mim][BF_4]_x[NTF_2]_{(1-x)}$ mixture as a function of compositions.

x →	0	0.2	0.5	0.8	1
cp2k-charges	0.86	1.22	1.05	1.02	1.01
Interpolated-charges	0.86	1.23	1.04	1.01	1.01
VSIL	1.24	1.08	1.07	1.03	1.02

Table S31: Radius (nm) of the 1st solvation shell ($[C_2mim]^+$-$[C_2mim]^+$) in $[C_2mim][BF_4]_x[TFO]_{(1-x)}$ mixture as a function of compositions.

x →	0	0.2	0.5	0.8	1
cp2k-charges	1.13	1.05	1.02	1.01	1.01
Interpolated-charges	1.13	1.06	1.02	1.02	1.01
VSIL	1.13	1.08	1.04	1.01	1.02

Table S32: Radius (nm) of the 1st solvation shell ($[C_2mim]^+$-$[C_2mim]^+$) in $[C_2mim][TFO]_x[NTF_2]_{(1-x)}$ mixture as a function of compositions.

x →	0	0.2	0.5	0.8	1
cp2k-charges	1.27	1.25	1.21	1.16	1.13
Interpolated-charges	1.27	1.26	1.20	1.17	1.13
VSIL	1.25	1.22	1.17	1.15	1.13

Table S33: Radius (nm) of the 1st solvation shell (Anion-Anion) in $[C_2mim][BF_4]_x[DCA]_{(1-x)}$ mixture as a function of compositions.

x ↓	Anion-Anion RDFs	cp2k-charges	Interpolated-charges	VSIL
0	$[DCA]^-$-$[DCA]^-$	1.11	1.11	1.06
0.2	$[DCA]^-$-$[DCA]^-$	0.81	1.08	1.05
	$[BF_4]^-$-$[DCA]^-$	1.07	1.07	1.04
	$[BF_4]^-$-$[BF_4]^-$	1.04	1.04	1.03
0.5	$[DCA]^-$-$[DCA]^-$	1.08	1.08	1.05
	$[BF_4]^-$-$[DCA]^-$	1.05	1.05	1.04
	$[BF_4]^-$-$[BF_4]^-$	1.04	1.04	1.03
0.8	$[DCA]^-$-$[DCA]^-$	0.58	0.57	0.77
	$[BF_4]^-$-$[DCA]^-$	1.04	1.05	1.04
	$[BF_4]^-$-$[BF_4]^-$	0.69	1.04	1.03
1	$[BF_4]^-$-$[BF_4]^-$	1.04	1.04	1.03

Table S34: Radius (nm) of the 1st solvation shell (Anion-Anion) in $[C_2mim][BF_4]_x[NTF_2]_{(1-x)}$ mixture as a function of compositions.

x ↓	Anion-Anion RDFs	cp2k-charges	Interpolated-charges	VSIL
0	$[NTF_2]^-$-$[NTF_2]^-$	1.24	1.24	1.21
0.2	$[NTF_2]^-$-$[NTF_2]^-$	1.23	1.22	0.72
	$[BF_4]^-$-$[NTF_2]^-$	1.15	1.14	0.70
	$[BF_4]^-$-$[BF_4]^-$	1.05	1.05	1.04
0.5	$[NTF_2]^-$-$[NTF_2]^-$	1.22	1.21	1.19
	$[BF_4]^-$-$[NTF_2]^-$	1.13	1.13	1.12
	$[BF_4]^-$-$[BF_4]^-$	1.04	1.05	1.04
0.8	$[NTF_2]^-$-$[NTF_2]^-$	1.16	1.18	1.00
	$[BF_4]^-$-$[NTF_2]^-$	1.12	1.12	1.11
	$[BF_4]^-$-$[BF_4]^-$	1.04	1.03	1.03
1	$[BF_4]^-$-$[BF_4]^-$	1.04	1.04	1.03

Table S35: Radius (nm) of the 1st solvation shell (Anion-Anion) in $[C_2mim][BF_4]_x[TFO]_{(1-x)}$ mixture as a function of compositions.

x ↓	Anion-Anion RDFs	cp2k-charges	Interpolated-charges	VSIL
0	$[TFO]^-$-$[TFO]^-$	1.10	1.10	0.74
0.2	$[TFO]^-$-$[TFO]^-$	1.10	1.10	0.72
	$[BF_4]^-$-$[TFO]^-$	1.07	1.07	0.70
	$[BF_4]^-$-$[BF_4]^-$	1.04	1.04	1.04
0.5	$[TFO]^-$-$[TFO]^-$	1.10	1.09	0.69
	$[BF_4]^-$-$[TFO]^-$	1.07	1.07	1.06
	$[BF_4]^-$-$[BF_4]^-$	1.04	1.04	1.03
0.8	$[TFO]^-$-$[TFO]^-$	0.69	0.69	0.66
	$[BF_4]^-$-$[TFO]^-$	1.06	1.06	1.06
	$[BF_4]^-$-$[BF_4]^-$	0.68	1.03	1.03
1	$[BF_4]^-$-$[BF_4]^-$	1.04	1.04	1.03

Table S36: Radius (nm) of the 1st solvation shell (Anion-Anion) in $[C_2mim][TFO]_x[NTF_2]_{(1-x)}$ mixture as a function of compositions.

x ↓	Anion-Anion RDFs	cp2k-charges	Interpolated-charges	VSIL
0	$[NTF_2]^-$-$[NTF_2]^-$	1.24	1.24	1.21
0.2	$[TFO]^-$-$[TFO]^-$	1.11	1.11	1.12
	$[NTF_2]^-$-$[TFO]^-$	1.18	1.18	1.17
	$[NTF_2]^-$-$[NTF_2]^-$	1.24	1.23	1.21
0.5	$[TFO]^-$-$[TFO]^-$	1.12	1.11	0.74
	$[NTF_2]^-$-$[TFO]^-$	0.78	0.78	1.16
	$[NTF_2]^-$-$[NTF_2]^-$	1.22	1.22	1.19
0.8	$[TFO]^-$-$[TFO]^-$	1.11	1.11	0.76
	$[NTF_2]^-$-$[TFO]^-$	0.78	0.79	1.15
	$[NTF_2]^-$-$[NTF_2]^-$	0.76	0.94	1.14
1	$[TFO]^-$-$[TFO]^-$	1.10	1.10	0.74

S3.7 Coordination Numbers

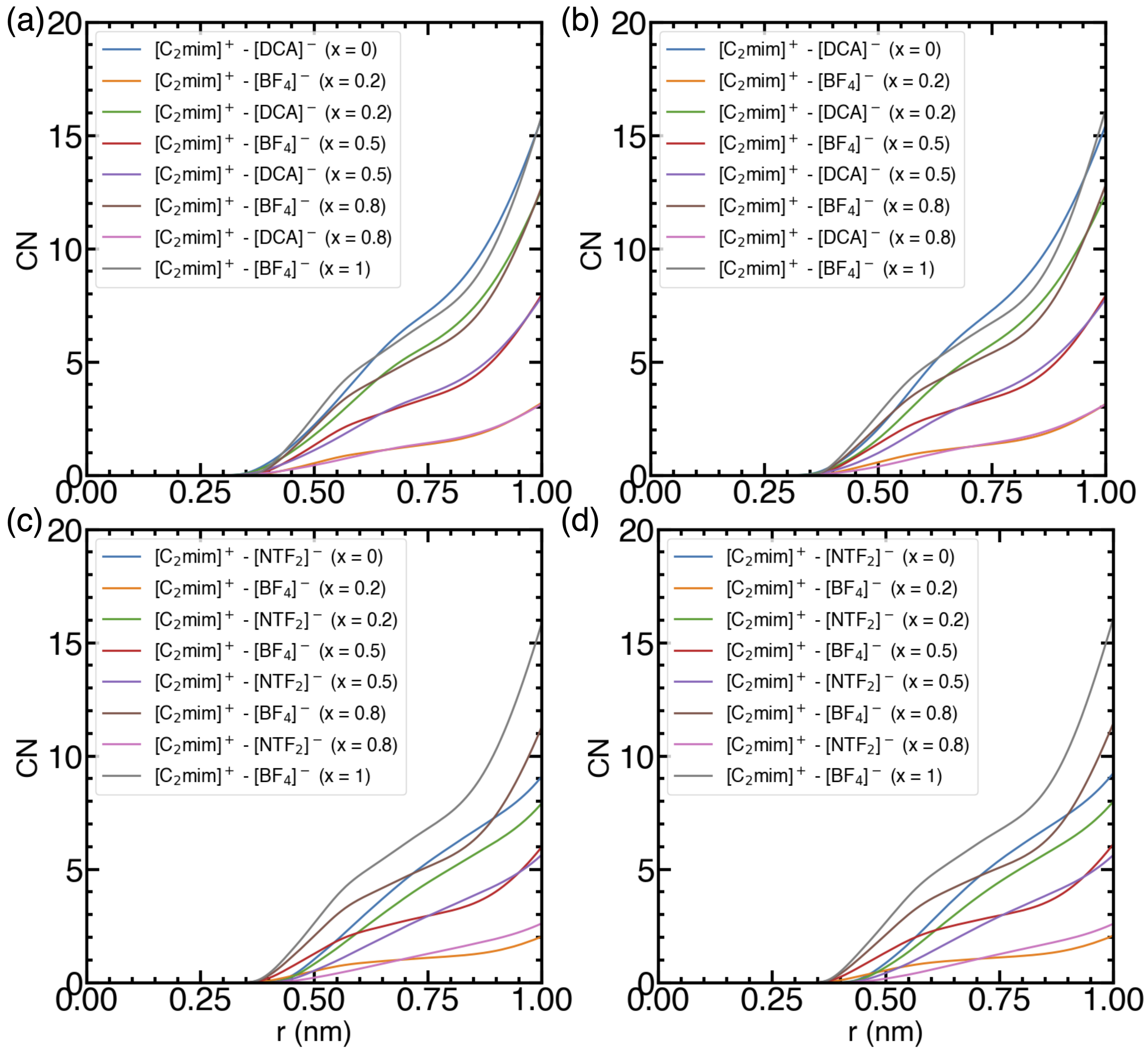

Figure S30: Coordination numbers as a function of radius for (a, b) $[C_2mim][BF_4]_x[DCA]_{(1-x)}$ and (c, d) $[C_2mim][BF_4]_x[NTF_2]_{(1-x)}$ mixtures using VSIL (left) and interpolated-charges (right).

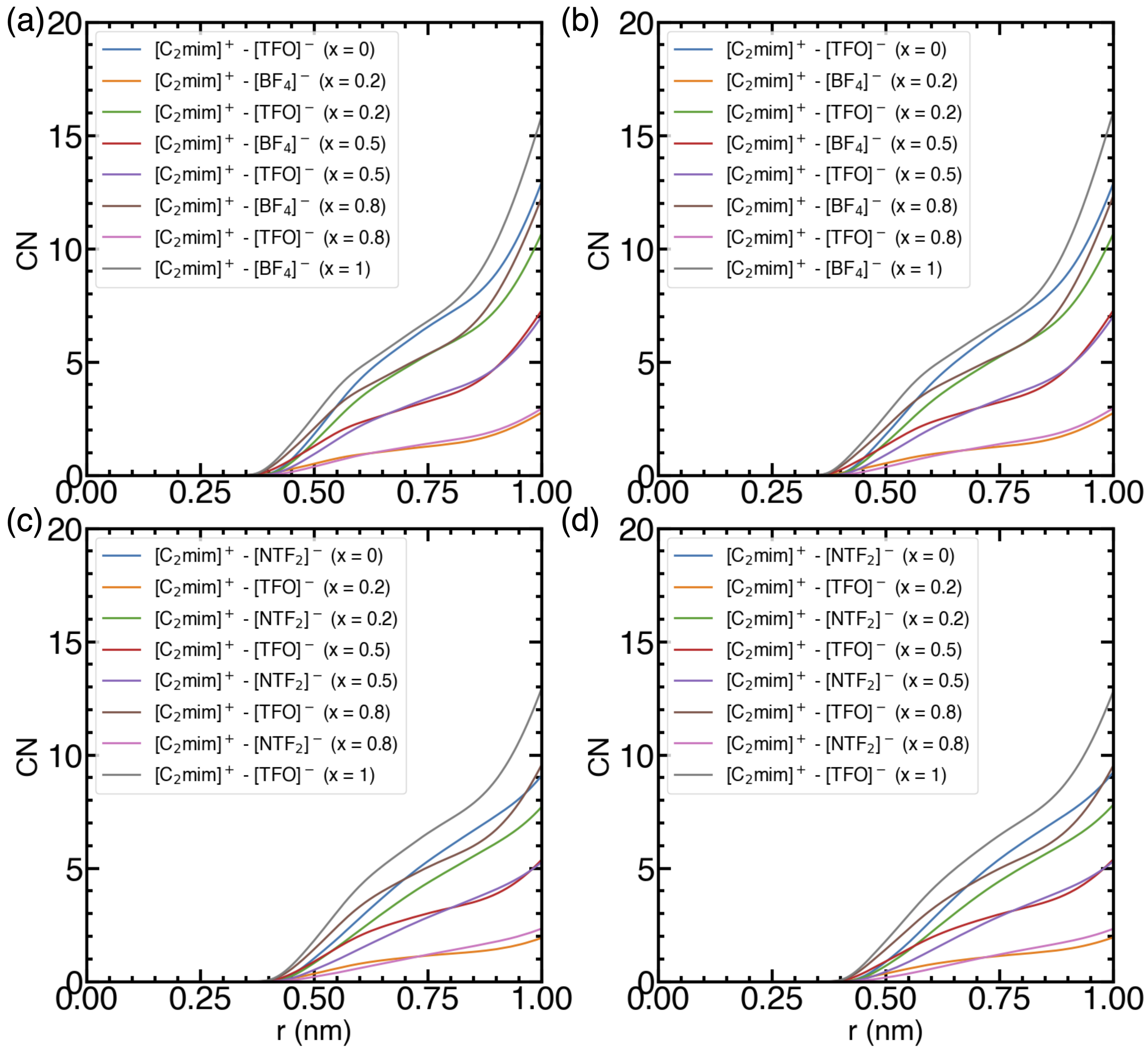

Figure S31: Coordination numbers as a function of radius for (a, b) $[C_2mim][BF_4]_x[NTF_2]_{(1-x)}$ and (c, d) $[C_2mim][TFO]_x[NTF_2]_{(1-x)}$ mixtures using VSIL (left) and interpolated-charges (right).

Table S37: Coordination numbers of $[DCA]^-$ and $[BF_4]^-$ around $[C_2mim]^+$ in $[C_2mim][BF_4]_x[DCA]_{(1-x)}$ mixture: in the first coordination shell of an anion as a function of mixture composition at 298 K. Note that the radius of the first solvation shell is determined from the respective $[C_2mim]^+$-anion RDFs.

x →	0	0.2		0.5		0.8		1
Anions →	$[DCA]^-$	$[DCA]^-$	$[BF_4]^-$	$[DCA]^-$	$[BF_4]^-$	$[DCA]^-$	$[BF_4]^-$	$[BF_4]^-$
cp2k-charges	7.18	5.68	1.35	3.62	3.44	1.44	5.41	6.83
Interpolated-charges	7.18	5.72	1.37	3.60	3.36	1.42	5.44	6.83
VSIL	7.16	5.71	1.37	3.56	3.42	1.43	5.51	6.94

Table S38: Coordination numbers of $[NTF_2]^-$ and $[BF_4]^-$ around $[C_2mim]^+$ in $[C_2mim][BF_4]_x[NTF_2]_{(1-x)}$ mixture: in the first coordination shell of an anion as a function of mixture composition at 298 K. Note that the radius of the first solvation shell is determined from the respective $[C_2mim]^+$-anion RDFs.

x →	0	0.2		0.5		0.8		1
Anions →	$[NTF_2]^-$	$[NTF_2]^-$	$[BF_4]^-$	$[NTF_2]^-$	$[BF_4]^-$	$[NTF_2]^-$	$[BF_4]^-$	$[BF_4]^-$
cp2k-charges	7.32	6.08	1.12	4.05	2.99	1.74	5.15	6.83
Interpolated-charges	7.32	6.08	1.13	4.12	3.02	1.80	5.12	6.83
VSIL	7.30	6.05	1.12	4.14	3.05	1.79	5.22	6.94

Table S39: Coordination numbers of $[TFO]^-$ and $[BF_4]^-$ around $[C_2mim]^+$ in $[C_2mim][BF_4]_x[TFO]_{(1-x)}$ mixture: in the first coordination shell of an anion as a function of mixture composition at 298 K. Note that the radius of the first solvation shell is determined from the respective $[C_2mim]^+$-anion RDFs.

x →	0	0.2		0.5		0.8		1
Anions →	$[TFO]^-$	$[TFO]^-$	$[BF_4]^-$	$[TFO]^-$	$[BF_4]^-$	$[TFO]^-$	$[BF_4]^-$	$[BF_4]^-$
cp2k-charges	7.17	5.78	1.28	3.73	3.28	1.54	5.34	6.83
Interpolated-charges	7.17	5.80	1.28	3.76	3.32	1.53	5.34	6.83
VSIL	7.23	5.89	1.30	3.79	3.34	1.54	5.47	6.94

Table S40: Coordination numbers of $[TFO]^-$ and $[BF_4]^-$ around $[C_2mim]^+$ in $[C_2mim][TFO]_x[NTF_2]_{(1-x)}$ mixture: in the first coordination shell of an anion as a function of mixture composition at 298 K. Note that the radius of the first solvation shell is determined from the respective $[C_2mim]^+$-anion RDFs.

x →	0	0.2		0.5		0.8		1
Anions →	$[NTF_2]^-$	$[TFO]^-$	$[NTF_2]^-$	$[TFO]^-$	$[NTF_2]^-$	$[TFO]^-$	$[NTF_2]^-$	$[TFO]^-$
cp2k-charges	7.32	1.24	6.10	3.27	3.94	5.56	1.68	7.17
Interpolated-charges	7.32	1.24	6.07	3.26	3.90	5.54	1.66	7.17
VSIL	7.30	1.25	5.94	3.30	3.91	5.54	1.65	7.23

S3.8 Spatial Distribution Function

The three-dimensional spatial distribution functions (SDFs) depicted in Figures S32 and S33 were employed to analyze the relative positioning of anions surrounding the cation. These SDFs were generated at isosurface densities corresponding to twice the bulk density. As illustrated in Figure S32, the SDFs for the pure and its mixture ($[C_2mim][BF_4][DCA]$) at three intermediate concentration reveal that the $[BF_4]^-$ interacts with the cation via acidic ring-hydrogen atoms (HR, HW) and the plane between the two imidazolium rings. The SDFs

for the $[C_2mim][BF_4][NTF_2]$ mixture generated using interpolated-charges showed similar to those of the $[C_2mim][BF_4][DCA]$ mixture. However, a notable difference exists at x = 0.8 concentration, where the $[NTF_2]^-$ anion lacks interaction with the HR atom compared to the pure IL. On the other hand, SDFs obtained using the VSIL indicate a weak interaction between the $[NTF_2]^-$ and HR atom at x = 0.8. Notably, the SDFs computed with both interpolated-charges and VSIL for $[C_2mim][BF_4][TFO]$ mixture exhibit high similarity across all concentrations. However, SDFs of $[NTF_2]^-$ around $[C_2mim]^+$ showed a weaker interaction with atom HR at x = 0.2.

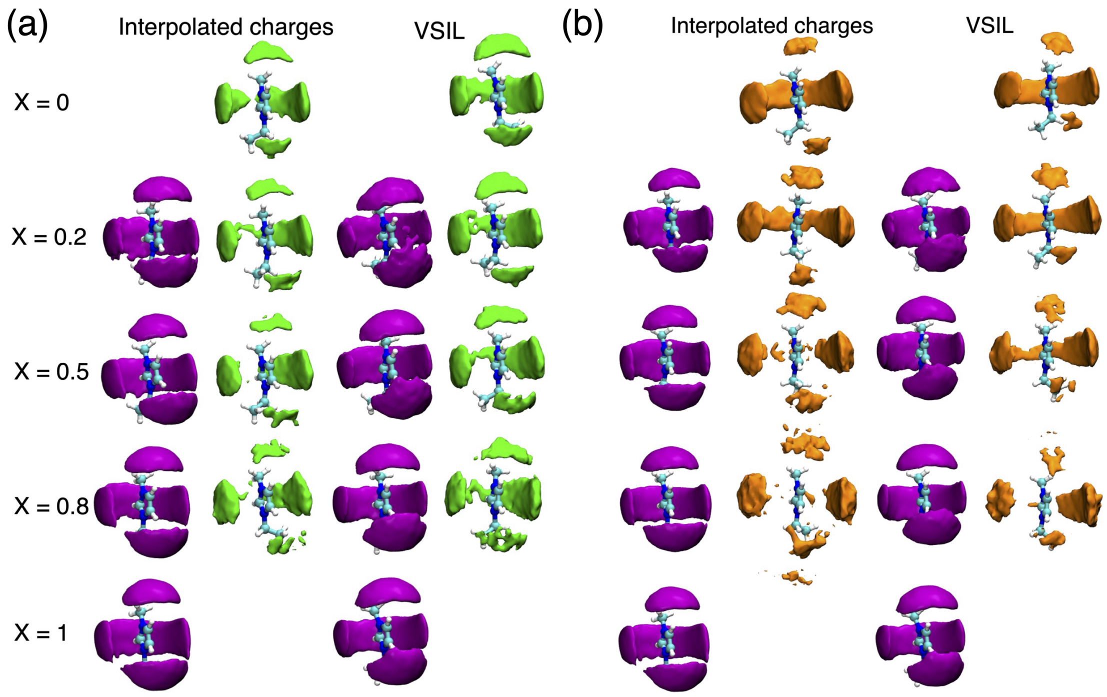

Figure S32: SDFs of $[BF_4]^-$, $[DCA]^-$, and $[NTF_2]^-$ anions around $[C_2mim]^+$ cation in the (a) $[C_2mim][BF_4]_x[DCA]_{(1-x)}$ and (b) $[C_2mim][BF_4]_x[NTF_2]_{(1-x)}$. Color coding: $[BF_4]^-$ in purple, $[DCA]^-$ in green, $[NTF_2]^-$ in orange.

Furthermore, one can see some minor deviations in the bulk structures of $[C_2mim][BF_4][NTF_2]$ and $[C_2mim][TFO][NTF_2]$ mixtures when compared to their pure IL counterparts at low concentrations of $[NTF_2]^-$. These deviations can be due to differences in intermolecular interactions, particularly differences in hydrogen bond ability ($\Delta\beta$) and molar volume (ΔV), which promote the formation of non-native microstructures within the mixtures. The bulk

structure of mixture are expected to be deviate significantly from pure ILs when change in molar volume is > 60 cm^3/mol and change in hydrogen bond ability is > 0.4 as proposed by Kapoor and Shah. As illustrated in Table S41, the range of $\Delta\beta$ is relatively small across ILs. Our findings indicate that binary mixtures containing the $[C_2mim]^+$ cation and exhibiting substantial molar volume changes (60-90 cm^3/mol) but minimal changes in hydrogen bond ability (0.13-0.15), resulting in minor deviations in the bulk structure of $[C_2mim][BF_4][NTF_2]$ and $[C_2mim][TFO][NTF_2]$ at low concentrations of $[NTF_2]^-$.

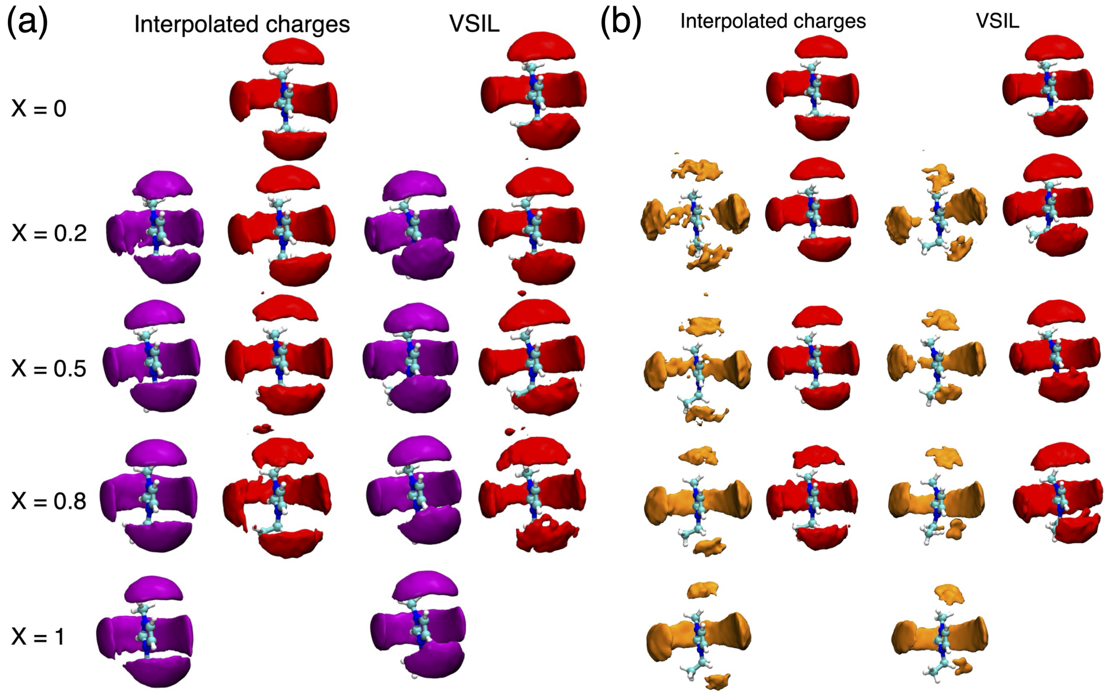

Figure S33: SDFs of $[BF_4]^-$, $[TFO]^-$, and $[NTF_2]^-$ anions around $[C_2mim]^+$ cation in the (a) $[C_2mim][BF_4]_x[TFO]_{(1-x)}$ and (b) $[C_2mim][TFO]_x[NTF_2]_{(1-x)}$. Color coding: $[BF_4]^-$ in purple, $[TFO]^-$ in red, $[NTF_2]^-$ in orange.

Table S41: List of β values for $[C_4mim]^+$-based cation from Spange and co-workers[18] and predicted molar volumes (cc/mol) for all ionic liquids studied in this work.

ILs	β	V_m^{VSIL} (cc/mol)	$V_m^{cp2k-charges}$ (cc/mol)
$[C_2mim][DCA]$	0.64	153.86	155.27
$[C_2mim][BF_4]$	0.55	154.98	154.54
$[C_2mim][TFO]$	0.57	178.87	181.92
$[C_2mim][NTF_2]$	0.42	244.70	245.33

References

(1) Martınez, L.; Andrade, R.; Birgin, E.; Martınez, J. A package for building initial configurations for molecular dynamics simulations. *J. Phys. Chem. Lett* **2009**, *30*, 92157–2164.

(2) Abraham, M. J.; Murtola, T.; Schulz, R.; Páll, S.; Smith, J. C.; Hess, B.; Lindahl, E. GROMACS: High performance molecular simulations through multi-level parallelism from laptops to supercomputers. *SoftwareX* **2015**, *1*, 19–25.

(3) Kühne, T. D.; Iannuzzi, M.; Del Ben, M.; Rybkin, V. V.; Seewald, P.; Stein, F.; Laino, T.; Khaliullin, R. Z.; Schütt, O.; Schiffmann, F.; others CP2K: An electronic structure and molecular dynamics software package-Quickstep: Efficient and accurate electronic structure calculations. *The Journal of Chemical Physics* **2020**, *152*, 194103.

(4) Blöchl, P. Electrostatic decoupling of periodic images of plane-wave-expanded densities and derived atomic point charges. *The Journal of chemical physics* **1995**, *103*, 7422–7428.

(5) Perdew, J. P.; Burke, K.; Ernzerhof, M. Generalized gradient approximation made simple. *Physical review letters* **1996**, *77*, 3865.

(6) VandeVondele, J.; Hutter, J. Gaussian basis sets for accurate calculations on molecular systems in gas and condensed phases. *The Journal of chemical physics* **2007**, *127*, 114105.

(7) Goedecker, S.; Teter, M.; Hutter, J. Separable dual-space Gaussian pseudopotentials. *Physical Review B* **1996**, *54*, 1703.

(8) Grimme, S.; Antony, J.; Ehrlich, S.; Krieg, H. A consistent and accurate ab initio parametrization of density functional dispersion correction (DFT-D) for the 94 elements H-Pu. *The Journal of chemical physics* **2010**, *132*, 154104.

(9) Mondal, A.; Balasubramanian, S. Quantitative prediction of physical properties of imidazolium based room temperature ionic liquids through determination of condensed phase site charges: A refined force field. *The Journal of Physical Chemistry B* **2014**, *118*, 3409–3422.

(10) Doherty, B.; Zhong, X.; Acevedo, O. Virtual site OPLS force field for imidazolium-based ionic liquids. *The Journal of Physical Chemistry B* **2018**, *122*, 2962–2974.

(11) Noda, A.; Hayamizu, K.; Watanabe, M. Pulsed-gradient spin- echo 1H and 19F NMR ionic diffusion coefficient, viscosity, and ionic conductivity of non-chloroaluminate room-temperature ionic liquids. *The Journal of Physical Chemistry B* **2001**, *105*, 4603–4610.

(12) Haskins, J. B.; Bennett, W. R.; Wu, J. J.; Hernández, D. M.; Borodin, O.; Monk, J. D.; Bauschlicher Jr, C. W.; Lawson, J. W. Computational and experimental investigation of Li-doped ionic liquid electrolytes:[pyr14][TFSI],[pyr13][FSI], and [EMIM][BF4]. *The Journal of Physical Chemistry B* **2014**, *118*, 11295–11309.

(13) Borodin, O. Relation between heat of vaporization, ion transport, molar volume, and cation- anion binding energy for ionic liquids. *The Journal of Physical Chemistry B* **2009**, *113*, 12353–12357.

(14) Yang, M. Y.; Merinov, B. V.; Zybin, S. V.; Goddard III, W. A.; Mok, E. K.; Hah, H. J.; Han, H. E.; Choi, Y. C.; Kim, S. H. Transport properties of imidazolium based ionic liquid electrolytes from molecular dynamics simulations. *Electrochemical Science Advances* **2022**, *2*, e2100007.

(15) Pitawela, N. R.; Shaw, S. K. Imidazolium Triflate Ionic Liquids' Capacitance–Potential Relationships and Transport Properties Affected by Cation Chain Lengths. *ACS Measurement Science Au* **2021**, *1*, 117–130.

(16) Kapoor, U.; Shah, J. K. Thermophysical properties of imidazolium-based binary ionic

liquid mixtures using molecular dynamics simulations. *Journal of Chemical & Engineering Data* **2018**, *63*, 2512–2521.

(17) Trenzado, J. L.; Rodríguez, Y.; Gutiérrez, A.; Cincotti, A.; Aparicio, S. Experimental and molecular modeling study on the binary mixtures of [EMIM][BF4] and [EMIM][TFSI] ionic liquids. *Journal of Molecular Liquids* **2021**, *334*, 116049.

(18) Lungwitz, R.; Spange, S. A hydrogen bond accepting (HBA) scale for anions, including room temperature ionic liquids. *New Journal of Chemistry* **2008**, *32*, 392–394.
